# The Δ40p53 isoform inhibits p53-dependent eRNA transcription and enables regulation by signal-specific transcription factors during p53 activation

**DOI:** 10.1371/journal.pbio.3001364

**Published:** 2021-08-05

**Authors:** Cecilia B. Levandowski, Taylor Jones, Margaret Gruca, Sivapriya Ramamoorthy, Robin D. Dowell, Dylan J. Taatjes

**Affiliations:** 1 Department of Biochemistry, University of Colorado, Boulder, Colorado, United States of America; 2 Department of Molecular, Cellular, and Developmental Biology, University of Colorado, Boulder, Colorado, United States of America; 3 BioFrontiers Institute, University of Colorado, Boulder, Colorado, United States of America; 4 Metabolon, Inc., Durham, North Carolina, United States of America; IMBA, AUSTRIA

## Abstract

The naturally occurring Δ40p53 isoform heterotetramerizes with wild-type p53 (WTp53) to regulate development, aging, and stress responses. How Δ40p53 alters WTp53 function remains enigmatic because their co-expression causes tetramer heterogeneity. We circumvented this issue with a well-tested strategy that expressed Δ40p53:WTp53 as a single transcript, ensuring a 2:2 tetramer stoichiometry. Human MCF10A cell lines expressing Δ40p53:WTp53, WTp53, or WTp53:WTp53 (as controls) from the native TP53 locus were examined with transcriptomics (precision nuclear run-on sequencing [PRO-seq] and RNA sequencing [RNA-seq]), metabolomics, and other methods. Δ40p53:WTp53 was transcriptionally active, and, although phenotypically similar to WTp53 under normal conditions, it failed to induce growth arrest upon Nutlin-induced p53 activation. This occurred via Δ40p53:WTp53-dependent inhibition of enhancer RNA (eRNA) transcription and subsequent failure to induce mRNA biogenesis, despite similar genomic occupancy to WTp53. A different stimulus (5-fluorouracil [5FU]) also showed Δ40p53:WTp53-specific changes in mRNA induction; however, other transcription factors (TFs; e.g., E2F2) could then drive the response, yielding similar outcomes vs. WTp53. Our results establish that Δ40p53 tempers WTp53 function to enable compensatory responses by other stimulus-specific TFs. Such modulation of WTp53 activity may be an essential physiological function for Δ40p53. Moreover, Δ40p53:WTp53 functional distinctions uncovered herein suggest an eRNA requirement for mRNA biogenesis and that human p53 evolved as a tetramer to support eRNA transcription.

## Abstract 2

How does Δ40p53, a naturally occurring isoform of p53 that is linked to accelerated aging, alter WTp53 function? Using an innovative approach, this study reveals that Δ40p53 suppresses enhancer RNA transcription and allows other stimulus-specific transcription factors to modulate the p53 transcriptional response.

## Introduction

Transcription factors (TFs) are the primary drivers of cell state and cell physiology [1]. As a testament to their biological importance, an entire population of fibroblasts will form myotubes upon expression of a single TF, MyoD [2]. As a TF, p53 coordinates cellular stress responses and also plays key roles in cancer, aging, and stem cell biology [3–5]. Regulation of p53 function across these diverse biological circumstances involves an array of mechanisms, with well-studied examples that include changes in posttranslational modification [6] or protein stability [5,7,8]. Less well understood are p53 isoforms [9], which represent naturally occurring truncated products that result from alternative splicing from the native TP53 locus or from altered translation of its mRNA. Among the p53 isoforms that have been identified, the Δ40p53 isoform is arguably the most biologically relevant, yet also remains one of the most enigmatic [10].

Isoform Δ40p53 has many aliases such as p44, p53/p47, and ΔNp53; Δ40p53 lacks only the N-terminal 39 amino acids, which encompass the first p53 activation domain (AD1). All other p53 domains, including the oligomerization and DNA-binding domains, are retained. AD1 is a key p53 domain that drives most p53 transcriptional responses in vivo [11,12] and is required for stable recruitment of co-activators such as Mediator and CBP/p300. AD1 is also recognized by the MDM2 protein, an E3 ubiquitin ligase that negatively regulates p53 function through proteasomal degradation [7,8]. In addition to AD1, the p53 N-terminus contains a second activation domain, AD2. Loss of both AD1 and AD2 or mutation of key residues in each domain (L22, W23 in AD1; L53, W54 in AD2) results in complete loss of p53 TF function and resembles a p53 null phenotype [13]. Notably, p53AD2 is capable of activating a subset of p53 transcripts in the presence of L22, W23 AD1 mutations, to support induction of senescence and tumor suppression [13,14].

In the absence of wild-type p53 (WTp53), the Δ40p53 isoform is transcriptionally inactive in vitro, despite forming stable tetramers [15]. Moreover, Δ40p53 expression in a p53 null background does not alter the p53 null phenotype [16]. Thus, Δ40p53 requires WTp53 to impact p53 transcriptional responses and to cause phenotypic changes. This was best exemplified in mouse studies in which truncated or naturally occurring Δ40p53 isoforms were expressed together with WTp53 in roughly a 1:1 ratio [16,17]. These “Δ40p53 + WTp53” mice adopted an accelerated aging phenotype, which, in addition to premature death, involved physiological changes observed with normal aging, such as increased senescence, early-onset osteoporosis, and memory loss [16,18,19].

Whereas the molecular and cellular mechanisms by which Δ40p53 acts to alter WTp53 function remain unclear, it involves formation of heterodimers with WTp53. The Δ40p53 isoform forms mixed Δ40p53:WTp53 tetramers when expressed together with WTp53 [20,21]. Generation of Δ40p53 can occur by alternate splicing; however, the primary mechanism appears to be through alternate translation via an internal ribosomal entry site (IRES) [22,23]. Cellular levels of Δ40p53 have been shown to increase in response to diverse types of stress [24–27], suggesting that Δ40p53 levels naturally fluctuate throughout the life span of an organism. Whereas enforced co-expression of Δ40p53 with WTp53 causes accelerated aging in mice [16], the direct correlation between cellular stress and Δ40p53 levels suggests a mechanism whereby chronic stress may cause transcriptional changes that contribute to mammalian aging [28].

**Fig 1:**
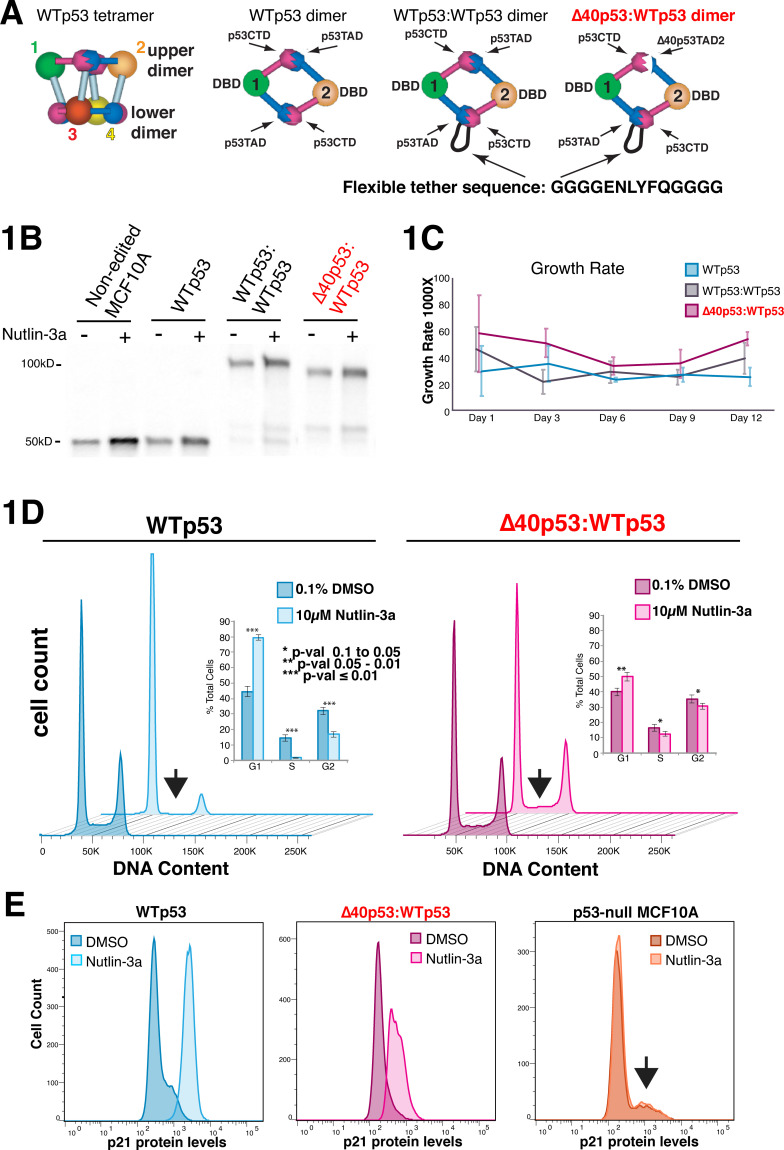
Analysis of Δ40p53:WTp53 tetramers as a single entity; phenotypic comparisons under normal growth and Nutlin-induced conditions. **(A)** Schematic of WTp53 tetramer [29] and strategy to generate Δ40p53:WTp53 tetramers with a fixed 2:2 stoichiometry. Note that the flexible tether is longer than necessary to allow conformational flexibility [15]. **(B)** Western blot to probe p53 levels before (–) or 6 hours after Nutlin-3a treatment. Non-edited MCF10A cells express WTp53 and are shown as a control. Moreover, 20-μg total protein was loaded in each lane. **(C)** Growth rate measured over 5 treatment cycles; each cycle encompassed 20 hours growth under basal (0.1% DMSO) conditions, splitting cells 1:10, then growth for another 48 hours (3 biological replicates; bars = SEM). **(D)** Cell cycle analysis (PI); chart (inset) represents the average of 6 biological replicates (bars = SEM). Arrow highlights loss of S phase in WTp53 cells, in contrast with Δ40p53:WTp53 cells. **(E)** Measurement of p21 protein levels by FACS. Underlying FACS data in the BioStudies database under accession number S-BSST672. Raw data for panels C and D in **S1 Data**. FACS, fluorescence-activated cell sorting; PI, propidium iodide; WTp53, wild-type p53.

A major barrier to understanding how Δ40p53 affects WTp53 function is that co-expression (i.e., Δ40p53 + WTp53) will confound analyses due to tetramer heterogeneity, including formation of “contaminating” WTp53 tetramers. Under such circumstances, the activity of Δ40p53:WTp53 tetramers cannot be decoupled from WTp53 function. The cryo-electron microscopy (cryo-EM) structure of the WTp53 tetramer [29] revealed a straightforward means to link Δ40p53 and WTp53 as a single transcript while preserving p53 tetramer structure (**Fig 1A**). As a proof of principle, we tested the function of tethered Δ40p53:WTp53 tetramers (versus standard WTp53 tetramers) in a series of biochemical and cell-based experiments [15]. As expected, the flexible sequence linking Δ40p53 with WTp53—which was longer than necessary to enable conformational flexibility—did not affect p53 activity. For example, tethered p53 tetramers purified exactly as WTp53 tetramers (e.g., identical over a size-exclusion column), and tethered versions of WTp53 (i.e., WTp53:WTp53) mimicked phenotypic and gene expression changes induced by WTp53 in H1299 cells [15]. In fact, the global gene expression changes (mRNA) induced by WTp53 versus WTp53:WTp53 were essentially identical [15]. These results affirmed the tethering strategy and served as a “proof of concept” for the more rigorous analysis described here, in which we used CRISPR/Cas-9 to generate homozygous knock-in cell lines that expressed WTp53, Δ40p53:WTp53, or WTp53:WTp53 from the native TP53 locus.

Using precision nuclear run-on sequencing (PRO-seq), we measured rapid transcriptional responses following p53 activation, whereas RNA sequencing (RNA-seq) probed subsequent changes in steady-state mRNA levels, and these transcriptional responses were linked to p53 occupancy using chromatin immunoprecipitation sequencing (ChIP-seq). Combined with metabolomics and phenotypic assays, we have better defined how Δ40p53 alters WTp53 function in human cells. Notably, the Δ40p53 isoform tempers WTp53 activity, which allows other TFs to drive stimulus-specific responses. We also uncovered unexpected aspects of Δ40p53 function that link enhancer RNA (eRNA) transcription and mRNA biogenesis and that suggest that 4 complete p53 activation domains (i.e., AD1 + AD2) must occupy a p53 binding site to induce eRNA transcription.

## Results

### Generation of genome-edited cell lines

Three distinct genome-edited (CRISPR/Cas-9) MCF10A cell lines were generated: a WTp53 control, in which WTp53 was simply inserted back into the native TP53 locus; a WTp53:WTp53 control, to probe for potential tether-specific effects; and Δ40p53:WTp53 (**Fig 1A**). In each case, the p53 cDNA sequence was inserted at the first translational start site in exon 2 of the TP53 gene, to ensure expression would be controlled through the native p53 promoter (**S1A and S1B Fig**). We chose MCF10A cells because they endogenously express WTp53 and are derived from non-tumorigenic mammary tissue. MCF10A cells therefore have a stable genome that is not prone to mutations or polyploidy. Edited cells were sorted based on mCherry selection, and single cell clones were expanded and verified homozygous using PCR (**S1C Fig**), western blot (**S1D Fig**), and sequencing (**S2 Fig**).

The endogenous regulation of p53 expression in genome-edited cells was tested by treating cells with the small molecule Nutlin-3a, which disrupts the p53 interaction with MDM2 [30]. Consequently, Nutlin-3a activates p53 and increases its protein levels. As shown in **Fig 1B**, Nutlin-3a increased p53 protein levels in all 3 genome-edited cell lines, confirming each was regulated similar to WTp53 in non-edited MCF10A cells. Additional verification results are shown in **S3 Fig**. Together, these 3 MCF10A cell lines provided a means to evaluate Δ40p53 function under physiologically relevant conditions. Because Δ40p53:WTp53 was expressed as a single transcript, a fixed 2:2 tetramer stoichiometry was assured, avoiding tetramer heterogeneity that results from Δ40p53 + WTp53 co-expression.

### WTp53 and Δ40p53:WTp53 cells are phenotypically similar under normal growth conditions

In non-stressed conditions, cellular p53 activity is typically very low. All 3 cell lines (WTp53, WTp53:WTp53, and Δ40p53:WTp53) were derived from the same parental MCF10A line and were therefore isogenic except at the TP53 locus. Under normal growth conditions, no significant change in cell cycle was observed between WTp53, WTp53:WTp53, or Δ40p53:WTp53 cells (**S4A Fig**). Furthermore, the growth rate of each cell line was similar (**Fig 1C**); whereas growth was slightly enhanced in Δ40p53:WTp53 cells, the increase was not statistically significant. As a tumor suppressor, p53 significantly impacts cell metabolism [5]. Therefore, we compared the metabolomes of WTp53, WTp53:WTp53, and Δ40p53:WTp53 cells. Consistent with the cell cycle and cell growth assays, untargeted metabolomics experiments confirmed that each p53 knock-in cell line had similar (but not identical) basal levels of metabolites (**S1 Table**). Metabolites relevant to cell cycle progression are shown in **S4B Fig**.

### Nutlin-3a exposes phenotypic changes in Δ40p53:WTp53 cells (versus WTp53)

We next examined how each cell line (WTp53, WTp53:WTp53, or Δ40p53:WTp53) would respond to p53 activation by Nutlin-3a. Notably, Nutlin-3a is non-genotoxic and highly specific for p53 [31]; thus, unlike a typical physiological stimulus (e.g., DNA damage), auxiliary pathways are not activated by Nutlin-3a. As shown in **Fig 1D**, p53 activation by Nutlin-3a triggered a marked increase in G1 and a decrease in S and G2 phases in WTp53 cells. These results are characteristic of G1 arrest and represent a typical p53 response [3]. Also as expected, WTp53:WTp53 cells were similar to WTp53 cells (**S5A Fig**). By contrast, cell cycle data for Nutlin-treated Δ40p53:WTp53 cells resembled control-treated (DMSO) cells, with only a modest Nutlin-dependent shift in G1 that did not reach statistical confidence of *p* ≤ 0.01 (**Fig 1D**).

A p53 target gene that drives cell cycle arrest is CDKN1A (aka p21), and p21 can be induced by mixed Δ40p53:WTp53 tetramers, as shown by us [15] and others [16]. We measured p21 protein levels in Nutlin-treated WTp53 versus Δ40p53:WTp53 cells and observed increases in each line (**Fig 1E**; WTp53:WTp53 in **S5B Fig**). Although p21 protein levels were greater in WTp53 cells, it was evident that Nutlin-3a activated Δ40p53:WTp53 tetramers, consistent with increased Δ40p53:WTp53 protein levels upon Nutlin-3a treatment (**Fig 1B**). Parallel experiments in p53 null MCF10A cells confirmed that the Nutlin-dependent increase in p21 protein was p53 dependent (**Fig 1E**). Also, as expected, cell cycle changes were not observed in p53 null cells in response to Nutlin-3a treatment, nor was there a change in p21 mRNA levels (**S5C Fig**).

Others have shown that cell stress transiently increases Δ40p53 protein levels [20,24–26,32], and this has implications for aging, as chronic induction of Δ40p53 over time may contribute to physiological aging. To simulate transient periods of cell stress (i.e., p53 pathway activation), we subjected each cell line (WTp53, WTp53:WTp53, or Δ40p53:WTp53) to repeated cycles of Nutlin-3a treatment (20 hours), followed by 48 hours recovery. As shown in **S5D Fig**, WTp53:WTp53 cells responded similarly to WTp53, as expected. By contrast, a stark difference was observed between WTp53 and Δ40p53:WTp53 cells. Growth was arrested in WTp53 cells, but proliferation continued in Δ40p53:WTp53 cells, even after multiple rounds of Nutlin treatment (**S5D Fig**). This result contrasts with untreated (DMSO) WTp53 versus Δ40p53:WTp53 cells, which showed no significant difference in growth rate (**Fig 1C**).

Because the metabolic needs for proliferating versus arrested cells will be distinct, we also compared the metabolomes of WTp53 versus Δ40p53:WTp53 cells following Nutlin-3a treatment (**S2 Table**). As expected, Nutlin-treated WTp53 cells showed metabolic changes that reflected their reduced proliferation (**S2 Table**; similar results in WTp53:WTp53 cells). By contrast, metabolic differences evident in Δ40p53:WTp53 cells were consistent with their maintenance of proliferation and were observed even prior to Nutlin stimulation (**S1 Table**). For instance, decreased levels of sphingosine or increased levels of sphingomyelin metabolites in Δ40p53:WTp53 cells (**S6 Fig**) is each independently consistent with maintenance of cell cycle and proliferation [33]. Conversely, WTp53 and WTp53:WTp53 cells showed the opposite trends in these metabolites (versus Δ40p53:WTp53), consistent with the induction of cell cycle arrest upon Nutlin treatment. Although these results could reflect a complete lack of p53 pathway activation by Nutlin-3a in Δ40p53:WTp53 cells, this did not appear to be the case based upon data shown in **Fig 1B** (Nutlin-dependent increase in Δ40p53:WTp53 levels) or **Fig 1E** (p21 protein induction). To further probe the underlying mechanisms, we next assessed how Nutlin-dependent p53 activation affected the transcriptomes of Δ40p53:WTp53 versus WTp53 cells.

### Δ40p53:WTp53 differentially affects the pol II transcriptome upon Nutlin-3a treatment

To compare and contrast transcriptional changes in Δ40p53:WTp53 cells (versus WTp53), we used PRO-seq, which measures nascent transcription genome-wide [34]. PRO-seq detects transcripts from all 3 RNA polymerases and measures all types of pol II transcripts, including noncoding RNAs and non-annotated regions. Cells (Δ40p53:WTp53, WTp53, and WTp53:WTp53) were treated with Nutlin-3a for 3 hours, and differential transcription was quantified using DEseq2 [35]. The 3-hour time point was determined empirically (**S7 Fig**), with reference to Nutlin-treated HCT116 cells that we evaluated previously [36]. This early time point following Nutlin stimulation favored identification of direct (i.e., primary) p53 transcriptional targets.

**Fig 2:**
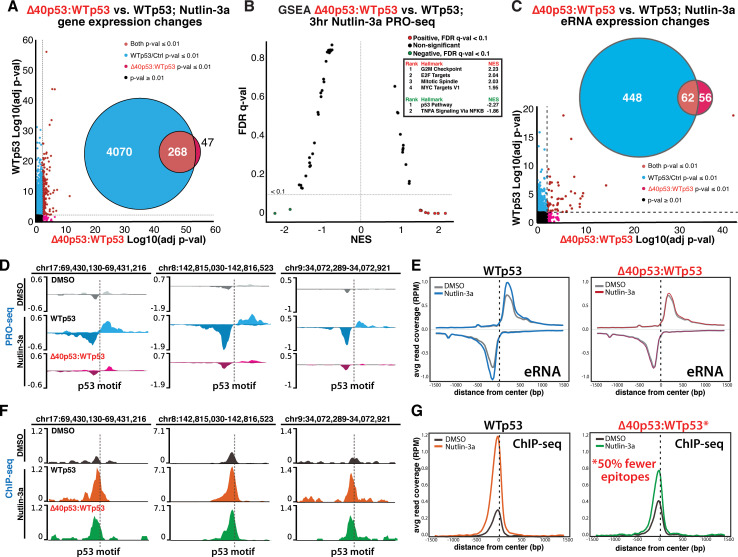
Δ40p53 alters WTp53 function; Δ40p53:WTp53 fails to induce eRNA transcription despite similar genomic occupancy vs. WTp53. **(A)** Summary of PRO-seq data for WTp53 (y-axis) vs. Δ40p53:WTp53 (x-axis). Dashed line represents *p*-value 0.01. Venn diagram shows overlap between WTp53 and Δ40p53:WTp53 cells. **(B)** GSEA based upon PRO-seq data (3 hours Nutlin-3a) comparing Δ40p53:WTp53 vs. WTp53. Pathways with FDR (q-val <0.1) are colored dots. Red represents increased in Δ40p53:WTp53 compared to WTp53, and green is decreased. X-axis is NES. Top significant pathways are in the ranked list. **(C)** Summary of PRO-seq data of eRNA transcription for WTp53 (y-axis) vs. Δ40p53:WTp53 (x-axis). Dashed line represents *p*-value 0.01. Venn diagram shows overlap between WTp53 and Δ40p53:WTp53 cells. **(D)** Examples of PRO-seq data, showing Nutlin-induced eRNA transcription in WTp53 cells, but not Δ40p53:WTp53. Location of p53 binding motif (*p*-val ≤ 1 × 10^−5^) indicated with dashed line. (**E)** Metagene analysis showing average eRNA peak height, genome-wide, at p53-responsive eRNAs (*p*-val < 0.25) in WTp53 or Δ40p53:WTp53 cells. **(F)** Examples of ChIP-seq data in Nutlin-treated (6 hours) WTp53 or Δ40p53:WTp53 cells, compared with DMSO controls; aligns with PRO-seq locations from panel D. **(G)** Metagene analyses showing average ChIP-seq signal, genome-wide, at p53 binding sites in control vs. Nutlin-treated WTp53 or Δ40p53:WTp53 cells. Raw data for panels A–C, E, and G in **S2 Data**. ChIP-seq, chromatin immunoprecipitation sequencing; eRNA, enhancer RNA; FDR, false discovery rate; GSEA, gene set enrichment analysis; NES, normalized enrichment score; PRO-seq, precision nuclear run-on sequencing; WTp53, wild-type p53.

In WTp53 cells, 4,338 annotated regions were differentially transcribed (*p*-value ≤ 0.01) after Nutlin-3a treatment (**Fig 2A**), with similar transcriptional changes in WTp53:WTp53 cells (**S8 Fig**). By contrast, only 315 annotated regions were differentially transcribed (*p*-value ≤ 0.01) in Δ40p53:WTp53 cells after Nutlin-3a treatment (**Fig 2A**). Of these 315 Nutlin-induced transcripts in Δ40p53:WTp53 cells, 268 were also observed in WTp53 cells, and 47 annotated genes were differentially transcribed in Δ40p53:WTp53 versus WTp53 cells (**S9A Fig**), with most linked to cell growth. We confirmed that a significant p53 response was occurring in Δ40p53:WTp53 cells by completing parallel PRO-seq experiments in p53 null MCF10A cells (**S9B–S9D Fig**). Thus, the magnitude of Nutlin-3a induction was simply greater in WTp53 cells versus Δ40p53:WTp53, with examples shown in **S9E Fig**.

Gene set enrichment analysis (GSEA) of the Nutlin-induced genes revealed substantial differences, with pathways associated with growth and cell cycle progression increased in Δ40p53:WTp53 cells (**Fig 2B**). Enrichment of the E2F pathway in Nutlin-treated Δ40p53:WTp53 cells (versus WTp53) was notable because the E2F TF family has overlapping function with p53 [37]. The reduced p53 pathway activity (GSEA, **Fig 2B**) was consistent with dampened response in Δ40p53:WTp53 cells compared with WTp53. An integrated pathway analysis of the Nutlin-induced genes showed results consistent with GSEA, with growth and cell cycle pathways enhanced in Δ40p53:WTp53 cells (**S9F Fig**). In particular, mammalian target of rapamycin (mTOR) signaling and insulin-like growth factor 1 (IGF-1) signaling were increased in Δ40p53:WTp53 cells compared to WTp53; each of these pathways has been linked to Δ40p53 expression in human cells [15] or mouse models [16], as potential contributors to the Δ40p53 accelerated aging phenotype.

Although the transcriptional changes summarized in **S9A Fig** may contribute to the increased proliferation of Δ40p53:WTp53 cells (versus WTp53) after Nutlin-3a treatment (**S5D Fig**), we emphasize that the PRO-seq data were obtained after only 3-hour treatment; changes in steady-state mRNA levels (i.e., RNA-seq) after a longer period of Nutlin-3a treatment are described later.

### Δ40p53:WTp53 tetramers fail to induce eRNA transcription

In addition to gene expression changes in annotated regions, the PRO-seq data revealed stark differences in transcription of eRNAs. Whereas WTp53 cells had 510 differentially transcribed (*p*-value ≤ 0.01) eRNAs after 3-hour Nutlin-3a treatment, only 118 eRNAs changed in Δ40p53:WTp53 cells. Among the 510 WTp53 eRNAs (**Fig 2C**), 109 mapped to p53 binding sites identified by ChIP-seq (see below; similar results in WTp53:WTp53 cells, **S10 Fig**). These 109 p53-associated eRNAs are indicative of direct eRNA activation by p53 [36,38]. By contrast, only 6 eRNAs mapped to p53 binding sites in Δ40p53:WTp53 cells, revealing a defect in its ability to induce eRNA transcription. The examples shown in **Fig 2D** and **S11 Fig** further highlight the contrast in eRNA induction between WTp53 and Δ40p53:WTp53 tetramers. We note that Nutlin-induced eRNAs that mapped to sites not bound by p53 likely represent weak p53 binding (i.e., below cutoff) or secondary effects from p53 activation.

Whereas the biological roles for eRNA transcription remain unclear, they display cell type–specific expression patterns [39] and rapidly respond to external stimuli. Most relevant to this study, eRNAs are transcribed in response to p53 binding events [36] and correlate with expression of p53 target genes [38]. To further probe eRNA transcriptional changes, we applied transcription factor enrichment analysis (TFEA), an improved computational method [40] that can detect changes in bidirectional eRNA transcription. TFEA then maps these eRNAs to the genome and identifies any underlying TF binding motifs. In this way, TFEA can accurately identify which TFs are being activated or repressed during a stimulus. As shown in **S12A Fig**, TFEA revealed a robust p53 activation in Nutlin-treated WTp53 cells (and WTp53:WTp53 cells, **S12B Fig**), as expected. In Δ40p53:WTp53 cells, however, no evidence of p53 activation was apparent (**S12C Fig**); moreover, a TFEA comparison of WTp53 versus Δ40p53:WTp53 cells indicated a defect in p53 activation by Δ40p53:WTp53 tetramers (**S12D Fig**). Metagene analyses further illustrated the contrast between eRNA transcription in Nutlin-treated WTp53 versus Δ40p53:WTp53 cells (**Fig 2E**; WTp53:WTp53 data in **S12E Fig**). In agreement with the TFEA results, ChIP-seq analyses confirmed that Δ40p53:WTp53 binding fails to induce eRNA transcription, in direct contrast to WTp53 (see below). Collectively, these results establish that Δ40p53:WTp53 tetramers fail to induce eRNA transcription upon p53 activation.

### Genomic occupancy of Δ40p53:WTp53 is identical to WTp53

Because eRNA induction is dependent upon TF binding [36,41,42], it was possible that eRNAs were not impacted in Δ40p53:WTp53 cells because Δ40p53:WTp53 did not bind p53 sequences, even after Nutlin treatment. However, Δ40p53 contains the entire DNA-binding domain (residues 101 to 300); therefore, we expected that the genomic occupancy of Δ40p53:WTp53 tetramers would resemble that of WTp53. As shown in **Fig 2F** and **S13A and S13B Fig**, ChIP-seq data revealed that Δ40p53:WTp53 bound the same sites as WTp53, genome-wide, as expected given their identical DNA-binding domains. The corresponding eRNAs are shown in **Fig 2D**. Consistent with TFEA results (**S12C Fig**) these data show that Δ40p53:WTp53 binding fails to induce eRNA transcription, in contrast to WTp53. Metagene analyses showed that occupancy of both WTp53 and Δ40p53:WTp53 increased after Nutlin-3a treatment, as expected (**Fig 2G**; WTp53:WTp53 shown in **S13C Fig**). No Δ40p53:WTp53-specific binding sites were detected.

Note that the antibody used for ChIP-seq recognizes the p53 N-terminus, which is absent in Δ40p53; thus, Δ40p53:WTp53 possesses 50% fewer epitopes per tetramer, and this will reduce its overall signal. Nevertheless, occupancies of WTp53:WTp53 and Δ40p53:WTp53 appeared nearly identical under basal or Nutlin-treated conditions (**S13B Fig**). Because ChIP-seq is qualitative, we cannot draw conclusions about the relative levels of p53 binding across the cell lines. Moreover, proteins with increased molecular weight (e.g., WTp53:WTp53) are more susceptible to degradation during sonication [43], which may have decreased ChIP efficiency in tethered p53 cell lines. Despite these caveats, it was evident that (1) Δ40p53:WTp53 was appropriately mobilized in response to Nutlin-3a treatment, similar to WTp53; that (2) Δ40p53:WTp53 occupied the same p53 binding sites (versus WTp53), genome-wide, under basal and Nutlin-induced conditions; and that (3) unlike WTp53, Δ40p53:WTp53 binding fails to induce eRNA transcription.

### RNA-seq data suggest defective mRNA biogenesis for transcripts induced by Δ40p53:WTp53

**Fig 3:**
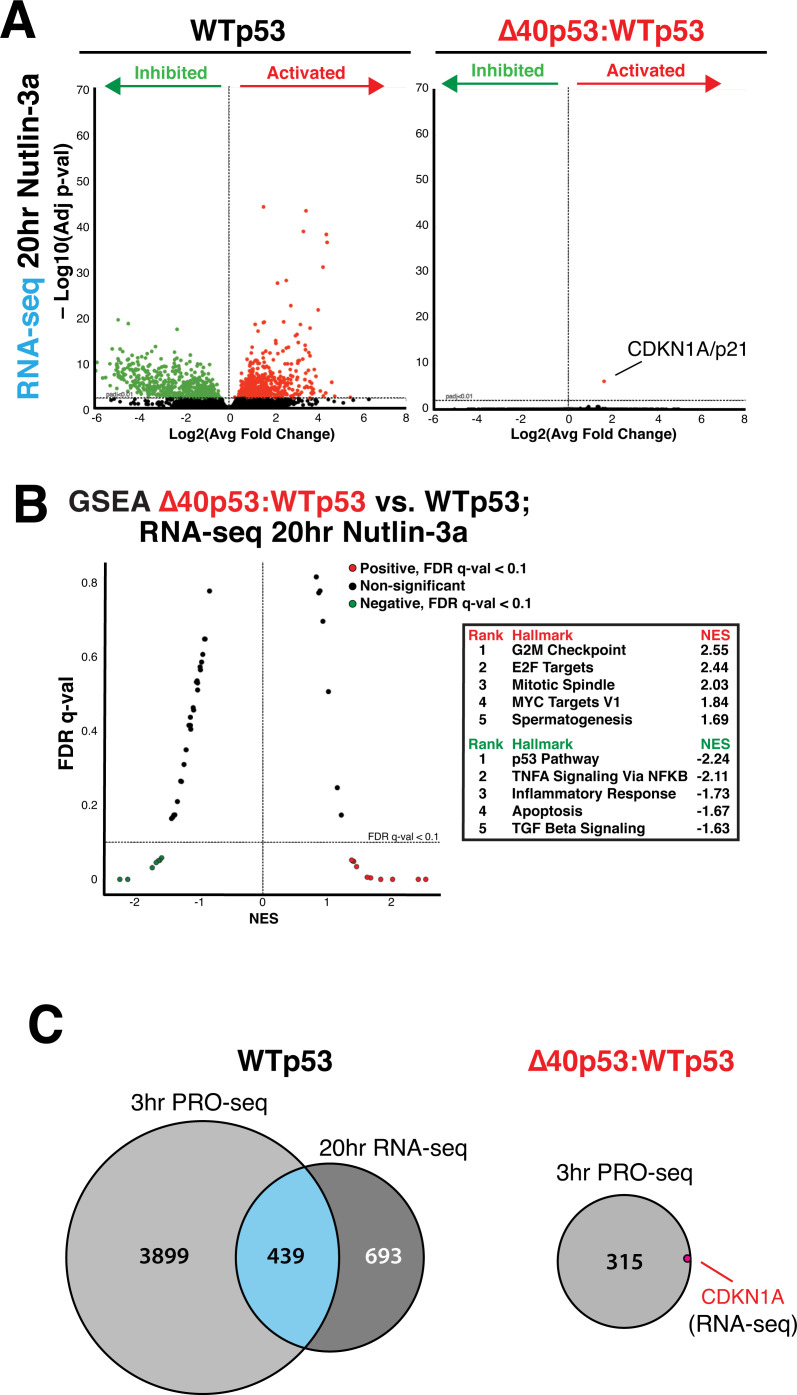
p53 activation fails to increase mRNA levels in Δ40p53:WTp53 cells. **(A)** Volcano plots showing differentially expressed mRNAs after 20-hour Nutlin treatment (vs. DMSO controls) in WTp53 or Δ40p53:WTp53 cells. Green dots represent down-regulated and red dots up-regulated transcripts (*p*-val ≤ 0.01). (**B)** GSEA based upon RNA-seq data (20 hours Nutlin-3a) comparing Δ40p53:WTp53 vs. WTp53. Pathways with FDR q-val <0.1 are colored dots. Red represents increased in Δ40p53:WTp53 compared to WTp53, and green is decreased. X-axis is NES. Top significant pathways are shown in ranked list. **(C)** Venn diagrams showing overlap among significantly induced transcripts from PRO-seq data (3 hours Nutlin) and RNA-seq data (20 hours Nutlin). Whereas about 10% of nascent transcripts show corresponding increases at the mRNA level in WTp53 cells, only CDKN1A/p21 shows this behavior in Δ40p53:WTp53 cells (1/316; 0.3%). Raw data for panels A and B in **S3 Data**. FDR, false discovery rate; GSEA, gene set enrichment analysis; NES, normalized enrichment score; PRO-seq, precision nuclear run-on sequencing; RNA-seq, RNA sequencing; WTp53, wild-type p53.

We next completed biological replicate RNA-seq experiments, to compare cellular mRNA levels with the nascent RNA changes identified by PRO-seq. We treated each cell line (WTp53, Δ40p53:WTp53, and WTp53:WTp53) with Nutlin-3a for 20 hours. This time point was chosen to allow time for Nutlin-induced changes to manifest in the steady-state mRNA transcriptome. As shown in **Fig 3A**, 1,132 genes were differentially expressed (*p*-value ≤ 0.01) in WTp53 cells, with similar results in WTp53:WTp53 cells (**S14A Fig**). In stark contrast, Δ40p53:WTp53 cells showed essentially no Nutlin response at the mRNA level; CDKN1A/p21 was the only gene that was induced (**Fig 3A**). The lack of p53 activation in Δ40p53:WTp53 cells is further highlighted by the heat map of p53 pathway genes, shown in **S14B Fig**. As with the PRO-seq data (**Fig 2B**), GSEA identified the same pathways upon comparison of RNA-seq data from Nutlin-treated Δ40p53:WTp53 versus WTp53 cells (**Fig 3B**). For instance, the inhibited (p53 pathway) and activated (G2M checkpoint, E2F targets) pathways were consistent, again supporting the enhanced proliferation of Δ40p53:WTp53 cells during Nutlin treatment (**S5D Fig**).

As shown in **Fig 3C**, approximately 10% of genes (439/4,338) were identified as differentially expressed (*p*-value ≤ 0.01) in both the PRO-seq (nascent transcription, 3 hours) and RNA-seq (steady-state mRNA, 20 hours) experiments in Nutlin-treated WTp53 cells (WTp53:WTp53 data shown in **S14C Fig**). This percentage is roughly consistent with previous studies that compared global run-on sequencing (GRO-seq) and RNA-seq data in Nutlin-treated cells [31] and provides a benchmark for the efficiency of mRNA biogenesis during a Nutlin-induced p53 response. Although the number of differentially expressed nascent transcripts (*n* = 315) was reduced in Nutlin-treated Δ40p53:WTp53 cells at 3 hours (**Fig 3C**), over 30 genes would be expected to be induced at the mRNA level, but this was not observed. These results suggest that mRNA biogenesis is defective for transcripts induced by Δ40p53:WTp53. Moreover, the mRNA data show striking parallels with bidirectional eRNA transcription. Like the mRNA transcriptome, changes in p53-dependent eRNA transcription were largely absent in Δ40p53:WTp53 cells.

### The p53 paralogs p63 or p73 do not impact Δ40p53:WTp53 response

Analysis of PRO-seq and RNA-seq data revealed that whereas p63 was expressed in MCF10A cells, p73 was not (**S15 Fig**). Stable knockdown of p63 (**S16A Fig**) in each cell line (WTp53, WTp53:WTp53, or Δ40p53:WTp53) did not impact proliferation or the cell cycle compared with controls (**S16B and S16C Fig**); moreover, p63 was not induced by Nutlin-3a in any cell line (**S15 Fig**). Finally, reverse transcription quantitative PCR (RT-qPCR) experiments showed that loss of p63 did not significantly impact CDKN1A/p21 or PUMA gene induction in Nutlin-treated WTp53, WTp53:WTp53, or Δ40p53:WTp53 cells (**S16D Fig**). Collectively, these results suggest that p63 and p73 do not contribute to the differential phenotypic or transcriptional responses in Δ40p53:WTp53 cells.

### WTp53 and Δ40p53:WTp53 support similar cellular responses to 5-fluorouracil via distinct TFs

**Fig 4:**
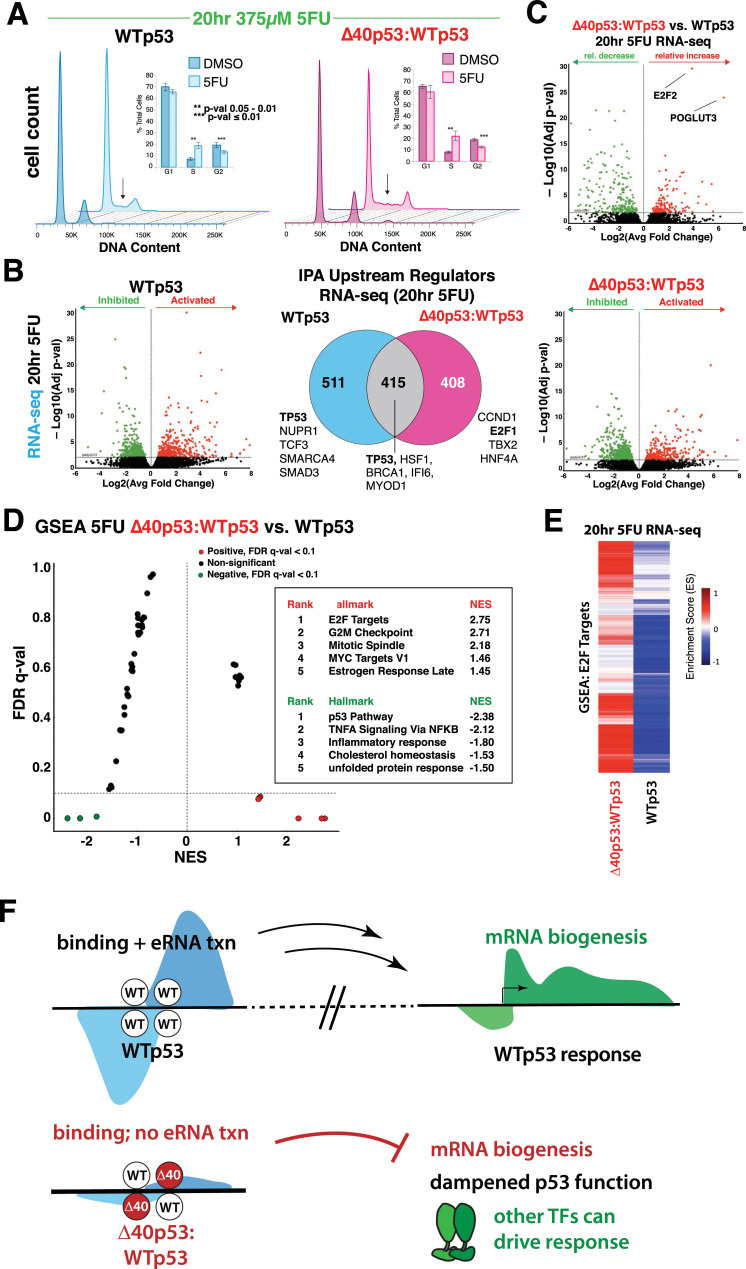
Cellular response to 5FU similar in WTp53 vs. Δ40p53:WTp53 cells, but driven by distinct TFs. **(A)** Cell cycle analysis (PI); chart (inset) represents the average of 3 experiments (bars = SEM). Arrows highlights increased S phase in both WTp53 and Δ40p53:WTp53 cells after 5FU treatment. **(B)** Volcano plots showing differentially expressed mRNAs after 20-hour 5FU treatment (vs. DMSO controls) in WTp53 or Δ40p53:WTp53 cells. Green dots represent down-regulated and red dots up-regulated transcripts (*p*-val ≤ 0.01). Venn diagram shows the overlap among IPA upstream regulators in 5FU-treated WTp53 vs. Δ40p53:WTp53 cells. For each subset of genes (WTp53-specific, shared, or Δ40p53:WTp53-specific), the top IPA transcription regulators associated with those genes are listed (minimum Z-score cutoff 2.0). **(C)** Volcano plot showing relative mRNA differences in 5FU-treated Δ40p53:WTp53 vs. WTp53 cells. **(D)** Mustache plot showing GSEA based upon RNA-seq data (20-hour 5FU treatment), comparing Δ40p53:WTp53 vs. WTp53. Consistent with **Figs 2B** and **3B**. Pathways with FDR q-val <0.1 are colored dots. Red represents increased in Δ40p53:WTp53 compared to WTp53, and green is decreased. X-axis is NES. Top significant pathways are shown in ranked list. **(E)** RNA-seq data (GSEA) shows that differential E2F2 TF activity characterizes the Δ40p53:WTp53 transcriptional response to 5FU. **(F)** Model. WTp53 induces eRNA transcription and drives cellular stress responses, whereas Δ40p53:WTp53 tetramers enable a tunable p53 response that allows other signal specific TFs to govern transcriptional outcomes. Despite Nutlin-dependent activation of p53 target gene nascent transcription (PRO-seq, 3 hours) in Δ40p53:WTp53 cells, subsequent increases in mRNA levels (RNA-seq, 20 hours) were not observed; moreover, p53-dependent induction of eRNAs was absent in Δ40p53:WTp53 cells. This disconnect between nascent transcription of p53 target genes and mRNA induction implicates eRNA transcription as a regulator of mRNA biogenesis. Underlying FACS data in the BioStudies database under accession number S-BSST672. Raw data for panels A–E in **S4 Data**. 5FU, 5-fluorouracil; eRNA, enhancer RNA; FACS, fluorescence-activated cell sorting; FDA, false discovery rate; GSEA, gene set enrichment analysis; IPA, ingenuity pathway analysis; NES, normalized enrichment score; PI, propidium iodide; PRO-seq, precision nuclear run-on sequencing; RNA-seq, RNA sequencing; TF, transcription factor; TNF, tumor necrosis factor; WTp53, wild-type p53.

Finally, we asked whether a different p53 stimulus would cause the same transcriptional defects in Δ40p53:WTp53 cells, i.e., a lack of response at the mRNA level. Because Nutlin-3a is highly selective for p53 (i.e., other TFs are not affected), it provided an efficient means to interrogate p53-specific transcriptional effects. However, Nutlin-3a is not physiologically relevant, and typical stress responses activate multiple pathways and their respective signal-specific TFs. We therefore selected 5-fluorouracil (5FU), a well-studied, clinically relevant chemotherapeutic that not only activates the p53 pathway but also other signaling cascades associated with DNA damage [44]. WTp53, WTp53:WTp53, and Δ40p53:WTp53 cells were treated with 375 μM 5FU for 20 hours. Cell cycle analysis showed that 5FU caused similar changes in WTp53 versus Δ40p53:WTp53 cells (**Fig 4A**), with a significant increase in S phase and a significant decrease in G2 phase. As expected, WTp53 and WTp53:WTp53 cells also showed similar results upon treatment with 5FU (**S17A Fig**). These results were not entirely surprising, since each cell line (WTp53, WTp53:WTp53, and Δ40p53:WTp53) was isogenic apart from the TP53 locus; thus, all auxiliary pathways were expected to respond similarly.

To further compare and contrast the cellular responses to 5FU, we conducted biological replicate RNA-seq experiments. As shown in **Fig 4B**, 5FU caused differential expression of 926 genes in WTp53 cells and 823 genes in Δ40p53:WTp53 cells (*p*-value ≤ 0.01; WTp53:WTp53 data shown in **S17B Fig**). Of these, 415 were shared among WTp53 and Δ40p53:WTp53, and many of these genes were p53 targets (**Fig 4B**), as revealed by IPA. About half (408 out of 823) of the differentially expressed genes in Δ40p53:WTp53 cells were regulated by other TFs such as E2F1 (**Fig 4B**). This result indicated that, in contrast to the targeted, p53-specific Nutlin response, the complex stress response induced by 5FU enabled other signal-specific TFs to augment the attenuated p53 response in Δ40p53:WTp53 cells.

As shown in **Fig 4C**, mRNA levels of the TF E2F2 were selectively up-regulated in Δ40p53:WTp53 cells, suggesting that E2F2 helps compensate for the reduced p53 response in cells expressing Δ40p53:WTp53. In WTp53 cells, E2F2 mRNA levels decreased by 57% (versus DMSO, with a 10% decrease in WTp53:WTp53 cells) upon 5FU treatment. By contrast, E2F2 mRNA levels increased by 398% in Δ40p53:WTp53 cells (fold change of 14.4), and E2F2 pathway genes were selectively up-regulated in Δ40p53:WTp53 cells (**Fig 4D, E**;WTp53:WTp53 **S18A Fig**). Interestingly, the E2F family of TFs have overlapping functions with p53, but p53 activation typically down-regulates E2F gene expression [37]. E2F2 induction in 5FU-treated Δ40p53:WTp53 cells suggested that, instead of p53, E2F and other TFs drive the transcriptional response toward cellular outcomes similar to WTp53. This concept was further supported by pathway analyses, which revealed that despite a different set of active and repressed TFs in Δ40p53:WTp53 cells versus WTp53 (**S18B Fig**), the upstream regulators identified by IPA were similar (**S18C Fig**). Consistent with these results, 5FU treatment caused similar cell cycle changes in WTp53 versus Δ40p53:WTp53 cells (**Fig 4A**).

To further probe p53-dependent effects on the 5FU response, and whether E2F and other TFs compensate for reduced p53 activity in Δ40p53:WTp53 cells, we completed cell cycle analyses in p53 null MCF10A cells following 20-hour treatment with 375 μM 5FU (conditions identical to prior experiments). The results showed that cellular responses were similar to WTp53 (**S18D Fig**), suggesting that, despite strong p53 activation by 5FU in WT cells, p53 is not required for cell cycle changes in this context. We next analyzed published RNA-seq data that compared 5FU-treated WTp53 and p53 null HCT116 cells [45]. Notably, GSEA identified similar pathways and TFs (e.g., E2F) activated by 5FU in p53 null cells (**S18E Fig**), compared with Δ40p53:WTp53 cells (**Fig 4D**), providing further support for a network of TFs that can compensate for p53 if its activity is reduced (e.g., in Δ40p53:WTp53 cells) or even lost (as in p53 null cells). Taken together, the results from 5FU-treated cells revealed that other signal-specific TFs can drive transcriptional programs under conditions with tempered p53 activity, such as in Δ40p53:WTp53 cells (**Fig 4F**).

## Discussion

The Δ40p53 isoform is naturally occurring, and its levels appear to increase during cell stress [20,24–27] or during specific developmental stages [46]. The relative levels of Δ40p53 versus WTp53 will undoubtedly dictate its impact on WTp53 function under these circumstances. Here, we focused on p53 tetramers with a defined 2:2 ratio (Δ40p53:WTp53). Because spontaneous TP53 mutations can yield a proliferative advantage in cultured cells [47], we chose the MCF10A cell line for this study because it is genetically stable (**S2 Fig**), unlike many cancer-derived cell lines. MCF10A cells also endogenously express WTp53, and we confirmed that WTp53 or WTp53:WTp53 cells were phenotypically similar to unedited MCF10A cells under normal or p53-stimulated conditions (**S3 Fig**). We also tested additional genome-edited cell clones to rule out potential effects from clonal expansion (**S19 Fig**). Whereas more cell lines could be tested with identical sets of experiments, others have shown that basic p53 transcriptional responses are similar across cell types [31,48], and p53 transcriptional response was the primary focus of this work.

Based upon the transcriptomics data, several themes have emerged about how Δ40p53 alters WTp53 function (**Fig 4F**). Expression of Δ40p53 (1) modulates the transcriptional activity of WTp53, such that Δ40p53:WTp53 tetramers suppress typical p53 transcriptional responses. Despite this, (2) Δ40p53:WTp53 tetramers retain the ability to activate p53 target genes, as seen most clearly in 5FU-treated cells (**Fig 4B**) or upon comparison with p53 null cells (**S9B–S9D Fig**). That is, Δ40p53:WTp53 tetramers do not functionally mimic p53 null conditions. This ability of Δ40p53 to dampen the p53 response 3) allows other sequence-specific, DNA-binding TFs to tune cellular stress responses, because they are not dominated by p53. For example, the gene expression signatures for the signal-specific TF E2F2 substantially contributed to the 5FU response in Δ40p53:WTp53 cells (**Fig 4C–4E**). In this way, Δ40p53 allows augmentation of the p53 response by other stress- or signal-responsive TFs. This functional versatility may be especially important in specific contexts, cell types, or developmental stages that require a modified p53 response [49,50], for instance, to allow cell cooperation [51] and proliferation during embryonic development [46] or wound healing [52].

Furthermore, (4) the continual, enforced expression of Δ40p53 in the context of WTp53 (i.e., Δ40p53 + WTp53) causes accelerated aging in mice [16]. Whereas the biology of aging is complex [53], we observed that numerous pathways implicated in organismal aging were impacted in predictable ways by Δ40p53:WTp53. For example, the mTOR and IGF signaling pathways were up-regulated in Δ40p53:WTp53 cells (versus WTp53; **S9F Fig**), and each pathway is broadly implicated in aging [54,55]. Concomitant activation of p53 and the mTOR pathway was also noted upon transfection of Δ40p53:WTp53 in p53 null H1299 cells [15]; notably, simultaneous activation of p53 and mTOR can trigger senescence [56,57]. Senescent cells accumulate during physiological aging [58], and, consistent with the aging phenotype, mice that co-express N-terminal p53 truncations with WTp53 exhibit early senescence-associated phenotypes [17].

### Δ40p53 tempers WTp53 function, enabling other TFs to drive cellular processes

Related to items (3) and (4) above, the contrast between Nutlin-3a and 5FU is especially informative. Because Nutlin-3a is exquisitely specific for p53 [30,31], it was a valuable tool to selectively interrogate the p53 response. However, Nutlin stimulation is not physiologically relevant and does not activate other signal-specific TFs (i.e., only p53). In contrast, 5FU induces a DNA damage response that activates multiple signaling cascades.

A clear distinction in mRNA biogenesis was observed in cells treated with 5FU (versus Nutlin). Hundreds of mRNAs were induced in Δ40p53:WTp53 cells after 20 hours treatment with 5FU, including many canonical p53 target genes (**Fig 4B**). These results suggest that functional coordination among other signal-specific TFs helps drive mRNA biogenesis in Δ40p53:WTp53 cells. Whereas 5FU-treated Δ40p53:WTp53 cells showed diminished p53 activation (versus WTp53 cells), E2F2 expression levels (and its downstream target genes; **Fig 4D and 4E**) increased significantly, revealing that other signal-responsive TFs augment cellular responses in the presence of Δ40p53:WTp53. Thus, the dampened p53 response in Δ40p53:WTp53 cells allows other TFs to drive the cellular response. These results are consistent with the concept of collaborative TF networks that enable compensatory responses if the function of typical “driver” TFs is compromised [59]. Such compensatory mechanisms appear to hard-wire cellular stress responses to ensure robust and consistent outcomes; this may be especially important in vivo, to coordinate transcriptional responses across organs and tissues. Notably, increased levels of Δ40p53 occur during endoplasmic reticulum (ER) stress [24,26], serum stimulation after starvation [20], and oxidative stress [25]. Multiple TFs and signaling cascades are activated under these circumstances, and the presence of Δ40p53:WTp53 tetramers may be important to allow integration of other pathway- and signal-specific TFs to modulate the cellular response in cell type–or context-specific ways.

### eRNA transcription and mRNA biogenesis

Many studies have linked eRNA transcription with mRNA expression [60], including in response to p53 activation [38,61]. A general theme has been that increased eRNA transcription correlates with increased expression of protein-coding genes; moreover, the timing of eRNA induction implies a regulatory role, as eRNAs are rapidly induced after a stimulus, followed by increased mRNA levels of protein-coding genes.

Here, we inadvertently established a system that, for the first time, allowed analysis of the transcriptional response to p53 activation in the presence or absence of p53-dependent eRNA induction, in largely isogenic cell lines. Our results implicate eRNA transcription as a prerequisite for mRNA biogenesis. In support of this conclusion, eRNA transcription at p53 binding sites was blocked in Δ40p53:WTp53 cells (e.g., **S12C Fig**)—despite similar genomic occupancy versus WTp53—and this tracked with mRNA levels. For instance, PRO-seq data showed that several hundred transcripts increased after 3-hour Nutlin treatment in Δ40p53:WTp53 cells (**Fig 2A**), but only 1 gene (0.3%), CDKN1A/p21 increased at the mRNA level (**Fig 3A**). By contrast, PRO-seq data from Nutlin-induced WTp53 cells showed an increase in 4,607 transcripts, with 439 increased at the mRNA level (approximately 10%; **Fig 3C**). These results establish a direct connection between eRNA transcription and mRNA biogenesis (**Fig 4F**). Indeed, the experiments described herein were (i) completed in virtually isogenic cell lines, with (ii) virtually the same TF (only lacking 2 of 4 AD1 domains for Δ40p53:WTp53), with (iii) the same genomic occupancy, and (iv) under the same stimulus. The mechanistic basis remains to be determined, but eRNA-dependent regulation of RNA processing (e.g., splicing, cleavage, and polyadenylation) or nuclear export could contribute.

### Four complete p53 activation domains are required for eRNA transcription

Because Δ40p53:WTp53 was expressed from the native TP53 locus, the timing and the levels of induction matched WTp53; moreover, ChIP-seq data indicated that occupancy of Δ40p53:WTp53 versus WTp53 on genomic DNA was similar in basal and Nutlin-induced conditions. The lack of p53-dependent eRNA induction by Δ40p53:WTp53 is therefore attributed to loss of only 2 of the 4 p53AD1 regions in the tetramer. That is, 2 activation domains are not sufficient for normal p53 tetramer function in cells. These findings suggest that p53 may have evolved to function as a tetramer (i.e., delivering 4 ADs to genomic DNA), at least in part, to induce eRNA transcription. Although additional experiments are needed to better define the molecular mechanisms that underlie this unexpected result, it is notable that p53 binding sites are more isolated in the human genome [62], whereas other TFs function within enhancers with clustered binding sites for many factors [63]. Potentially, 4 complete p53 activation domains (i.e., AD1 + AD2) are needed to stably recruit cofactors, such as Mediator, chromatin remodelers, and CBP/p300, to induce eRNA transcription. Alternately, 4 complete p53 activation domains might promote formation of molecular condensates that could help drive eRNA transcription by RNA polymerase II [64]. In support of this hypothesis, the activation domains of p53 is intrinsically disordered, and p53 has been shown to form phase separated condensates in vitro [65]. These mechanisms (cofactor recruitment or condensate formation) are not mutually exclusive and may act synergistically in cells.

### Implications for p53 tetramer structure

To date, no structural data exist for the entire p53 tetramer at atomic resolution, and it is evident that the p53 tetramer is structurally dynamic [66,67]. This presents challenges for structural analysis, even with cryo-EM. The flexibly tethered p53 tetramers described herein (WTp53:WTp53 or Δ40p53:WTp53) were designed based upon the cryo-EM structure of the native p53 tetramer at intermediate resolution [29]. As far as we are aware, this structure by Orlova and colleagues is the only instance in which wild-type, full-length p53 was used, and it remains the highest resolution p53 tetramer structure to date (13.7 Å). Whereas alternate structural models of the p53 tetramer have been proposed [68,69], these have used mutant versions of p53 (truncations or with 4 mutations in each core DNA-binding domain, thus 16 mutations per tetramer) that showed evidence for structural instability and heterogeneity and yielded lower resolution information.

Previous proof-of-concept biochemical and transcriptomics experiments demonstrated that the flexible tethers linking p53 monomers did not disrupt normal p53 tetramer function. For instance, WTp53:WTp53 tetramers matched gene expression changes induced by WTp53 in H1299 cells [15]. Similar results were obtained in genome-edited MCF10A cells here (e.g., **S8 Fig**), and ChIP-seq data showed similar genomic occupancy for WTp53 versus WTp53:WTp53 (**S13 Fig**). Likewise, MCF10A cells expressing WTp53 or WTp53:WTp53 were phenotypically indistinguishable under normal growth conditions or in response to Nutlin-3a or the genotoxic agent 5FU. These results best support the structural model of Orlova and colleagues [29,70]. Nevertheless, we emphasize that our flexible tether strategy could enable formation of the “alternate” p53 tetramer structures as well [68,69]. Because high-resolution data are lacking for WTp53 tetramers, its structural organization has remained controversial, and it remains plausible that full-length p53 may adopt multiple structural states that are functionally relevant.

### CDKN1A/p21 induction and Δ40p53:WTp53 biological functions

Despite the inability of Δ40p53:WTp53 tetramers to induce eRNA transcription or mRNA production in Nutlin-treated cells, an exception was CDKN1A (aka p21), which is a well-established p53 target gene. CDKN1A inhibits the cyclin-dependent kinases (CDKs) CDK2 and CDK4/6, which phosphorylate RB-related proteins to promote G1/S cell cycle arrest. Whereas Nutlin-dependent activation of p53 triggered cell cycle arrest in WTp53 cells, this was not observed with Δ40p53:WTp53 (**Fig 1D**). This phenotypic difference (p53 activation without cell cycle arrest) suggests Δ40p53 expression may be oncogenic; however, this was not observed in mouse models [16]. Notably, p21 is still induced in Nutlin-treated Δ40p53:WTp53 cells (although less compared with WTp53), at the protein and mRNA level (**Figs 1E and 3A**). This low-level p21 expression is likely an important aspect of Δ40p53:WTp53 function in biology. Loss of p21 results in polyploidy, due to defects in DNA damage response and cycling in the absence of mitosis [71]. Low-level p21 expression by Δ40p53:WTp53 may help maintain genomic stability by retaining the mitotic checkpoint to prevent polyploidy. As a naturally occurring isoform, this basic function may be essential while Δ40p53 regulates stress responses throughout the mammalian life span.

## Methods

### Cas-9 protein purification

Cas-9 purification was completed as described [72].

### Cas-9 RNP formation

Single-guide RNA (sgRNA) was formed by adding tracrRNA (IDT (Coralville, Iowa) cat# 1072533) and crRNA (TP53 exon 2, positive-strand, AGG PAM site, sequence: GATCCACTCACAGTTTCCAT) in a 1:1 ratio and heated to 95°C then slowly cooled to room temperature over 1 hour. Purified Cas-9 was added to sgRNA at a ratio of 1:1.2 and incubated at 37°C for 15 minutes, forming Cas-9 RNP. Cas-9 RNP was used at 10 μM concentration within an hour.

### Donor plasmid construction

VectorBuilder was used to construct the plasmid. The insert (see **S1A Fig**) was flanked by 1.5-kb homology arms, and mCherry was inserted as a selection marker. Insertion sizes were as follows: WTp53: 2820bp, WTp53:WTp53: 4041bp, and Δ40p53:WTp53: 3924bp.

### CRISPR/Cas-9 genome editing

MCF10A cells were split 24 hours prior to each experiment and grown to approximately 70% confluence on a 15-cm plate. Cells were washed with PBS, followed by trypsinization (4 ml per plate) and resuspended in resuspension media (8 mL DMEM/F12 containing 20% horse serum and 1x pen/strep). Moreover, 5 × 10^5^ cells were placed in 1.5-mL Eppendorf tubes for transfection with neon transfection system (Invitrogen, (Carlsbad, California) #MPK5000). Cells were resuspended in resuspension Buffer R (Invitrogen, #MPK1025) with 10 μM Cas-9 RNP and 1 μg donor plasmid (WTp53, WTp53:WTp53, or Δ40p53:WTp53). Furthermore, 10 μL Neon pipet tip (Invitrogen, #MPK1025) electroporated using the Neon Transfection Kit (1,400 V, 20-ms width, 2 pulses). Transfected cells were transferred to 2-mL antibiotic free media. Cut location: hg38 chr17:7,676,510. Insertion location: hg38 chr17:7,676,591; after the first ATG for TP53 in exon 2. Cells were single cell sorted into 96-well plates based on mCherry expression. Clones were then verified with sequencing, PCR, and western blot.

### MCF10A cell culture

MCF10a cells cultured in DMEM/F12 (Invitrogen #11330–032) media containing 5% horse serum (LifeTech (Rochester, New York) #16050–122), 20 ng/mL epidermal growth factor (EGF; PeproTech (Minneapolis, Minnesota) #AF-100-15), 0.5 ug/mL Hydrocortisone (Sigma (St. Louis, Missouri) #H0888-1g), 100 ng/mL Cholera toxin (Sigma #C8052-2mg), 10 ug/mL insulin (Sigma #I1882-200mg), and 1x Gibco 100x Antibiotic-Antimycotic (Fisher Scientific, (Carlsbad, California) 15240062) penicillin-streptomycin.

### PCR verification of insertions at TP53 locus

NEB Phusion polymerase (NEB, (Ipswitch, Massachusetts) #M0530S) was used to the manufacturer’s specifications. Seq_TP53_exon2_Forward and Seq_TP53_exon2_Reverse primers were used at 65 to 68°C; homozygous knock-in clones had no 500-bp product. Product was sequenced using TP53exon2-Forward primer. Using DBD_Forward and Seq_TP53_exon2_Reverse primers at 67.8°C, a 2,542-bp band indicated knock-in for at least 1 allele (either homozygous or heterozygous clone). A homozygous clone was then verified if Seq_TP53_exon2_Forward and Seq_TP53_exon2_Reverse primers at 65 to 68°C had no 500-bp product.

Because mutations in p53 can yield a survival advantage [47], we periodically re-sequenced the TP53 locus. Primers used were CL802 and CL803 at 65°C, and DBD_Forward and DBD_Reverse were used for sequencing. For sequencing downstream of insert, we used DBD_Forward and Seq_TP53_exon2_Reverse at 70°C. For amplifying upstream of insert, we used CRISPR3 seq primer and CL803 at 70°C, and DBD_Reverse and Seq_TP53_exon2_Forward primers were used for sequencing. Primer sequences are listed in **S3 Table**.

### Compound treatments

Nutlin-3a (Selleck, (Houston, Texas) #S8059) stock concentration 10 mM, and treatment concentration 10μM. 5FU (Selleck, #S1209) stock concentration 100 mM, and treatment concentration 375 μM. Equivalent volumes of DMSO vehicle were used for controls.

### Metabolomics

Cells were harvested after 20-hour treatment with Nutlin-3a (10 μM) or 0.1% DMSO controls, with 6 biological replicates for each cell line and each condition. Sample preparation was carried out at Metabolon (Durham, North Carolina, USA), in a manner similar to a previous study [73]. Briefly, individual samples were subjected to methanol extraction then split into aliquots for analysis by ultrahigh-performance liquid chromatography/mass spectrometry (UHPLC/MS). The global biochemical profiling analysis comprised 4 unique arms consisting of reverse phase chromatography, positive ionization methods optimized for hydrophilic compounds (LC/MS Pos Polar) and hydrophobic compounds (LC/MS Pos Lipid), reverse phase chromatography with negative ionization conditions (LC/MS Neg), as well as a HILIC chromatography method coupled to negative ionization (LC/MS Polar) [74]. All of the methods alternated between full scan MS and data dependent MS^*n*^ scans. The scan range varied slightly between methods but generally covered 70 to 1,000 *m*/*z*.

Metabolites were identified by automated comparison of the ion features in the experimental samples to a reference library of chemical standard entries that included retention time, molecular weight (m/z), preferred adducts, and in-source fragments, as well as associated MS spectra, and curated by visual inspection for quality control using software developed at Metabolon. Identification of known chemical entities was based on comparison to metabolomic library entries of purified standards [75]. A summary of all metabolomics data is shown in **S4 Table**.

### Statistical analysis of metabolomics data

Two types of statistical analyses were performed: (1) significance tests and (2) classification analysis. Standard statistical analyses were performed in Array Studio on log-transformed data. For analyses not standard in Array Studio, the R program (http://cran.r-project.org/) was used. Following log transformation and imputation of missing values, if any, with the minimum observed value for each compound, Welch 2-sample *t* test was used as a significance test to identify biochemicals that differed significantly (*p* < 0.05) between experimental groups. An estimate of the false discovery rate (*q*-value) was calculated to take into account the multiple comparisons that normally occur in metabolomic-based studies. Classification analyses included principal component analysis (PCA), hierarchical clustering, and random forest. For the scaled intensity graphics, each biochemical in original scale (raw area count) was rescaled to set the median across all samples and time points equal to 1.

### Cell cycle analysis

WTp53, WTp53:WTp53, or Δ40p53:WTp53 cells were treated with 10 μM Nutlin-3a or 375 μM 5FU for 20 hours in parallel with DMSO controls, 0.1% and 0.375%, respectively. Propidium Iodide (PI) Flow Cytometry Kit (Abcam, (Cambridge, Massachusetts) ab139418) was used as specified by the manufacturer. Samples were then placed on ice and analyzed with a BS FACSCelesta cell analyzer. FLOWJO was used to analyze fluorescence-activated cell sorting (FACS) data. All experiments were performed in biological triplicate.

### Immunoflourescence

WTp53, WTp53:WTp53, and Δ40p53:WTp53 were treated with 10 μM Nutlin-3a or 0.1% DMSO for 20 hours, collected, and fixed in 66% ethanol for at least 24 hours. Cells were then incubated with a blocking/permeabilization buffer (3% BSA, 0.1% Triton X-100) for 1 hour at room temperature. Primary antibody, anti-p21 (CST, (Danvers, Massachusetts) 2947) at 1:250 dilution, staining was carried out overnight at 4°C in the blocking buffer and visualized using secondary antibodies conjugated to Alexa Fluor 488.

### Growth rate calculations

To examine how growth rate change over time, cells were treated with 0.1% DMSO or 10 μM Nutlin-3a for 20 hours, passaged 1:10, and given 48 hours to recover (treatment cycle; **S5D Fig**). Growth rate was calculated by dividing the total cell growth by time of growth (68 hours). Total cell growth was calculated by counting with Nexcelom Bioscience Cellometter Auto T4 Bright Field Cell Counter (Nexcelom Bioscience, Lawrence, Massachusetts). All experiments were performed in biological triplicate.

### p63 lentiviral knockdown

WTp53, WTp53:WTp53, and Δ40p53:WTp53 cells were transduced with viral construct targeting p63 (shRNA TRCN0000006506; obtained from the University of Colorado Functional Genomics Core) under a constitutive hU6 promoter with puromycin resistance. Knockdown cells were selected with 48-hour treatment of 2 μg/mL puromycin followed by recovery in standard growth media.

### RT-qPCR

WTp53, WTp53:WTp53, Δ40p53:WTp53 cells ± p63 knockdown or alternate p53 cell clones were treated with 10 μM Nutlin-3a or DMSO control for 20 hours before RNA isolation using TRizol (Invitrogen, #15596026) as specified by the manufacturer. Total RNA was converted to cDNA using High-Capacity cDNA Reverse Transcription Kit (Applied Biosystems, (Foster City, California) 4368813) as specified by the manufacturer, followed by a cleanup performed using 0.8X AMPure XP beads (Beckman Coulter, (Indianapolis, Indiana) #A63881). Sybr Select Master Mix (Thermo Fisher Scientific, (Waltham, Massachusetts) 4472908) was added to cDNA at 0.1 ng/μL and amplified per manufacturer instructions. ΔΔCT values were background normalized using a gene desert. PCR primers are listed in **S3 Table**.

### RNA-seq

Total RNA was isolated from WTp53, WTp53:WTp53, and Δ40p53:WTp53 cells treated with 10 μM Nutlin-3a, 375μM 5FU, or DMSO controls (0.1% and 0.375%, respectively) using TRizol (Invitrogen, #15596026) as specified by the manufacturer. Moreover, 1 μg of total RNA with a RIN number of ≥8 was used for RNA-seq library prep. Sample was enriched for mRNA using NEBNext Poly(A) mRNA Magnetic Isolation Module (E7490), and library was prepared using NEBNext Ultra II Directional RNA Library Prep Kit from Illumina (E7765).

### ChIP-seq

MCF10A cell lines (WTp53, WTp53:WTp53, and Δ40p53:WTp53; approximately 60 × 10^6^ cells per experiment) were treated with 10 μM Nutlin-3a or 0.1% DMSO for 6 hours then cross-linked with 1% formaldehyde for 10 minutes at 25°C followed by glycine (0.125 M) quenching for 5 minutes. Nuclei were isolated by resuspending cells in NRO buffer (80 μl/million cells; 10 mM Tris-HCl [pH 8], 4 mM MgCl_2,_ 10 mM NaCl, 0.5% [vol/vol] NP-40, 1 mM DTT, and the protease inhibitor cocktail (1 mM Benzamidine (Sigma, #B6506-100G), 1 mM Sodium Metabisulfite (Sigma, #255556-100G), 0.25 mM Phenylmethylsulfonyl Fluoride (American Bioanalytical, (Natick, Massachusetts) #AB01620) 0.012 TIU/mL aprotinin (Sigma, #A6106), followed by a 5 minutes incubation on ice, a low speed spin, and then a final wash with NRO buffer. The isolated nuclei were prepared for shearing based on the Covaris truChIP Chromatin Shearing Kit (Covaris: (Woburn, Massachusetts) PN 520237) and sheared for 11 minutes with Covaris M220 Focused-ultrasonicator. A total of 25 μL of Protein G Dynabeads beads (Invitrogen, #10003D) was used per 100 μg chromatin (approximately 15 million nuclei). Beads were incubated in blocking solution (PBS, 0.5% BSA) then 6 μg of DO1 p53 antibody (BD Biosciences: (Franklin Lakes, New Jersey) BD554293) was conjugated to beads in blocking solution, nutating at 4°C for 4 hours. Conjugated beads were washed 1x block solution and 1x IP buffer (15 mM Tris-HCl [pH 8], 150 mM NaCl, 1 mM EDTA, 1% Triton X-100 and the protease inhibitor cocktail). Sheared chromatin was added (100 μg chromatin (approximately 15 million nuclei)) to conjugated beads in IP buffer and then nutating at 4°C for at least 12 hours. Bound chromatin was washed 3x with IP buffer, 3x with RIPA buffer (20 mM Tris-HCl [pH 8], 500 mM NaCl, 1 mM EDTA, 1% Triton X-100 and 0.1% SDS), 2x with LiCl buffer (20 mM Tris-HCl [pH 8], 500 mM LiCl, 1 mM EDTA, 1% sodium deoxycholate and 1% NP-40) and 2x with TE Salt buffer (10 mM Tris-HCl [pH 8], 50 mM NaCl, 1 mM EDTA). Sample was eluted from beads with PK buffer (10 mM Tris-HCl [pH 8], 1 mM EDTA and 1% SDS) containing 100 μg of Proteinase K (NEB: P81075) incubated at 50°C, with shaking, for 1 hour then at 65°C for 1 hour. Eluted DNA was transferred to a new tube and incubated at 65°C for 12 hours to reverse cross-linking. DNA was purified by phenol chloroform extraction using Light-5PRIME Phase Lock Tubes (Quantabio (Beverly, Massachusetts): 2302820) based on the manufacturer’s instructions. Libraries were prepared using the KAPA Hyper Prep Kit (Roche, Wilmington, Massachusetts: KK8502) and sequenced on Illumina NextSeq V2 high output 75-cycle.

### PRO-seq nuclei preparation, nuclear run-on, and RNA preparation

Nuclei were harvested for WTp53, WTp53:WTp53, Δ40p53:WTp53, or p53 null MCF10A cells, as described [76]. The cells were grown for 24 hours prior to the harvest, to 70% confluency. At this point, cells were treated simultaneously with 10 μM Nutlin-3a (in DMSO) or 0.1% DMSO for 3 hours before being harvested. The nuclear run-on experiments were performed with biological duplicates as described [76].

### Sequencing

Sequencing of RNA-seq, ChIP-seq, and PRO-Seq libraries was performed at the BioFrontiers Sequencing Facility (UC Boulder). Single-end fragment libraries (75 bp) were sequenced on the Illumina NextSeq 500 platform (RTA version: 2.4.11, Instrument ID: NB501447), demultiplexed and converted BCL to fastq format using bcl2fastq (bcl2fastq v2.20.0.422); sequencing data quality was assessed using FASTQC (v0.11.5) (https://www.bioinformatics.babraham.ac.uk/projects/fastqc/) and FastQ Screen (v0.11.0, https://www.bioinformatics.babraham.ac.uk/projects/fastq_screen/). Trimming and filtering of low-quality reads was performed using BBDUK from BBTools (v37.99) (https://www.osti.gov/servlets/purl/1241166) and FASTQ-MCF from EAUtils (v1.05) [77]. Alignment to the human reference genome (hg38) was carried out using Hisat2 (v2.1.0) [78] in unpaired, no-spliced-alignment mode with an hg38 index, and alignments were sorted and filtered for mapping quality (MAPQ>10) using Samtools (v1.5) [79].

### RNA-seq computational analysis

The RNA-seq data were processed using a Nextflow pipeline v1.1 (https://github.com/Dowell-Lab/RNAseq-Flow). A full pipeline report of the run as well as a quality control report generated by MultiQC (v. 1.7), including trimming, mapping, coverage, splicing, and complexity metrics are included in **S5 Table**. Gene counts were generated using featureCounts [80] and differential gene expression analysis was performed using DESeq2 [35]. Duplicate genes were filtered and those with the highest FPKM were kept for analysis. Qiagen ingenuity pathway analysis (IPA) and GSEA 4.03 [81] were used for identification of activated and inhibited pathways and upstream regulators based on expression changes.

### ChIP-seq computational analysis

All ChIP-seq data were processed (mapped and quality checked) using a Nextflow pipeline, ChIP-Flow v1.0 (https://github.com/Dowell-Lab/ChIP-Flow). A full pipeline report of the run as well as a quality control report generated by MultiQC (v. 1.7), including trimming, mapping, coverage, and complexity metrics are included in **S6 Table**. Peak calls were generated using MACS2 narrowPeak. The q-value default cutoff was also decreased from the default of 0.05 to 1e-5. Blacklisted regions (those having artificially high signal and read mapping, http://mitra.stanford.edu/kundaje/akundaje/release/blacklists/hg38-human) were removed using BEDTools intersect [82]. PyGenomeTracks [83] was used to generate track images.

### Data processing, visualization, and identification of eRNAs

Two biological replicates for each treatment (DMSO/Nutlin-3a) were generated for each of the 3 cell lines were processed using a Nextflow pipeline for nascent data (https://doi.org/10.5281/zenodo.2641755). A full pipeline report of the run as well as a quality control report generated by MultiQC (v. 1.7), including trimming, mapping, coverage, and complexity metrics, is included in **S7 Table**. PyGenomeTracks [83] was used to generate track images. Tfit was used to identify regions with bidirectional transcription [84]. Note that in Nutlin-treated Δ40p53:WTp53 cells, 86 eRNAs were identified with a *p*-value ≤ 0.01; after removal of all repetitive regions, 25 eRNAs remained. TFEA was used to identify changes in bidirectional transcription and map to underlying TF sequence motifs to infer changes in TF activity [40].

### Differential transcription analysis of genes and bidirectionals/enhancers (PRO-seq)

Using the RefSeq: NCBI Reference Sequences for hg38, including both NM and NR accession types (downloaded from the UCSC track browser on May 18, 2018), counts were calculated for each sorted BAM file using multiBamCov in the BEDTools suite (v. 2.25.0). Genes (NM accession type) and lncRNAs (NR accession type) were then filtered such that only the isoform with the highest number of reads per annotated length was kept in order to minimize duplicate samples being included in differential transcription analysis. DESeq2 (v. 1.20.0, Bioconductor release v. 3.7) was then used to determine differentially transcribed genes between the different treatments both within and between time points. A prerank file was generated using the results from the differential analysis from each pairwise comparison and used in differential pathway analysis using GSEA 4.0.3, using hallmark pathways gene sets. Qiagen IPA (v7.2) was used for identification of activated and inhibited pathways based on transcriptional changes. For bidirectional/enhancer comparisons, all bidirectional prediction Tfit calls were merged using mumerge software (merge component of TFEA) to generate an annotation file. Counts were then calculated for each sample using multicov from the BEDTools suite (v. 2.28.0) [82], and DESeq2 [35] was used to calculate differentially transcribed bidirectionals. TFEA was used to assess p53 activation based on eRNA expression [40].

### Additional cell line validation

Cell lines were internally validated by mapping PRO-seq reads to the p53 construct as a mini “genome” using Hisat2 (v2.1.0) [78], and alignments were sorted using Samtools (v1.5) [79]. Counts were then calculated for each sample using multicov from the BEDTools suite (v. 2.28.0) [82] and regions were compared for their read density over TAD1 against to the central region (TAD2, DBD and carboxyl-terminal region) of p53.

### Quantification and statistical analysis

PRO-seq and RNA-seq experiments were completed in biological replicate. ChIP-seq experiments were completed with biological triplicates. Metabolomics experiments were completed with 6 biological replicates. Statistical analysis of sequencing data and metabolomics data is provided in Method details.

## Supporting information

S1 Fig Additional information about genome-edited cell lines. **(A)** Overview of donor repair plasmids co-transfected with Cas-9 RNPs. **(B)** Scheme for CRISPR/Cas-9 insertions at the native TP53 locus: WTp53 (2820bp), WTp53:WTp53 (4041bp), and Δ40p53:WTp53 (3924bp). **(C)** PCR validation. Blue primer sets span the insertion and red primer sets have forward primer within the insertion and the reverse primer outside the insertion. Lack of 500-bp band with blue primers indicated homozygous insertion. PCR experiments were completed on genomic DNA isolated from each of the indicated cell lines. Non-edited MCF10A cells were tested as an additional control. **(D)** Western blot validation of each genome-edited cell line, using a p53 antibody (DO-1). As designed, each tethered construct (Δ40p53:WTp53 or WTp53:WTp53) migrated at around 100kDa, indicative of dimer expression as a single transcript. The flexible tether for Δ40p53:WTp53 or WTp53:WTp53 contained a TEV cleavage site, and treatment with TEV protease (+, as shown) cleaved the dimers to p53 monomers, as expected. Note that the Δ40p53 monomer lacks the epitope detected by the p53 DO-1 antibody. See **S1 Raw Images** for uncropped gels. CTR, carboxyl-terminal region; IRES, internal ribosomal entry site; PolyA, SV40 polyA sequence with terminator to prevent downstream transcription; TA1, transactivation domain 1; TA2, transactivation domain 2; WTp53, wild-type p53.
(EPS)

S2 Fig Additional validation of CRISPR/Cas-9 knock-in cell lines. **(A)** Sequencing results from the p53 DNA-binding domain show no mutations at common hotspots in any of the cell lines. Because p53 mutations can yield proliferative advantages in culture [47], DNA-binding domain sequencing was performed several times throughout the project to ensure no mutations occurred during the course of our experiments. **(B)** Internal validation of the edited p53 cell lines, using PRO-seq data. The PRO-seq data were mapped to the inserted p53 sequences at the native TP53 locus. RNA sequence corresponding to the first 39 amino acids of p53 was reduced by half in the Δ40p53:WTp53 cell line vs. WTp53, as expected. This method also allowed verification of p53 copy number at 2 per cell line. PRO-seq, precision nuclear run-on sequencing; WTp53, wild-type p53.
(EPS)

S3 Fig Endogenous MCF10A cells phenotypically match CRISPR/Cas-9 edited WTp53 and WTp53:WTp53 cell lines. Unedited “off-the-shelf” MCF10A cells, edited WTp53, and WTp53:WTp53 cells each display the same cell cycle phenotypes. **(A)** Cell cycle analysis (PI) and bar plots **(B)** represent the average of 6 biological replicates (bars = standard error of mean). Note that MCF10A cells endogenously express WTp53. Underlying FACS data in the BioStudies database under accession number S-BSST672. Raw data for panel B in **S5 Data.** FACS, fluorescence-activated cell sorting; PI, propidium iodide; WTp53, wild-type p53.
(EPS)

S4 Fig WTp53 vs. Δ40p53:WTp53 phenotypic and metabolic similarity under normal growth conditions but differences upon p53 activation with Nutlin-3a. **(A)** Cell cycle analysis (PI) of each genome-edited cell line. Chart (inset) represents the average of 6 experiments (bars = standard error of mean). **(B)** Sample of metabolomics data comparing IPA identified cell cycle metabolites under normal growth conditions in each cell line (6 biological replicates each). Log2 (fold change) was normalized to WTp53. Underlying FACS data in the BioStudies database under accession number S-BSST672. Raw data for panels A and B in **S6 Data**. FACS, fluorescence-activated cell sorting; IPA, ingenuity pathway analysis; PI, propidium iodide; WTp53, wild-type p53.
(EPS)

S5 Fig WTp53:WTp53 cells are metabolically and phenotypically similar to WTp53 cells. **(A)** Cell cycle analysis (PI) in WTp53:WTp53 cells; chart (inset) represents the average of 6 biological replicate experiments (bars = standard error of mean). Arrow highlights the loss of S phase cells, similar to WTp53 cells (and in contrast with Δ40p53:WTp53 cells). **(B)** Measurement of p21 protein levels by FACS; similar to WTp53, p21 protein levels increase in WTp53:WTp53 cells after Nutlin-3a treatment. **(C)** Cell cycle analysis (PI) in p53 null MCF10a cells; chart (inset) represents the average of 3 biological replicate experiments (bars = standard error of mean). Bar plot shows time correlated p21 RT-qPCR (not statistically significant pval > 0.05). **(D)** Growth rate measured over 5 treatment cycles; each cycle encompassed Nutlin treatment for 20 hours, followed by splitting cells 1:10, then growth under normal conditions for 48 hours (3 biological replicates; bars = SEM). Note the similar growth characteristics of WTp53 vs. WTp53:WTp53 cells. Underlying FACS data in the BioStudies database under accession number S-BSST672. Raw data for panels A, C, and D in **S7 Data.** FACS, fluorescence-activated cell sorting; PI, propidium iodide; RT-qPCR, reverse transcription quantitative PCR; WTp53, wild-type p53.
(EPS)

S6 Fig Metabolomics show increased sphingolipid metabolites in Δ40p53:WTp53 cells. Metabolic changes in Δ40p53:WTp53 cells are similar vs. WTp53:WTp53 cells and vs. WTp53 cells after Nutlin-3a treatment for 20 hours. WTp53, wild-type p53.
(EPS)

S7 Fig Justification for PRO-seq analysis after 3-hour Nutlin-3a in MCF10A cells. RT-qPCR of nuclear RNA levels for CDKN1A/p21 in either HCT116 or MCF10A cells at 1 hour and 3 hours. These data show a delayed p53 response in MCF10A cells vs. HCT116, with the 3-hour induction roughly matching the 1-hour p21 nuclear RNA levels from HCT116 cells (arrows). Past GRO-seq results in 1 hour Nutlin-treated HCT116 cells [36] were used to guide the 3-hour time point chosen for MCF10A. Raw data in **S8 Data**. GRO-seq, global run-on sequencing; PRO-seq, precision nuclear run-on sequencing; RT-qPCR, reverse transcription quantitative PCR.
(EPS)

S8 Fig Nutlin induces similar transcriptional changes in WTp53:WTp53 and WTp53 cells. Summary of PRO-seq data for annotated genes differentially transcribed in WTp53 (y-axis) vs. WTp53:WTp53 cells (x-axis). Dashed line represents *p*-value 0.01. Venn diagram shows overlap between WTp53 and WTp53:WTp53 cells. Raw data in **S9 Data**. PRO-seq, precision nuclear run-on sequencing; WTp53, wild-type p53.
(EPS)

S9 Fig Differential transcription in Δ40p53:WTp53 cells; Δ40p53:WTp53 activates p53 response in contrast to p53 null cells. **(A)** List of genes that are selectively transcribed in Δ40p53:WTp53 vs. WTp53 cells (PRO-seq data, 3 hours Nutlin). **(B, C)** Volcano plots that show differentially expressed transcripts (PRO-seq) at gene bodies after 3-hour Nutlin-3a treatment (vs. DMSO controls) in **(B)** p53 null or **(C)** Δ40p53:WTp53 MCF10A cells. Green dots represent down-regulated and red dots up-regulated transcripts (*p*-val ≤ 0.01). **(D)** Ward cluster maps of enrichment of canonical p53 target genes in either Δ40p53:WTp53 (left) or p53 null MCF10A cells (right) after 3-hour Nutlin-3a treatment (PRO-seq). **(E)** Representative PRO-seq data from Nutlin-treated WTp53 or Δ40p53:WTp53 cells, and DMSO controls. Although Nutlin induction is observed in each cell line, activation is reduced in Δ40p53:WTp53 cells. **(F)** IPA based upon PRO-seq data (3-hour Nutlin treatment). Pathways highlighted in **bold** font have been linked to progeroid phenotypes in mice expressing Δ40p53 + WTp53 [16,17]. Raw data for panels B–D and F in **S10 Data**. IPA, ingenuity pathway analysis; PRO-seq, precision nuclear run-on sequencing; WTp53, wild-type p53.
(EPS)

S10 Fig Nutlin induces similar transcriptional changes (eRNA) in WTp53:WTp53 and WTp53 cells. Summary of PRO-seq data of eRNA transcription for WTp53 (y-axis) vs. WTp53:WTp53 (x-axis). Dashed line represents *p*-value 0.01. Venn diagram shows overlap between WTp53 and WTp53:WTp53 cells. Raw data for panel C in **S11 Data**. eRNA, enhancer RNA; PRO-seq, precision nuclear run-on sequencing; WTp53, wild-type p53.
(EPS)

S11 Fig PRO-seq data from all 3 cell lines (3-hour Nutlin-treated) and DMSO control. Note the similar responses in WTp53 and WTp53:WTp53 cells, whereas Δ40p53:WTp53 cells lack the ability to induce bidirectional eRNA transcription. The p53 binding motif (*p*-value ≤ 1 × 10^−5^) is shown with a dashed line, and the peaks correspond directly with ChIP-seq peaks shown in **S13B Fig**. ChIP-seq, chromatin immunoprecipitation sequencing; eRNA, enhancer RNA; PRO-seq, precision nuclear run-on sequencing; WTp53, wild-type p53.
(EPS)

S12 Fig TFEA reveals differential TF activation in Nutlin-treated cells. **(A–C)** TFEA [40] from PRO-seq data (3 hours Nutlin-3a) in WTp53, WTp53:WTp53, and Δ40p53:WTp53 cells. Whereas p53 induction is robust in WTp53 and WTp53:WTp53 cells, this is not observed in Nutlin-treated Δ40p53:WTp53 cells (*p*-val ≤ 1 × 10^−6^). This reflects a lack of eRNA transcription at p53 binding sites in Δ40p53:WTp53 cells. Note that TP53 and TP63 have almost identical binding motifs. **(D)** TFEA [40] from PRO-seq data (3 hours Nutlin-3a) in WTp53 vs. Δ40p53:WTp53 cells, indicating reduced p53 (and p63) activity in Δ40p53:WTp53 cells (*p*-val ≤ 1 × 10^−6^). This reflects a lack of eRNA transcription at p53 binding sites in Δ40p53:WTp53 cells. Note that TP53 and TP63 have almost identical binding motifs. **(E)** Metagene analysis showing average eRNA peak height, genome-wide, at p53 responsive eRNAs (*p*-val < 0.25) in WTp53:WTp53 cells. Raw data for panels A–E in **S12 Data**. eRNA, enhancer RNA; PRO-seq, precision nuclear run-on sequencing; TFEA, transcription factor enrichment analysis; WTp53, wild-type p53.
(EPS)

S13 Fig Additional ChIP-seq data across all 3 cell lines. **(A)** A series of ChIP-seq experiments were completed 1 hour, 3 hours, or 6 hours after Nutlin-3a treatment to assess the time dependence of p53 occupancy changes. Metagene analyses are shown (average ChIP-seq signal) at p53 binding sites in Nutlin-3a-treated WTp53, WTp53:WTp53 or Δ40p53:WTp53 cells at 1 hour, 3 hours and 6 hours. To our knowledge, following Nutlin treatment, all published p53 ChIP-seq data sets used 6 hours or longer time points. **(B)** Examples of ChIP-seq data in Nutlin-treated (6 hours) cells, compared with DMSO controls. ChIP-seq peaks correspond directly with PRO-seq eRNA peaks shown in **S11 Fig**. **(C)** Metagene analyses (filtered for peaks containing p53 motif) showing average ChIP-seq signal, genome-wide, at p53 binding sites in DMSO control vs. Nutlin-treated WTp53:WTp53 cells. Raw data for panels A and C in **S13 Data**. ChIP-seq, chromatin immunoprecipitation sequencing; eRNA, enhancer RNA; PRO-seq, precision nuclear run-on sequencing; WTp53, wild-type p53.
(EPS)

S14 Fig Transcriptional changes in WTp53:WTp53 cells; IPA comparisons for Δ40p53:WTp53 and WTp53. **(A)** Volcano plot showing differentially expressed mRNAs after 20 hours Nutlin treatment (vs. DMSO controls) in WTp53:WTp53 cells. Green dots represent down-regulated and red dots up-regulated transcripts (*p*-val ≤ 0.01). **(B)** Venn diagrams showing overlap among significantly induced transcripts from PRO-seq data (3 hours Nutlin) and RNA-seq data (20 hours Nutlin) in WTp53:WTp53 cells. **(C)** IPA of canonical pathways from RNA-seq data after 20 hours Nutlin-3a treatment in Δ40p53:WTp53 vs. WTp53 cells flanked by heat maps of log2 (fold change) of genes from p53 on the left, and cell cycle chromosome replication pathway on the right. P53 and cell cycle chromosome replication represent the most significantly down-regulated or up-regulated pathways, respectively, in Δ40p53:WTp53 cells (vs. WTp53). Raw data for panels A and B in **S14 Data**. IPA, ingenuity pathway analysis; PRO-seq, precision nuclear run-on sequencing; RNA-seq, RNA sequencing; WTp53, wild-type p53.
(EPS)

S15 Fig PRO-seq and RNA-seq data at TP63 and TP73 loci in WTp53, WTp53:WTp53, and Δ40p53:WTp53 cells. Genome browser views of p63 and p73, showing that p63 is transcribed (PRO-seq) and expressed (RNA-seq) in the MCF10A cell lines listed at right. The p73 locus, by contrast, shows transcription of p73-AS1 (p73 antisense 1), a mature antisense transcript. Neither p63 nor p73 was induced by Nutlin-3a treatment. Because p73 was not transcribed in MCF10A cells, we focused on p63, but we note some transcription is observed from the adjacent TP73-AS1 locus. Top: DMSO and Nutlin-3a tracks for PRO-seq in WTp53, WTp53:WTp53, and Δ40p53:WTp53 cells; bottom: DMSO and Nutlin-3a tracks for RNA-seq in WTp53, WTp53:WTp53, and Δ40p53:WTp53 cells. PRO-seq, precision nuclear run-on sequencing; RNA-seq, RNA sequencing; WTp53, wild-type p53.
(EPS)

S16 Fig The p53 paralog p63 does not impact Δ40p53:WTp53 phenotype or function. **(A)** Western blot to probe p63 levels before or after lentiviral p63 knockdown. **(B)** Growth rate measured over 5 Nutlin treatment cycles; each cycle encompassed 20 hours under basal (0.1% DMSO) vs. Nutlin-treated conditions, splitting cells 1:10, then growth for another 48 hours. **(C)** Cell cycle analysis (PI) of each genome-edited cell line in either control or p63 knockdown. Left shows the cell cycle after 20 hours of 0.1% DMSO treatment (control), and right shows the cell cycle after 20 hours of Nutlin3a treatment. **(D)** Knockdown of p63 shows no impact on p53 target gene induction in WTp53, WTp53:WTp53, or Δ40p53:WTp53 cell lines. RT-qPCR data are shown for p21 and PUMA (2 biological replicates; bars = SEM). N.S. designates non-significant comparisons. Underlying FACS data in the BioStudies database under accession number S-BSST672. Raw data for panels B and D in **S15 Data**. FACS, fluorescence-activated cell sorting; PI, propidium iodide; RT-qPCR, reverse transcription quantitative PCR; WTp53, wild-type p53.
(EPS)

S17 Fig Additional data on cellular responses to 5FU. **(A)** Cell cycle analysis (PI); chart (inset) represents the average of 3 experiments (bars = standard error of mean). Arrow highlights increased S phase in 5FU-treated WTp53:WTp53 cells, similar to both WTp53 and Δ40p53:WTp53 cells (**Fig 4A**). **(B)** Volcano plot showing differentially expressed mRNAs after 20 hours 5FU treatment (vs. DMSO controls) in WTp53:WTp53 cells. Green dots represent down-regulated and red dots up-regulated transcripts (*p*-val ≤ 0.01). Underlying FACS data in the BioStudies database under accession number S-BSST672. Raw data for panels A and B in **S16 Data**. 5FU, 5-fluorouracil; FACS, fluorescence-activated cell sorting; PI, propidium iodide; WTp53, wild-type p53.
(EPS)

S18 Fig The E2F pathway is activated in 5FU-treated Δ40p53:WTp53 cells. **(A)** RNA-seq data (GSEA) shows that differential E2F2 TF activity characterizes the Δ40p53:WTp53 transcriptional response to 5FU in comparison to WTp53 and WTp53:WTp53. **(B)** IPA showing upstream regulatory TFs, inferred from RNA-seq data in 5FU-treated Δ40p53:WTp53 vs. WTp53 cells. **(C)** The general upstream regulators (IPA) controlling the cellular response to 5FU are similar (shared inside oval) between Δ40p53:WTp53 and WTp53 cells, with a few exceptions (*p* < 0.01). **(D)** Cell cycle analysis (PI); chart (inset) represents the average of 3 experiments (bars = standard error of mean). Arrow highlights increased S phase in 5FU-treated p53 null MCF10a cells. **(E)** GSEA based upon RNA-seq data from Yang and colleagues comparing p53 null HCT116 cells vs. WTp53 HCT116 cells after a 24-hour 5FU treatment. Pathways with FDR q-val < 0.1 are colored dots. Red represents increased in p53 null cells compared to WTp53 cells, and green is decreased. X-axis is NES. Top significant pathways are shown in the ranked list. Underlying FACS data in the BioStudies database under accession number S-BSST672. Raw data for panels A–E in **S17 Data**. 5FU, 5-fluorouracil; FACS, fluorescence-activated cell sorting; FDR, false discovery rate; GSEA, gene set enrichment analysis; IPA, ingenuity pathway analysis; NES, normalized enrichment score; PI, propidium iodide; RNA-seq, RNA sequencing; WTp53, wild-type p53.
(EPS)

S19 Fig Alternative genome-edited clones show similar results compared with original cell line clones selected. **(A)** PCR validation of additional clones. Blue primer sets span the insertion, and red primer sets have forward primer within the insertion and the reverse primer outside the insertion. Lack of 500-bp band with blue primers indicated homozygous insertion. PCR experiments were completed on genomic DNA isolated from each of the indicated cell lines. Non-edited MCF10A cells were tested as an additional control. **(B)** Western blot validation of each genome-edited cell line, using a p53 antibody (DO-1). As designed, each tethered construct (Δ40p53:WTp53 or WTp53:WTp53) migrated at around 100 kDa, indicative of dimer expression as a single transcript. **(C)** Differences between the different WTp53, WTp53:WTp53, or Δ40p53:WTp53 clones were minimal as shown by the relative induction (RT-qPCR) for p21 (2 biological replicates; bars = SEM). **(D)** Cell cycle analysis (PI) of the alternative clone for each genome-edited cell line. Cell cycle was performed at 20 hours of 0.1% DMSO treatment (control) or 20 hours of Nutlin-3a (treatment). Underlying FACS data in the BioStudies database under accession number S-BSST672. Raw data for panels C and D in **S18 Data**. See **S2 Raw Images** for uncropped gels. FACS, fluorescence-activated cell sorting; PI, propidium iodide; RT-qPCR, reverse transcription quantitative PCR; WTp53, wild-type p53.
(EPS)

S1 Table Comparative metabolomics data under basal conditions. (PDF)

S2 Table Comparative metabolomics data with Nutlin stimulation. (PDF)

S3 Table Primer sequences for PCR. (PDF)

S4 Table Metabolomics data. (PDF)

S5 Table RNA-seq sample information. RNA-seq, RNA sequencing.
(PDF)

S6 Table ChIP-seq sample information. ChIP-seq, chromatin immunoprecipitation sequencing.
(PDF)

S7 Table PRO-seq sample information. PRO-seq, precision nuclear run-on sequencing.
(PDF)

S1 Data Numerical raw data. Associated with **Fig 1**. File contains tabs with labels corresponding to relevant panel.
(XLSX)

S2 Data Numerical raw data. Associated with **Fig 2**. File contains tabs with labels corresponding to relevant panel.
(XLSX)

S3 Data Numerical raw data. Associated with **Fig 3**. File contains tabs with labels corresponding to relevant panel.
(XLSX)

S4 Data Numerical raw data. Associated with **Fig 4**. File contains tabs with labels corresponding to relevant panel.
(XLSX)

S5 Data Numerical raw data. Associated with **S3 Fig**. File contains tabs with labels corresponding to relevant panel.
(XLSX)

S6 Data Numerical raw data. Associated with **S4 Fig**. File contains tabs with labels corresponding to relevant panel.
(XLSX)

S7 Data Numerical raw data. Associated with **S5 Fig**. File contains tabs with labels corresponding to relevant panel.
(XLSX)

S8 Data Numerical raw data. Associated with **S7 Fig**. File contains tabs with labels corresponding to relevant panel.
(XLSX)

S9 Data Numerical raw data. Associated with **S8 Fig**. File contains tabs with labels corresponding to relevant panel.
(XLSX)

S10 Data Numerical raw data. Associated with **S9 Fig**. File contains tabs with labels corresponding to relevant panel.
(XLSX)

S11 Data Numerical raw data. Associated with **S10 Fig**. File contains tabs with labels corresponding to relevant panel.
(XLSX)

S12 Data Numerical raw data. Associated with **S12 Fig**. File contains tabs with labels corresponding to relevant panel.
(XLSX)

S13 Data Numerical raw data. Associated with **S13 Fig**. File contains tabs with labels corresponding to relevant panel.
(XLSX)

S14 Data Numerical raw data. Associated with **S14 Fig**. File contains tabs with labels corresponding to relevant panel.
(XLSX)

S15 Data Numerical raw data. Associated with **S16 Fig**. File contains tabs with labels corresponding to relevant panel.
(XLSX)

S16 Data Numerical raw data. Associated with **S17 Fig**. File contains tabs with labels corresponding to relevant panel.
(XLSX)

S17 Data Numerical raw data. Associated with **S18 Fig**. File contains tabs with labels corresponding to relevant panel.
(XLSX)

S18 Data Numerical raw data. Associated with **S19 Fig**. File contains tabs with labels corresponding to relevant panel.
(XLSX)

S1 Raw Images Uncropped agarose gels and western blots associated with S1 Fig. (EPS)

S2 Raw Images Uncropped agarose gels and western blots associated with S19 Fig. (EPS)

## Abbreviations

5FU: 5-fluorouracil
CDK: cyclin-dependent kinase
ChIP-seq: chromatin immunoprecipitation sequencing
cryo-EM: cryo-electron microscopy
EGF: epidermal growth factor
ER: endoplasmic reticulum
eRNA: enhancer RNA
FACS: fluorescence-activated cell sorting
GRO-seq: global run-on sequencing
GSEA: gene set enrichment analysis
IGF-1: insulin-like growth factor 1
IPA: ingenuity pathway analysis
IRES: internal ribosomal entry site
mTOR: mammalian target of rapamycin
PCA: principal component analysis
PI: propidium iodide
PRO-seq: precision nuclear run-on sequencing
RNA-seq: RNA sequencing
RT-qPCR: reverse transcription quantitative PCR
sgRNA: single-guide RNA
TF: transcription factor
TFEA: transcription factor enrichment analysis
UHPLC/MS: ultrahigh-performance liquid chromatography/mass spectrometry
WTp53: wild-type p53
