# Supplementary material for: The Δ40p53 isoform inhibits p53-dependent eRNA transcription and enables regulation by signal-specific transcription factors during p53 activation
Source: PLoS Biol. 2021 Aug 5;19(8):e3001364. doi: 10.1371/journal.pbio.3001364 (PMC8370613; doi:10.1371/journal.pbio.3001364)
Supplement: S1 Table — (PDF) [file pbio.3001364.s020.pdf]

Table S1: Basal metabolomics (DMSO CTRL) comparisons

KEY:

0.55	**Green:** significant difference (*p*≤0.05) between groups shown, metabolite ratio of < 1.00
0.76	**Light Green:** statistical significance 0.05<p<0.10, metabolite ratio of < 1.00
1.71	**Red:** significant difference (*p*≤0.05) between groups shown; metabolite ratio of ≥ 1.00
1.32	**Light Red:** statistical significance 0.05<p<0.10, metabolite ratio of ≥ 1.00
1.20	**Non-colored text and cell:** mean values not significantly different for that comparison

Super Pathway	Sub Pathway	Biochemical Name	DMSO WTp53:WTp53 WTp53	DMSO Δ40p53:WTp53 WTp53
Amino Acid	Glycine, Serine and Threonine Metabolism	glycine	1.01	1.02
		betaine	0.97	**9.94**
		serine	1.04	1.00
		N-acetylserine	1.01	**1.38**
		threonine	1.02	1.05
		N-acetylthreonine	1.93	**1.77**
	Alanine and Aspartate Metabolism	alanine	1.00	1.00
		N-acetylalanine	0.83	0.91
		aspartate	1.01	1.08
		N-acetylaspartate (NAA)	**1.36**	**1.85**
		asparagine	1.01	1.00
	Glutamate Metabolism	glutamate	1.06	1.10
		glutamine	1.07	0.93
		N-acetylglutamate	**1.33**	**1.32**
		N-acetylglutamine	1.33	1.25
		glutamate, gamma-methyl ester	1.12	1.14
		pyroglutamine*	1.07	1.20
		beta-citrylglutamate	**1.43**	**2.04**
		S-1-pyrroline-5-carboxylate	**1.88**	1.17
	Histidine Metabolism	histidine	1.03	1.03
		formiminoglutamate	1.48	0.77
		imidazole lactate	1.16	**1.33**
		4-imidazoleacetate	1.24	0.94
	Lysine Metabolism	lysine	0.98	0.99
		N6-acetyllysine	0.87	1.19
		N6-methyllysine	0.83	1.09
		N6,N6-dimethyllysine	0.79	1.02
		N6,N6,N6-trimethyllysine	0.81	1.05
		5-(galactosylhydroxy)-L-lysine	1.14	0.81
		fructosyllysine	0.99	1.08
	Phenylalanine Metabolism	phenylalanine	**1.13**	1.07
	Tyrosine Metabolism	tyrosine	1.09	1.02

		3-(4-hydroxyphenyl)lactate	1.04	**1.44**
		phenol sulfate	**2.33**	**2.85**
		O-methyltyrosine	1.11	1.07
		N-formylphenylalanine	1.52	1.10
	Tryptophan Metabolism	tryptophan	1.09	1.01
		kynurenine	**1.33**	**1.29**
	Leucine, Isoleucine and Valine Metabolism	leucine	1.06	1.02
		isovalerylcarnitine (C5)	**2.20**	1.09
		beta-hydroxyisovalerate	0.91	1.10
		isoleucine	1.11	1.01
		2-methylbutyrylcarnitine (C5)	1.33	1.01
		tiglylcarnitine (C5:1-DC)	0.88	1.24
		methylsuccinate	1.00	0.72
		valine	1.01	1.01
		alpha-hydroxyisovalerate	0.77	**1.78**
		isobutyrylcarnitine (C4)	**1.99**	**1.51**
	Methionine, Cysteine, SAM and Taurine Metabolism	methionine	1.09	0.98
		N-acetylmethionine	0.81	1.09
		N-formylmethionine	1.03	1.23
		methionine sulfoxide	0.99	**0.86**
		N-acetylmethionine sulfoxide	0.87	0.86
		S-adenosylhomocysteine (SAH)	1.06	1.20
		cystathionine	**1.98**	**2.69**
		cysteine	1.58	1.24
		N-acetylcysteine	1.13	1.15
		S-methylcysteine	1.20	0.98
		cystine	**2.15**	0.59
		cysteine sulfinic acid	1.22	0.95
		hypotaurine	1.20	**2.11**
		taurine	1.11	**1.87**
	Urea cycle; Arginine and Proline Metabolism	arginine	1.07	0.96
		argininosuccinate	1.29	**1.32**
		urea	1.35	0.88
		ornithine	**1.96**	1.41
		2-oxoarginine*	1.18	1.38
		citrulline	**2.57**	**2.34**
		proline	1.11	1.13
		dimethylarginine (SDMA + ADMA)	0.95	0.99
		trans-4-hydroxyproline	**1.41**	**1.58**
		N-monomethylarginine	1.11	1.00
	Creatine Metabolism	guanidinoacetate	**0.10**	**0.07**
		creatine	1.14	**1.73**
		creatinine	1.04	1.13

	Polyamine Metabolism	putrescine	**4.43**	1.41
		N-acetylputrescine	**2.23**	0.95
		spermidine	0.95	1.08
		(N(1) + N(8))-acetylspermidine	**7.75**	1.06
		spermine	1.08	0.86
		N(1)-acetylspermine	2.23	0.65
		5-methylthioadenosine (MTA)	1.20	1.09
	Guanidino and Acetamido Metabolism	4-guanidinobutanoate	1.01	1.06
	Glutathione Metabolism	glutathione, reduced (GSH)	0.76	**1.69**
		glutathione, oxidized (GSSG)	0.73	1.52
		cysteine-glutathione disulfide	1.01	1.00
		5-oxoproline	0.98	**1.33**
Peptide	Gamma-glutamyl Amino Acid	gamma-glutamylcysteine	8.15	1.23
		gamma-glutamylglutamine	**1.61**	1.09
		gamma-glutamylleucine	**1.74**	1.21
	Dipeptide	alanylleucine	2.25	0.88
		glutaminylleucine	2.24	0.72
		glycylleucine	**1.38**	1.02
		glycylvaline	**1.85**	0.96
		histidylalanine	1.08	0.91
		isoleucylglycine	1.22	0.99
		leucylalanine	1.12	**0.88**
		leucylglycine	1.37	0.91
		phenylalanylalanine	1.11	0.82
		phenylalanylglycine	1.10	0.89
		prolylglycine	0.97	1.28
		threonylphenylalanine	3.76	**0.70**
		tryptophylglycine	1.12	**0.96**
		tyrosylglycine	1.10	0.92
		valylglutamine	1.26	**0.98**
		valylglycine	1.40	0.96
		valylleucine	1.68	0.98
		leucylglutamine*	1.69	0.92
	Acetylated Peptides	phenylacetylglycine	1.39	**1.85**
Carbohydrate	Glycolysis, Gluconeogenesis, and Pyruvate Metabolism	glucose	1.11	1.37
		fructose 1,6-diphosphate/glucose 1,6-diphosphate/myo-inositol diphosphates	0.91	1.07
		dihydroxyacetone phosphate (DHAP)	1.06	1.64
		3-phosphoglycerate	1.17	1.25
		phosphoenolpyruvate (PEP)	1.22	1.39
		pyruvate	1.19	**1.65**
		lactate	1.19	**1.84**
		glycerate	1.14	1.17

	Pentose Phosphate Pathway	sedoheptulose-7-phosphate	0.76	1.14
	Pentose Metabolism	ribose	**1.32**	**1.54**
	Fructose, Mannose and Galactose Metabolism	mannitol/sorbitol	1.16	0.87
		mannose	0.98	0.97
		galactitol (dulcitol)	1.13	1.21
	Nucleotide Sugar	UDP-glucose/UDP-galactose	**3.41**	**2.71**
		UDP-glucuronate	0.85	**2.32**
		UDP-N-acetylglucosamine/galactosamine	1.04	**1.77**
	Aminosugar Metabolism	N-acetylglucosamine 6-phosphate	0.78	1.49
		N-acetyl-glucosamine 1-phosphate	**0.64**	1.31
		N-acetylneuraminate	0.94	1.01
		N-acetylglucosaminylasparagine	0.92	1.17
		erythronate*	1.08	**1.34**
		N-acetylglucosamine/N-acetylgalactosamine	0.92	**1.34**
		N-glycolylneuraminate	0.91	1.05
Energy	TCA Cycle	citrate	**1.38**	**1.37**
		aconitate [cis or trans]	**1.30**	**1.47**
		isocitrate	**1.51**	**1.64**
		alpha-ketoglutarate	1.05	1.12
		fumarate	1.10	1.36
		malate	1.18	**1.52**
	Oxidative Phosphorylation	acetylphosphate	1.38	1.23
		phosphate	0.90	1.11
Lipid	Long Chain Saturated Fatty Acid	myristate (14:0)	1.06	1.03
		pentadecanoate (15:0)	1.23	0.89
		palmitate (16:0)	1.20	0.96
		margarate (17:0)	**1.51**	1.08
		stearate (18:0)	**1.34**	1.07
		nonadecanoate (19:0)	**1.29**	1.09
		arachidate (20:0)	1.44	1.23
	Long Chain Monounsaturated Fatty Acid	palmitoleate (16:1n7)	**1.33**	0.96
		10-heptadecenoate (17:1n7)	**1.74**	1.07
		oleate/vaccenate (18:1)	**1.46**	1.12
		10-nonadecenoate (19:1n9)	**1.51**	1.17
		eicosenoate (20:1)	**1.41**	1.26
		erucate (22:1n9)	1.33	1.26
	Long Chain Polyunsaturated Fatty Acid (n3 and n6)	tetradecadienoate (14:2)*	**2.08**	0.99
		stearidonate (18:4n3)	**1.45**	0.88
		eicosapentaenoate (EPA; 20:5n3)	1.33	0.80
		docosapentaenoate (n3 DPA; 22:5n3)	**2.15**	1.14
		docosahexaenoate (DHA; 22:6n3)	**2.89**	1.25
		docosatrienoate (22:3n3)	**2.32**	**1.68**
		nisinate (24:6n3)	**5.24**	1.59

		hexadecadienoate (16:2n6)	**1.99**	1.18
		linoleate (18:2n6)	**1.65**	1.07
		linolenate [alpha or gamma; (18:3n3 or 6)]	**1.66**	1.01
		dihomo-linoleate (20:2n6)	**1.57**	1.21
		dihomo-linolenate (20:3n3 or n6)	**1.78**	1.16
		arachidonate (20:4n6)	**1.97**	1.11
		docosatrienoate (22:3n6)*	**2.02**	**1.46**
		adrenate (22:4n6)	**1.65**	1.01
		docosadienoate (22:2n6)	**1.54**	**1.30**
		mead acid (20:3n9)	1.17	1.01
	Fatty Acid, Branched	(14 or 15)-methylpalmitate (a17:0 or i17:0)	**1.58**	1.06
		(16 or 17)-methylstearate (a19:0 or i19:0)	**1.37**	0.97
	Fatty Acid, Dicarboxylate	glutarate (C5-DC)	1.18	1.01
		azelate (C9-DC)	0.85	**0.78**
		dodecadienoate (12:2)*	0.94	**0.93**
	Fatty Acid Metabolism (also BCAA Metabolism)	butyrylcarnitine (C4)	1.37	1.20
		propionylcarnitine (C3)	**1.44**	**1.30**
		methylmalonate (MMA)	0.96	1.27
	Fatty Acid Metabolism (Acyl Carnitine, Short Chain)	acetylcarnitine (C2)	1.08	**2.18**
	Fatty Acid Metabolism (Acyl Carnitine, Long Chain Saturated)	palmitoylcarnitine (C16)	**1.93**	1.00
		stearoylcarnitine (C18)	**2.60**	**1.27**
		lignoceroylcarnitine (C24)*	**2.17**	**1.20**
		cerotoylcarnitine (C26)*	1.18	**0.79**
	Fatty Acid Metabolism (Acyl Carnitine, Monounsaturated)	oleoylcarnitine (C18:1)	**2.27**	1.34
		nervonoylcarnitine (C24:1)*	**1.96**	**1.46**
		ximenoylcarnitine (C26:1)*	**1.24**	0.94
	Carnitine Metabolism	deoxycarnitine	**1.65**	**2.09**
		carnitine	1.00	**1.27**
	Fatty Acid, Monohydroxy	2-hydroxypalmitate	1.16	0.75
		2-hydroxyoleate	1.21	0.79
		2-hydroxystearate	**1.43**	0.88
		3-hydroxylaurate	**1.19**	1.14
		13-HODE + 9-HODE	1.11	0.97
	Endocannabinoid	oleoyl ethanolamide	**1.48**	0.98
		palmitoyl ethanolamide	1.14	1.10
		stearoyl ethanolamide	**1.50**	**1.20**
		linoleoyl ethanolamide	**1.59**	1.02
	Inositol Metabolism	myo-inositol	**0.78**	**1.79**
	Phospholipid Metabolism	choline	**1.21**	**1.36**
		choline phosphate	**0.67**	**1.33**
		glycerophosphorylcholine (GPC)	1.08	**0.83**
		phosphoethanolamine	1.46	1.21

		glycerophosphoethanolamine	1.17	**0.80**
		glycerophosphoserine*	**0.82**	**0.72**
		glycerophosphoinositol*	**0.82**	0.98
	Phosphatidylcholine (PC)	1-myristoyl-2-palmitoyl-GPC (14:0/16:0)	**1.32**	**1.12**
		1-myristoyl-2-arachidonoyl-GPC (14:0/20:4)*	**1.72**	1.15
		1,2-dipalmitoyl-GPC (16:0/16:0)	**1.54**	**1.13**
		1-palmitoyl-2-palmitoleoyl-GPC (16:0/16:1)*	**1.09**	0.91
		1-palmitoyl-2-stearoyl-GPC (16:0/18:0)	**1.95**	**1.22**
		1-palmitoyl-2-oleoyl-GPC (16:0/18:1)	**1.11**	0.96
		1-palmitoyl-2-linoleoyl-GPC (16:0/18:2)	**1.09**	**0.87**
		1-palmitoyl-2-gamma-linolenoyl-GPC (16:0/18:3n6)*	**1.27**	1.02
		1-palmitoyl-2-arachidonoyl-GPC (16:0/20:4n6)	**1.39**	0.94
		1,2-distearoyl-GPC (18:0/18:0)	**2.87**	**1.93**
		1-stearoyl-2-oleoyl-GPC (18:0/18:1)	**1.21**	1.07
		1-stearoyl-2-arachidonoyl-GPC (18:0/20:4)	**1.71**	1.11
		1-oleoyl-2-linoleoyl-GPC (18:1/18:2)*	**1.09**	0.95
		1-oleoyl-2-docosahexaenoyl-GPC (18:1/22:6)*	**2.19**	**1.23**
		1,2-dilinoleoyl-GPC (18:2/18:2)	**1.23**	**0.86**
		1-linoleoyl-2-linolenoyl-GPC (18:2/18:3)*	**1.29**	**0.77**
	Phosphatidylethanolamine (PE)	1,2-dipalmitoyl-GPE (16:0/16:0)*	**1.16**	**0.87**
		1-palmitoyl-2-oleoyl-GPE (16:0/18:1)	0.91	**0.83**
		1-palmitoyl-2-linoleoyl-GPE (16:0/18:2)	**0.81**	**0.77**
		1-palmitoleoyl-2-oleoyl-GPE (16:1/18:1)*	0.86	**0.82**
		1-stearoyl-2-oleoyl-GPE (18:0/18:1)	**1.12**	1.06
		1-stearoyl-2-linoleoyl-GPE (18:0/18:2)*	0.95	**0.77**
		1-stearoyl-2-arachidonoyl-GPE (18:0/20:4)	**1.28**	0.91
		1,2-dioleoyl-GPE (18:1/18:1)	0.98	**1.16**
		1-oleoyl-2-linoleoyl-GPE (18:1/18:2)*	**0.82**	**0.83**
		1-oleoyl-2-arachidonoyl-GPE (18:1/20:4)*	**1.43**	1.06
	Phosphatidylserine (PS)	1-palmitoyl-2-oleoyl-GPS (16:0/18:1)	0.91	**0.77**
		1-stearoyl-2-oleoyl-GPS (18:0/18:1)	**1.24**	**1.18**
		1-stearoyl-2-linoleoyl-GPS (18:0/18:2)	0.92	**0.74**
	Phosphatidylglycerol (PG)	1-palmitoyl-2-oleoyl-GPG (16:0/18:1)	**0.79**	**0.82**
		1,2-distearoyl-GPG (18:0/18:0)	**1.48**	**1.38**
		1-stearoyl-2-oleoyl-GPG (18:0/18:1)	**1.31**	**1.25**
	Phosphatidylinositol (PI)	1-palmitoyl-2-oleoyl-GPI (16:0/18:1)*	0.98	**0.79**
		1-palmitoyl-2-arachidonoyl-GPI (16:0/20:4)*	**1.54**	**0.78**
		1-stearoyl-2-oleoyl-GPI (18:0/18:1)*	0.99	**0.79**
		1-stearoyl-2-linoleoyl-GPI (18:0/18:2)	1.01	**0.80**
		1,2-dioleoyl-GPI (18:1/18:1)	**0.77**	0.93
		1-stearoyl-2-arachidonoyl-GPI (18:0/20:4)	**1.29**	**1.15**
		1-oleoyl-2-arachidonoyl-GPI (18:1/20:4)*	**1.25**	1.02
	Lysophospholipid	1-palmitoyl-GPC (16:0)	**0.77**	**0.70**

	2-palmitoyl-GPC (16:0)*	1.35	0.78
	1-palmitoleoyl-GPC (16:1)*	**0.59**	0.77
	2-palmitoleoyl-GPC (16:1)*	1.31	0.76
	1-stearoyl-GPC (18:0)	0.85	**0.80**
	1-oleoyl-GPC (18:1)	1.12	0.79
	1-linoleoyl-GPC (18:2)	1.34	0.71
	1-lignoceroyl-GPC (24:0)	**0.81**	**0.79**
	1-palmitoyl-GPE (16:0)	**0.70**	**0.63**
	1-stearoyl-GPE (18:0)	**0.81**	**0.72**
	2-stearoyl-GPE (18:0)*	1.16	0.85
	1-oleoyl-GPE (18:1)	0.91	0.75
	1-linoleoyl-GPE (18:2)*	0.99	0.73
	1-arachidonoyl-GPE (20:4n6)*	**1.69**	0.88
	1-stearoyl-GPS (18:0)*	1.08	0.73
	1-oleoyl-GPS (18:1)	1.23	0.80
	1-palmitoyl-GPG (16:0)*	1.01	0.74
	1-stearoyl-GPG (18:0)	1.13	0.96
	1-oleoyl-GPG (18:1)*	**1.50**	1.04
	1-linoleoyl-GPG (18:2)*	**2.07**	1.06
	1-palmitoyl-GPI (16:0)	0.91	0.73
	1-stearoyl-GPI (18:0)	0.85	0.87
	1-oleoyl-GPI (18:1)	0.96	0.76
	1-linoleoyl-GPI (18:2)*	1.05	**0.68**
	1-arachidonoyl-GPI (20:4)*	**1.40**	0.90
Plasmalogen	1-(1-enyl-palmitoyl)-2-oleoyl-GPE (P-16:0/18:1)*	**1.08**	0.94
	1-(1-enyl-palmitoyl)-2-linoleoyl-GPE (P-16:0/18:2)*	1.01	**0.75**
	1-(1-enyl-palmitoyl)-2-palmitoyl-GPC (P-16:0/16:0)*	**1.22**	1.00
	1-(1-enyl-palmitoyl)-2-palmitoleoyl-GPC (P-16:0/16:1)*	1.08	0.97
	1-(1-enyl-palmitoyl)-2-arachidonoyl-GPE (P-16:0/20:4)*	**1.25**	1.09
	1-(1-enyl-palmitoyl)-2-oleoyl-GPC (P-16:0/18:1)*	**1.17**	0.99
	1-(1-enyl-stearoyl)-2-oleoyl-GPE (P-18:0/18:1)	**1.27**	**1.17**
	1-(1-enyl-stearoyl)-2-linoleoyl-GPE (P-18:0/18:2)*	**1.32**	**0.90**
	1-(1-enyl-palmitoyl)-2-arachidonoyl-GPC (P-16:0/20:4)*	**1.90**	**2.25**
	1-(1-enyl-palmitoyl)-2-linoleoyl-GPC (P-16:0/18:2)*	**1.22**	**0.87**
	1-(1-enyl-stearoyl)-2-arachidonoyl-GPE (P-18:0/20:4)*	**1.68**	**1.36**
Lysoplasmalogen	1-(1-enyl-palmitoyl)-GPC (P-16:0)*	**0.80**	**0.81**
	1-(1-enyl-palmitoyl)-GPE (P-16:0)*	**0.72**	**0.72**
	1-(1-enyl-oleoyl)-GPE (P-18:1)*	**0.79**	**0.73**
	1-(1-enyl-stearoyl)-GPE (P-18:0)*	0.94	**0.86**
	1-(1-enyl-oleoyl)-2-oleoyl-GPE (P-18:1/18:1)*	**0.83**	0.88
Glycerolipid Metabolism	glycerol	**1.34**	**1.43**
	glycerol 3-phosphate	0.98	1.23
	glycerophosphoglycerol	**1.30**	0.99

		1-pentadecanoylglycerol (15:0)	**1.63**	**1.99**
		1-palmitoylglycerol (16:0)	1.57	0.79
		1-margaroylglycerol (17:0)	1.50	1.00
		1-oleoylglycerol (18:1)	1.66	1.01
		1-linoleoylglycerol (18:2)	1.69	1.15
		2-dihomo-linoleoylglycerol (20:2)*	1.42	0.93
		1-dihomo-linolenylglycerol (20:3)	1.60	0.76
		1-arachidonylglycerol (20:4)	**2.08**	1.41
	Monoacylglycerol	2-palmitoylglycerol (16:0)	**2.02**	0.87
		2-palmitoleoylglycerol (16:1)*	1.98	0.61
		2-oleoylglycerol (18:1)	2.38	0.85
		2-linoleoylglycerol (18:2)	**2.14**	1.03
		2-arachidonoylglycerol (20:4)	**2.23**	0.85
		1-heptadecenoylglycerol (17:1)*	**2.13**	1.14
		2-heptadecenoylglycerol (17:1)*	2.03	0.93
		1-meadoylglycerol (20:3n9)*	1.38	0.51
		1-dihomo-linoleoylglycerol (20:2)	1.31	1.18
	Diacylglycerol	diacylglycerol (14:0/18:1, 16:0/16:1) [2]*	0.93	0.93
		diacylglycerol (16:1/18:2 [2], 16:0/18:3 [1])*	1.09	**0.79**
		palmitoyl-oleoyl-glycerol (16:0/18:1) [2]*	0.99	0.98
		palmitoyl-linoleoyl-glycerol (16:0/18:2) [2]*	**1.51**	1.04
		palmitoleoyl-oleoyl-glycerol (16:1/18:1) [2]*	0.65	0.76
		palmitoyl-arachidonoyl-glycerol (16:0/20:4) [2]*	**1.96**	1.11
		oleoyl-oleoyl-glycerol (18:1/18:1) [2]*	**1.24**	**1.21**
		oleoyl-linoleoyl-glycerol (18:1/18:2) [1]	1.18	0.89
		oleoyl-linoleoyl-glycerol (18:1/18:2) [2]	1.09	1.02
		linoleoyl-linolenoyl-glycerol (18:2/18:3) [2]*	**1.60**	0.80
		stearoyl-arachidonoyl-glycerol (18:0/20:4) [1]*	**1.82**	1.26
		stearoyl-arachidonoyl-glycerol (18:0/20:4) [2]*	**1.42**	1.11
		oleoyl-arachidonoyl-glycerol (18:1/20:4) [2]*	**1.67**	1.12
	Sphingolipid Synthesis	sphinganine	**0.75**	0.89
		sphingadienine	**0.58**	**0.63**
		phytosphingosine	**0.63**	0.83
	Dihydroceramides	N-palmitoyl-sphinganine (d18:0/16:0)	**1.40**	**1.58**
	Ceramides	N-palmitoyl-sphingosine (d18:1/16:0)	1.01	1.01
		N-stearoyl-sphingosine (d18:1/18:0)*	**1.33**	**1.35**
		N-palmitoyl-sphingadienine (d18:2/16:0)*	**1.13**	**0.74**
		N-palmitoyl-heptadecasphingosine (d17:1/16:0)*	1.05	0.95
		ceramide (d18:1/14:0, d16:1/16:0)*	0.99	0.97
		ceramide (d18:1/17:0, d17:1/18:0)*	**1.51**	**1.29**
	Hexosylceramides (HCER)	glycosyl-N-palmitoyl-sphingosine (d18:1/16:0)	1.03	**0.63**
		glycosyl-N-stearoyl-sphingosine (d18:1/18:0)	**1.33**	0.93
		glycosyl ceramide (d18:1/20:0, d16:1/22:0)*	1.34	0.89

		glycosyl ceramide (d18:2/24:1, d18:1/24:2)*	1.18	**0.68**
	Lactosylceramides (LCER)	lactosyl-N-palmitoyl-sphingosine (d18:1/16:0)	**0.83**	**0.69**
		lactosyl-N-nervonoyl-sphingosine (d18:1/24:1)*	**0.75**	0.87
	Dihydrosphingomyelins	myristoyl dihydrosphingomyelin (d18:0/14:0)*	1.14	**1.51**
		palmitoyl dihydrosphingomyelin (d18:0/16:0)*	**1.19**	**1.46**
		behenoyl dihydrosphingomyelin (d18:0/22:0)*	**1.83**	**2.35**
		sphingomyelin (d18:0/18:0, d19:0/17:0)*	**1.44**	**2.80**
		sphingomyelin (d18:0/20:0, d16:0/22:0)*	**1.54**	**2.71**
	Sphingomyelins	palmitoyl sphingomyelin (d18:1/16:0)	1.01	1.01
		hydroxypalmitoyl sphingomyelin (d18:1/16:0(OH))**	**1.38**	**1.22**
		stearoyl sphingomyelin (d18:1/18:0)	**1.31**	**1.42**
		behenoyl sphingomyelin (d18:1/22:0)*	1.18	**1.28**
		lignoceroyl sphingomyelin (d18:1/24:0)	**1.18**	**1.28**
		sphingomyelin (d18:2/24:2)*	**1.42**	0.81
		sphingomyelin (d17:1/14:0, d16:1/15:0)*	1.15	1.10
		sphingomyelin (d18:1/14:0, d16:1/16:0)*	1.08	**1.13**
		sphingomyelin (d18:2/14:0, d18:1/14:1)*	**1.42**	1.15
		sphingomyelin (d17:1/16:0, d18:1/15:0, d16:1/17:0)*	1.07	0.99
		sphingomyelin (d18:2/16:0, d18:1/16:1)*	**1.20**	**0.88**
		sphingomyelin (d18:1/17:0, d17:1/18:0, d19:1/16:0)	**1.60**	1.12
		sphingomyelin (d18:1/18:1, d18:2/18:0)	**1.95**	**1.84**
		sphingomyelin (d18:1/20:0, d16:1/22:0)*	**1.14**	**1.23**
		sphingomyelin (d18:1/21:0, d17:1/22:0, d16:1/23:0)*	1.52	1.03
		sphingomyelin (d18:1/22:1, d18:2/22:0, d16:1/24:1)*	0.98	1.00
		sphingomyelin (d18:2/23:0, d18:1/23:1, d17:1/24:1)*	1.06	1.06
		sphingomyelin (d18:1/24:1, d18:2/24:0)*	0.93	1.18
		sphingomyelin (d18:2/24:1, d18:1/24:2)*	1.07	1.05
		sphingomyelin (d18:1/25:0, d19:0/24:1, d20:1/23:0, d19:1/24:0)*	**1.46**	1.08
	Ceramide PEs	palmitoyl-sphingosine-phosphoethanolamine (d18:1/16:0)	**1.40**	1.05
	Sphingosines	sphingosine	**0.55**	**0.77**
		hexadecasphingosine (d16:1)*	**0.63**	**0.73**
		heptadecasphingosine (d17:1)	**0.66**	**0.77**
		eicosanoylsphingosine (d20:1)*	**0.81**	0.93
	Mevalonate Metabolism	3-hydroxy-3-methylglutarate	**2.75**	**2.48**
	Sterol	cholesterol	0.99	0.98
		4-cholesten-3-one	0.91	0.85
		7-hydroxycholesterol (alpha or beta)	1.02	0.81
Nucleotide	Purine Metabolism, (Hypo)Xanthine/Inosine containing	AICA ribonucleotide	1.33	1.34
		inosine	0.96	**1.41**
		hypoxanthine	0.88	**1.50**
		xanthine	**0.83**	**0.64**
		2'-deoxyinosine	21.94	0.98
		urate	0.99	**0.40**

	Purine Metabolism, Adenine containing	adenosine 5'-triphosphate (ATP)	1.30	**1.35**
		adenosine 5'-monophosphate (AMP)	**0.58**	0.93
		adenosine 3'-monophosphate (3'-AMP)	0.40	0.79
		adenosine 2'-monophosphate (2'-AMP)	0.65	0.63
		adenosine	**1.42**	**1.31**
		adenine	10.35	1.30
		N6-methyladenosine	1.16	1.29
		N6,N6-dimethyladenosine	**1.46**	1.29
		2'-deoxyadenosine	769.75	2.21
	Purine Metabolism, Guanine containing	guanosine 5'- diphosphate (GDP)	0.99	**1.49**
		guanosine 5'- monophosphate (5'-GMP)	**0.45**	0.92
		guanosine 3'-monophosphate (3'-GMP)	**0.39**	0.82
		guanosine	1.34	**2.54**
		guanine	**1.70**	**6.78**
		7-methylguanine	1.06	0.98
		2'-deoxyguanosine	38.31	1.25
	Pyrimidine Metabolism, Orotate containing	orotate	**2.23**	0.78
	Pyrimidine Metabolism, Uracil containing	uridine 3'-monophosphate (3'-UMP)	0.51	0.79
		uridine	0.95	**1.19**
		uracil	0.85	1.62
		pseudouridine	0.86	1.36
		3-ureidopropionate	1.38	1.41
	Pyrimidine Metabolism, Cytidine containing	cytidine 5'-monophosphate (5'-CMP)	1.03	**1.28**
		cytidine 2' or 3'-monophosphate (2' or 3'-CMP)	**0.52**	**0.75**
		cytidine	0.96	1.35
		cytosine	1.22	1.06
		2'-deoxycytidine 5'-monophosphate	15.71	1.00
		2'-deoxycytidine	**65.21**	0.88
		2'-O-methylcytidine	0.67	1.42
	Pyrimidine Metabolism, Thymine containing	thymidine 5'-monophosphate	1.00	1.00
		thymidine	**1.40**	1.00
	Purine and Pyrimidine Metabolism	methylphosphate	0.85	0.90
Cofactors and Vitamins	Nicotinate and Nicotinamide Metabolism	nicotinamide	0.93	**1.34**
		nicotinamide ribonucleotide (NMN)	1.09	0.96
		nicotinamide riboside	1.26	1.25
		nicotinamide adenine dinucleotide (NAD+)	0.82	0.84
		1-methylnicotinamide	1.07	1.08
		adenosine 5'-diphosphoribose (ADP-ribose)	0.88	1.13
	Riboflavin Metabolism	riboflavin (Vitamin B2)	1.08	0.98
		flavin adenine dinucleotide (FAD)	1.02	1.07
	Pantothenate and CoA Metabolism	pantothenate	1.09	**1.25**
		pantetheine	1.03	**1.25**

		phosphopantetheine	0.98	**1.48**
	Tocopherol Metabolism	alpha-tocopherol	**1.13**	0.92
		gamma-tocopherol/beta-tocopherol	1.03	**0.60**
	Hemoglobin and Porphyrin Metabolism	heme	0.86	1.41
	Thiamine Metabolism	thiamin (Vitamin B1)	1.04	**1.48**
	Vitamin A Metabolism	retinol (Vitamin A)	**1.39**	1.16
	Vitamin B6 Metabolism	pyridoxine (Vitamin B6)	1.22	1.37
		pyridoxamine	1.09	1.16
		pyridoxamine phosphate	0.94	1.00
		pyridoxal phosphate	0.82	1.00
		pyridoxal	**1.33**	**1.41**
Xenobiotics	Benzoate Metabolism	hippurate	1.18	**1.54**
		p-cresol sulfate	**0.79**	**1.38**
	Food Component/Plant	3-formylindole	**0.65**	1.07
		ergothioneine	0.77	**2.05**
		stachydrine	0.75	0.93
		methyl glucopyranoside (alpha + beta)	**1.41**	0.96
	Drug - Antibiotic	penicillin G	1.16	**1.57**
	Drug - Antineoplastic	tamoxifen	1.00	1.00
	Chemical	HEPES	1.09	**1.34**
		2,4-di-tert-butylphenol	1.16	0.92
		phenol red	1.25	**1.59**
		thioproline	1.13	0.98
		4-chlorobenzoic acid	1.06	1.03
