# Supplementary material for: The Δ40p53 isoform inhibits p53-dependent eRNA transcription and enables regulation by signal-specific transcription factors during p53 activation
Source: PLoS Biol. 2021 Aug 5;19(8):e3001364. doi: 10.1371/journal.pbio.3001364 (PMC8370613; doi:10.1371/journal.pbio.3001364)
Supplement: S2 Table — (PDF) [file pbio.3001364.s021.pdf]

Table S2: Metabolomics comparisons in p53-activated cells (+Nutlin, 20 hr)

KEY:

0.55	**Green:** significant difference ($p \leq 0.05$) between groups shown, metabolite ratio of < 1.00
0.76	**Light Green:** statistical significance 0.05<p<0.10, metabolite ratio of < 1.00
1.71	**Red:** significant difference ($p \leq 0.05$) between groups shown; metabolite ratio of ≥ 1.00
1.32	**Light Red:** statistical significance 0.05<p<0.10, metabolite ratio of ≥ 1.00
1.20	**Non-colored text and cell:** mean values not significantly different for that comparison

Super Pathway	Sub Pathway	Biochemical Name	Nutlin WTp53:WTp53 WTp53	Nutlin Δ40p53:WTp53 WTp53
Amino Acid	Glycine, Serine and Threonine Metabolism	glycine	1.17	1.03
		betaine	0.80	**11.69**
		serine	**1.20**	1.01
		N-acetylserine	1.00	1.13
		threonine	1.13	1.03
		N-acetylthreonine	**1.58**	1.47
	Alanine and Aspartate Metabolism	alanine	1.08	0.95
		N-acetylalanine	0.78	0.83
		aspartate	**0.86**	0.88
		N-acetylaspartate (NAA)	**1.81**	**2.31**
		asparagine	1.07	0.88
	Glutamate Metabolism	glutamate	**1.25**	1.01
		glutamine	0.90	0.75
		N-acetylglutamate	1.01	0.85
		N-acetylglutamine	0.85	0.78
		glutamate, gamma-methyl ester	0.82	**0.70**
		pyroglutamine*	0.87	**0.75**
		beta-citrylglutamate	**1.33**	**1.56**
		S-1-pyrroline-5-carboxylate	**1.71**	1.10
	Histidine Metabolism	histidine	0.96	0.88
		formiminoglutamate	**1.52**	1.23
		imidazole lactate	**0.59**	0.89
		4-imidazoleacetate	0.75	0.68
	Lysine Metabolism	lysine	1.05	0.84
		N6-acetyllysine	1.14	1.10
		N6-methyllysine	1.14	1.02
		N6,N6-dimethyllysine	1.18	0.94
		N6,N6,N6-trimethyllysine	1.12	0.98
		5-(galactosylhydroxy)-L-lysine	0.99	**0.51**
		fructosyllysine	0.83	0.74
	Phenylalanine Metabolism	phenylalanine	1.12	0.96
Amin Acid	Tyrosine Metabolism	tyrosine	1.13	0.97

	3-(4-hydroxyphenyl)lactate	0.95	1.02
	phenol sulfate	**3.71**	**2.78**
	O-methyltyrosine	0.90	0.81
	N-formylphenylalanine	1.32	1.44
Tryptophan Metabolism	tryptophan	0.95	0.91
	kynurenine	1.07	1.04
Leucine, Isoleucine and Valine Metabolism	leucine	1.09	0.93
	isovalerylcarnitine (C5)	**1.72**	1.10
	beta-hydroxyisovalerate	1.21	1.03
	isoleucine	1.09	0.91
	2-methylbutyrylcarnitine (C5)	**1.50**	0.81
	tiglylcarnitine (C5:1-DC)	1.01	1.16
	methylsuccinate	1.25	0.80
	valine	1.05	0.98
	alpha-hydroxyisovalerate	0.99	0.87
	isobutyrylcarnitine (C4)	**2.02**	**1.42**
Methionine, Cysteine, SAM and Taurine Metabolism	methionine	1.03	0.89
	N-acetylmethionine	0.92	1.02
	N-formylmethionine	1.03	1.00
	methionine sulfoxide	**1.22**	0.90
	N-acetylmethionine sulfoxide	1.14	0.87
	S-adenosylhomocysteine (SAH)	0.98	0.96
	cystathionine	0.68	**1.68**
	cysteine	1.05	0.83
	N-acetylcysteine	1.15	1.15
	S-methylcysteine	1.19	0.99
	cystine	1.04	0.60
	cysteine sulfinic acid	**1.24**	0.89
	hypotaurine	**0.41**	1.07
	taurine	0.89	1.04
Urea cycle; Arginine and Proline Metabolism	arginine	1.06	**0.82**
	argininosuccinate	1.11	1.04
	urea	**2.31**	**1.36**
	ornithine	**3.05**	1.27
	2-oxoarginine*	0.74	0.82
	citrulline	1.52	**2.55**
	proline	0.96	0.92
	dimethylarginine (SDMA + ADMA)	1.13	0.92
	trans-4-hydroxyproline	1.14	1.07
	N-monomethylarginine	**1.18**	0.94
Creatine Metabolism	guanidinoacetate	**0.10**	**0.08**
	creatine	0.77	0.99
	creatinine	0.88	0.87

Amino Acid	Polyamine Metabolism	putrescine	1.63	1.01
		N-acetylputrescine	**3.07**	**1.48**
		spermidine	0.49	0.85
		(N(1) + N(8))-acetylspermidine	**1.62**	**0.23**
		spermine	0.60	0.90
		N(1)-acetylspermine	0.63	**0.21**
		5-methylthioadenosine (MTA)	0.95	0.82
	Guanidino and Acetamido Metabolism	4-guanidinobutanoate	1.13	1.00
	Glutathione Metabolism	glutathione, reduced (GSH)	**0.44**	0.98
		glutathione, oxidized (GSSG)	0.62	1.03
		cysteine-glutathione disulfide	0.94	0.83
		5-oxoproline	0.95	0.95
Peptide	Gamma-glutamyl Amino Acid	gamma-glutamylcysteine	0.48	0.17
		gamma-glutamylglutamine	1.05	**0.50**
		gamma-glutamylleucine	1.36	1.01
	Dipeptide	alanylleucine	0.97	**0.48**
		glutaminylleucine	1.01	0.89
		glycylleucine	**1.49**	1.03
		glycylvaline	0.99	0.79
		histidylalanine	1.13	0.91
		isoleucylglycine	1.17	0.84
		leucylalanine	1.44	**0.87**
		leucylglycine	1.55	**0.94**
		phenylalanylalanine	1.14	**0.74**
		phenylalanylglycine	1.24	0.97
		prolylglycine	0.95	0.86
		threonylphenylalanine	**1.27**	0.76
		tryptophylglycine	1.16	**0.83**
		tyrosylglycine	**1.51**	1.08
		valylglutamine	1.46	**0.84**
		valylglycine	1.32	0.90
		valylleucine	**1.66**	0.77
		leucylglutamine*	1.20	**0.70**
	Acetylated Peptides	phenylacetylglycine	0.99	1.05
Carbohydrate	Glycolysis, Gluconeogenesis, and Pyruvate Metabolism	glucose	0.90	0.70
		fructose 1,6-diphosphate/glucose 1,6-diphosphate/myo-inositol diphosphates	1.12	1.13
		dihydroxyacetone phosphate (DHAP)	1.12	1.40
		3-phosphoglycerate	0.98	1.02
		phosphoenolpyruvate (PEP)	0.84	1.08
		pyruvate	0.81	1.23
		lactate	1.08	1.14
		glycerate	0.78	0.99

	Pentose Phosphate Pathway	sedoheptulose-7-phosphate	0.90	1.00
	Pentose Metabolism	ribose	1.12	1.14
	Fructose, Mannose and Galactose Metabolism	mannitol/sorbitol	0.89	**2.38**
		mannose	1.11	0.96
		galactitol (dulcitol)	0.87	1.01
	Nucleotide Sugar	UDP-glucose/UDP-galactose	**2.07**	1.17
		UDP-glucuronate	0.89	**1.37**
		UDP-N-acetylglucosamine/galactosamine	0.82	1.24
Carbohydrate	Aminosugar Metabolism	N-acetylglucosamine 6-phosphate	**0.63**	1.24
		N-acetyl-glucosamine 1-phosphate	1.19	1.04
		N-acetylneuraminate	0.95	**1.14**
		N-acetylglucosaminylasparagine	0.91	1.06
		erythronate*	1.02	1.05
		N-acetylglucosamine/N-acetylgalactosamine	0.87	1.12
		N-glycolylneuraminate	1.14	1.13
Energy	TCA Cycle	citrate	**1.26**	1.01
		aconitate [cis or trans]	**1.39**	1.22
		isocitrate	1.52	1.14
		alpha-ketoglutarate	1.14	1.03
		fumarate	0.84	1.20
		malate	0.99	1.29
	Oxidative Phosphorylation	acetylphosphate	1.08	0.77
		phosphate	1.04	0.99
Lipid	Long Chain Saturated Fatty Acid	myristate (14:0)	0.89	0.84
		pentadecanoate (15:0)	1.03	0.79
		palmitate (16:0)	0.97	0.77
		margarate (17:0)	1.21	0.85
		stearate (18:0)	1.05	0.81
		nonadecanoate (19:0)	1.08	0.85
		arachidate (20:0)	0.95	0.99
	Long Chain Monounsaturated Fatty Acid	palmitoleate (16:1n7)	0.92	0.85
		10-heptadecenoate (17:1n7)	1.08	0.91
		oleate/vaccenate (18:1)	0.94	0.94
		10-nonadecenoate (19:1n9)	1.07	0.98
		eicosenoate (20:1)	0.89	1.15
		erucate (22:1n9)	0.86	1.21
	Long Chain Polyunsaturated Fatty Acid (n3 and n6)	tetradecadienoate (14:2)*	**1.77**	0.96
		stearidonate (18:4n3)	0.95	**0.74**
		eicosapentaenoate (EPA; 20:5n3)	0.90	**0.64**
		docosapentaenoate (n3 DPA; 22:5n3)	1.18	0.98
		docosahexaenoate (DHA; 22:6n3)	**1.59**	1.03
		docosatrienoate (22:3n3)	1.37	**1.41**

Lipid	Long Chain Polyunsaturated Fatty Acid (n3 and n6)	nisinate (24:6n3)	**1.70**	1.19
		hexadecadienoate (16:2n6)	0.86	0.99
		linoleate (18:2n6)	1.00	0.99
		linolenate [alpha or gamma; (18:3n3 or 6)]	1.07	0.91
		dihomo-linoleate (20:2n6)	1.07	1.11
		dihomo-linolenate (20:3n3 or n6)	1.11	1.01
		arachidonate (20:4n6)	1.21	1.05
		docosatrienoate (22:3n6)*	1.08	1.19
		adrenate (22:4n6)	1.07	1.06
		docosadienoate (22:2n6)	1.07	1.11
		mead acid (20:3n9)	0.82	0.87
	Fatty Acid, Branched	(14 or 15)-methylpalmitate (a17:0 or i17:0)	1.10	0.86
		(16 or 17)-methylstearate (a19:0 or i19:0)	1.11	0.77
	Fatty Acid, Dicarboxylate	glutarate (C5-DC)	1.41	0.59
		azelate (C9-DC)	0.99	0.91
		dodecadienoate (12:2)*	1.00	1.00
	Fatty Acid Metabolism (also BCAA Metabolism)	butyrylcarnitine (C4)	1.14	0.93
		propionylcarnitine (C3)	**1.90**	1.21
		methylmalonate (MMA)	0.94	1.14
	Fatty Acid Metabolism (Acyl Carnitine, Short Chain)	acetylcarnitine (C2)	1.13	**1.48**
	Fatty Acid Metabolism (Acyl Carnitine, Long Chain Saturated)	palmitoylcarnitine (C16)	**1.86**	1.19
		stearoylcarnitine (C18)	**2.85**	**1.45**
		lignoceroylcarnitine (C24)*	**2.67**	**2.01**
		cerotoylcarnitine (C26)*	**1.44**	**1.38**
	Fatty Acid Metabolism (Acyl Carnitine, Monounsaturated)	oleoylcarnitine (C18:1)	**3.00**	**1.92**
		nervonoylcarnitine (C24:1)*	**2.26**	**2.52**
		ximenoylcarnitine (C26:1)*	**1.55**	**1.67**
	Carnitine Metabolism	deoxycarnitine	**1.48**	1.32
		carnitine	0.96	0.99
	Fatty Acid, Monohydroxy	2-hydroxypalmitate	1.31	1.04
		2-hydroxyoleate	1.22	1.15
		2-hydroxystearate	1.60	1.28
		3-hydroxylaurate	**1.31**	0.96
		13-HODE + 9-HODE	1.13	1.01
	Endocannabinoid	oleoyl ethanolamide	1.10	0.94
		palmitoyl ethanolamide	1.12	1.03
		stearoyl ethanolamide	**1.45**	**1.20**
		linoleoyl ethanolamide	1.04	0.95
	Inositol Metabolism	myo-inositol	0.78	**1.37**
	Phospholipid Metabolism	choline	1.11	**1.38**
		choline phosphate	**0.62**	1.10

		glycerophosphorylcholine (GPC)	**1.19**	**0.78**
		phosphoethanolamine	**1.93**	1.24
		glycerophosphoethanolamine	**1.36**	**0.76**
		glycerophosphoserine*	1.09	**0.73**
		glycerophosphoinositol*	1.07	0.97
	Phosphatidylcholine (PC)	1-myristoyl-2-palmitoyl-GPC (14:0/16:0)	**1.22**	0.99
		1-myristoyl-2-arachidonoyl-GPC (14:0/20:4)*	**1.45**	0.97
		1,2-dipalmitoyl-GPC (16:0/16:0)	**1.36**	1.01
		1-palmitoyl-2-palmitoleoyl-GPC (16:0/16:1)*	1.07	**0.80**
		1-palmitoyl-2-stearoyl-GPC (16:0/18:0)	**1.91**	**1.16**
		1-palmitoyl-2-oleoyl-GPC (16:0/18:1)	**1.17**	**0.86**
		1-palmitoyl-2-linoleoyl-GPC (16:0/18:2)	1.08	**0.76**
		1-palmitoyl-2-gamma-linolenoyl-GPC (16:0/18:3n6)*	1.16	0.93
		1-palmitoyl-2-arachidonoyl-GPC (16:0/20:4n6)	**1.18**	**0.78**
	Phosphatidylcholine (PC)	1,2-distearoyl-GPC (18:0/18:0)	**1.81**	**2.09**
		1-stearoyl-2-oleoyl-GPC (18:0/18:1)	**1.22**	0.98
		1-stearoyl-2-arachidonoyl-GPC (18:0/20:4)	**1.35**	**0.81**
		1-oleoyl-2-linoleoyl-GPC (18:1/18:2)*	1.09	**0.81**
		1-oleoyl-2-docosahexaenoyl-GPC (18:1/22:6)*	**1.61**	0.95
		1,2-dilinoleoyl-GPC (18:2/18:2)	1.10	**0.78**
		1-linoleoyl-2-linolenoyl-GPC (18:2/18:3)*	1.10	**0.76**
	Phosphatidylethanolamine (PE)	1,2-dipalmitoyl-GPE (16:0/16:0)*	**1.16**	0.93
		1-palmitoyl-2-oleoyl-GPE (16:0/18:1)	**0.88**	**0.73**
		1-palmitoyl-2-linoleoyl-GPE (16:0/18:2)	**0.89**	**0.75**
		1-palmitoleoyl-2-oleoyl-GPE (16:1/18:1)*	**0.67**	**0.61**
Lipid		1-stearoyl-2-oleoyl-GPE (18:0/18:1)	**1.11**	0.94
		1-stearoyl-2-linoleoyl-GPE (18:0/18:2)*	0.96	**0.70**
		1-stearoyl-2-arachidonoyl-GPE (18:0/20:4)	**1.17**	**0.83**
		1,2-dioleoyl-GPE (18:1/18:1)	0.92	0.93
		1-oleoyl-2-linoleoyl-GPE (18:1/18:2)*	**0.89**	**0.78**
		1-oleoyl-2-arachidonoyl-GPE (18:1/20:4)*	**1.26**	0.90
	Phosphatidylserine (PS)	1-palmitoyl-2-oleoyl-GPS (16:0/18:1)	1.00	**0.81**
		1-stearoyl-2-oleoyl-GPS (18:0/18:1)	**1.28**	**1.24**
		1-stearoyl-2-linoleoyl-GPS (18:0/18:2)	0.99	**0.76**
	Phosphatidylglycerol (PG)	1-palmitoyl-2-oleoyl-GPG (16:0/18:1)	0.97	0.93
		1,2-distearoyl-GPG (18:0/18:0)	**1.60**	**1.27**
		1-stearoyl-2-oleoyl-GPG (18:0/18:1)	**1.34**	1.07
	Phosphatidylinositol (PI)	1-palmitoyl-2-oleoyl-GPI (16:0/18:1)*	1.11	**0.67**
		1-palmitoyl-2-arachidonoyl-GPI (16:0/20:4)*	**1.23**	**0.79**
		1-stearoyl-2-oleoyl-GPI (18:0/18:1)*	1.09	**0.80**
		1-stearoyl-2-linoleoyl-GPI (18:0/18:2)	1.03	**0.74**
		1,2-dioleoyl-GPI (18:1/18:1)	**0.81**	**0.85**
		1-stearoyl-2-arachidonoyl-GPI (18:0/20:4)	**1.31**	1.10

		1-oleoyl-2-arachidonoyl-GPI (18:1/20:4)*	1.15	0.92
	Lysophospholipid	1-palmitoyl-GPC (16:0)	0.70	0.66
		2-palmitoyl-GPC (16:0)*	0.86	0.79
		1-palmitoleoyl-GPC (16:1)*	0.49	0.43
		2-palmitoleoyl-GPC (16:1)*	1.10	1.05
		1-stearoyl-GPC (18:0)	0.82	0.71
		1-oleoyl-GPC (18:1)	0.72	0.50
		1-linoleoyl-GPC (18:2)	0.60	0.59
		1-lignoceroyl-GPC (24:0)	0.84	0.72
		1-palmitoyl-GPE (16:0)	0.61	0.61
		1-stearoyl-GPE (18:0)	0.75	0.66
		2-stearoyl-GPE (18:0)*	0.85	0.49
		1-oleoyl-GPE (18:1)	0.60	0.72
		1-linoleoyl-GPE (18:2)*	0.74	0.67
		1-arachidonoyl-GPE (20:4n6)*	1.11	0.70
		1-stearoyl-GPS (18:0)*	0.77	0.66
		1-oleoyl-GPS (18:1)	0.56	0.60
	Lysophospholipid	1-palmitoyl-GPG (16:0)*	0.82	0.75
		1-stearoyl-GPG (18:0)	0.94	0.85
		1-oleoyl-GPG (18:1)*	1.05	0.84
		1-linoleoyl-GPG (18:2)*	1.37	0.84
		1-palmitoyl-GPI (16:0)	0.58	0.62
		1-stearoyl-GPI (18:0)	0.63	0.67
		1-oleoyl-GPI (18:1)	0.66	0.66
		1-linoleoyl-GPI (18:2)*	0.82	0.65
		1-arachidonoyl-GPI (20:4)*	1.15	0.88
Lipid	Plasmalogen	1-(1-enyl-palmitoyl)-2-oleoyl-GPE (P-16:0/18:1)*	1.20	0.99
		1-(1-enyl-palmitoyl)-2-linoleoyl-GPE (P-16:0/18:2)*	1.16	0.69
		1-(1-enyl-palmitoyl)-2-palmitoyl-GPC (P-16:0/16:0)*	1.25	1.01
		1-(1-enyl-palmitoyl)-2-palmitoleoyl-GPC (P-16:0/16:1)*	1.16	0.95
		1-(1-enyl-palmitoyl)-2-arachidonoyl-GPE (P-16:0/20:4)*	1.23	0.98
		1-(1-enyl-palmitoyl)-2-oleoyl-GPC (P-16:0/18:1)*	1.20	0.93
		1-(1-enyl-stearoyl)-2-oleoyl-GPE (P-18:0/18:1)	1.41	1.31
		1-(1-enyl-stearoyl)-2-linoleoyl-GPE (P-18:0/18:2)*	1.32	0.84
		1-(1-enyl-palmitoyl)-2-arachidonoyl-GPC (P-16:0/20:4)*	1.19	1.01
		1-(1-enyl-palmitoyl)-2-linoleoyl-GPC (P-16:0/18:2)*	1.29	0.85
		1-(1-enyl-stearoyl)-2-arachidonoyl-GPE (P-18:0/20:4)*	1.56	1.17
	Lysoplasmalogen	1-(1-enyl-palmitoyl)-GPC (P-16:0)*	0.82	0.84
		1-(1-enyl-palmitoyl)-GPE (P-16:0)*	0.76	0.73
		1-(1-enyl-oleoyl)-GPE (P-18:1)*	0.73	0.76
		1-(1-enyl-stearoyl)-GPE (P-18:0)*	0.96	0.88
		1-(1-enyl-oleoyl)-2-oleoyl-GPE (P-18:1/18:1)*	1.01	1.00
	Glycerolipid Metabolism	glycerol	1.08	1.23

		glycerol 3-phosphate	1.03	0.95
		glycerophosphoglycerol	1.13	0.87
	Monoacylglycerol	1-pentadecanoylglycerol (15:0)	0.70	1.05
		1-palmitoylglycerol (16:0)	1.50	0.76
		1-margaroylglycerol (17:0)	1.56	1.06
		1-oleoylglycerol (18:1)	1.16	0.86
		1-linoleoylglycerol (18:2)	0.76	1.09
		2-dihomo-linoleoylglycerol (20:2)*	0.77	0.75
		1-dihomo-linolenylglycerol (20:3)	0.84	**0.51**
		1-arachidonylglycerol (20:4)	0.82	0.97
		2-palmitoylglycerol (16:0)	1.01	0.77
		2-palmitoleoylglycerol (16:1)*	0.57	0.58
		2-oleoylglycerol (18:1)	1.05	0.66
		2-linoleoylglycerol (18:2)	0.77	0.95
		2-arachidonoylglycerol (20:4)	0.76	0.80
	Monoacylglycerol	1-heptadecenoylglycerol (17:1)*	0.66	0.83
		2-heptadecenoylglycerol (17:1)*	0.70	0.88
		1-meadoylglycerol (20:3n9)*	1.70	0.46
		1-dihomo-linoleoylglycerol (20:2)	0.48	0.91
	Diacylglycerol	diacylglycerol (14:0/18:1, 16:0/16:1) [2]*	**0.51**	**0.72**
		diacylglycerol (16:1/18:2 [2], 16:0/18:3 [1])*	0.91	**0.72**
		palmitoyl-oleoyl-glycerol (16:0/18:1) [2]*	**0.80**	**0.83**
		palmitoyl-linoleoyl-glycerol (16:0/18:2) [2]*	1.11	**0.66**
		palmitoleoyl-oleoyl-glycerol (16:1/18:1) [2]*	**0.57**	**0.59**
		palmitoyl-arachidonoyl-glycerol (16:0/20:4) [2]*	1.01	**0.73**
		oleoyl-oleoyl-glycerol (18:1/18:1) [2]*	0.94	1.09
		oleoyl-linoleoyl-glycerol (18:1/18:2) [1]	0.93	0.94
Lipid		oleoyl-linoleoyl-glycerol (18:1/18:2) [2]	0.96	**0.83**
		linoleoyl-linolenoyl-glycerol (18:2/18:3) [2]*	1.33	**0.59**
		stearoyl-arachidonoyl-glycerol (18:0/20:4) [1]*	0.92	0.93
		stearoyl-arachidonoyl-glycerol (18:0/20:4) [2]*	**1.41**	1.02
		oleoyl-arachidonoyl-glycerol (18:1/20:4) [2]*	**1.30**	0.87
	Sphingolipid Synthesis	sphinganine	**0.73**	**0.71**
		sphingadienine	**0.53**	**0.34**
		phytosphingosine	**0.53**	**0.51**
	Dihydroceramides	N-palmitoyl-sphinganine (d18:0/16:0)	0.97	**1.52**
	Ceramides	N-palmitoyl-sphingosine (d18:1/16:0)	**0.78**	**0.73**
		N-stearoyl-sphingosine (d18:1/18:0)*	1.03	**1.21**
		N-palmitoyl-sphingadienine (d18:2/16:0)*	**0.89**	**0.40**
		N-palmitoyl-heptadecasphingosine (d17:1/16:0)*	**0.82**	**0.67**
		ceramide (d18:1/14:0, d16:1/16:0)*	**0.78**	**0.68**
		ceramide (d18:1/17:0, d17:1/18:0)*	**1.19**	1.01
		glycosyl-N-palmitoyl-sphingosine (d18:1/16:0)	0.97	**0.54**

Lipid	Hexosylceramides (HCER)	glycosyl-N-stearoyl-sphingosine (d18:1/18:0)	0.88	**0.66**
		glycosyl ceramide (d18:1/20:0, d16:1/22:0)*	1.04	1.00
		glycosyl ceramide (d18:2/24:1, d18:1/24:2)*	1.06	**0.38**
	Lactosylceramides (LCER)	lactosyl-N-palmitoyl-sphingosine (d18:1/16:0)	**0.86**	**0.63**
		lactosyl-N-nervonoyl-sphingosine (d18:1/24:1)*	0.89	0.86
	Dihydrosphingomyelins	myristoyl dihydrosphingomyelin (d18:0/14:0)*	0.94	**1.67**
		palmitoyl dihydrosphingomyelin (d18:0/16:0)*	1.05	**1.66**
		behenoyl dihydrosphingomyelin (d18:0/22:0)*	1.20	**2.49**
		sphingomyelin (d18:0/18:0, d19:0/17:0)*	1.08	**3.52**
		sphingomyelin (d18:0/20:0, d16:0/22:0)*	1.20	**3.66**
	Sphingomyelins	palmitoyl sphingomyelin (d18:1/16:0)	1.11	1.00
		hydroxypalmitoyl sphingomyelin (d18:1/16:0(OH))**	**1.66**	**1.55**
		stearoyl sphingomyelin (d18:1/18:0)	**1.38**	**1.38**
		behenoyl sphingomyelin (d18:1/22:0)*	1.19	**1.45**
		lignoceroyl sphingomyelin (d18:1/24:0)	**1.23**	**1.34**
	Sphingomyelins	sphingomyelin (d18:2/24:2)*	1.45	0.96
		sphingomyelin (d17:1/14:0, d16:1/15:0)*	0.99	1.15
		sphingomyelin (d18:1/14:0, d16:1/16:0)*	1.04	1.00
		sphingomyelin (d18:2/14:0, d18:1/14:1)*	1.16	0.90
		sphingomyelin (d17:1/16:0, d18:1/15:0, d16:1/17:0)*	1.06	0.98
		sphingomyelin (d18:2/16:0, d18:1/16:1)*	**1.15**	**0.70**
		sphingomyelin (d18:1/17:0, d17:1/18:0, d19:1/16:0)	**1.38**	1.19
		sphingomyelin (d18:1/18:1, d18:2/18:0)	**1.54**	**1.38**
		sphingomyelin (d18:1/20:0, d16:1/22:0)*	**1.20**	**1.34**
		sphingomyelin (d18:1/21:0, d17:1/22:0, d16:1/23:0)*	1.23	0.99
		sphingomyelin (d18:1/22:1, d18:2/22:0, d16:1/24:1)*	1.03	1.08
		sphingomyelin (d18:2/23:0, d18:1/23:1, d17:1/24:1)*	1.09	1.15
		sphingomyelin (d18:1/24:1, d18:2/24:0)*	**1.19**	0.99
		sphingomyelin (d18:2/24:1, d18:1/24:2)*	1.10	1.11
		sphingomyelin (d18:1/25:0, d19:0/24:1, d20:1/23:0, d19:1/24:0)*	**1.44**	1.05
	Ceramide PEs	palmitoyl-sphingosine-phosphoethanolamine (d18:1/16:0)	1.41	1.13
	Sphingosines	sphingosine	**0.51**	**0.45**
		hexadecasphingosine (d16:1)*	**0.59**	**0.40**
		heptadecasphingosine (d17:1)	**0.64**	**0.50**
		eicosanoylsphingosine (d20:1)*	**0.76**	**0.63**
	Mevalonate Metabolism	3-hydroxy-3-methylglutarate	**1.34**	1.09
	Sterol	cholesterol	1.03	1.09
		4-cholesten-3-one	**0.72**	0.77
		7-hydroxycholesterol (alpha or beta)	0.99	0.81
Nucleotide	Purine Metabolism, (Hypo)Xanthine/Inosine containing	AICA ribonucleotide	1.00	**2.03**
		inosine	1.11	1.09
		hypoxanthine	1.00	1.36
		xanthine	0.79	**0.58**

		2'-deoxyinosine	0.04	0.04
		urate	**0.57**	**0.30**
	Purine Metabolism, Adenine containing	adenosine 5'-triphosphate (ATP)	0.83	0.72
		adenosine 5'-monophosphate (AMP)	0.84	0.69
		adenosine 3'-monophosphate (3'-AMP)	0.78	0.57
		adenosine 2'-monophosphate (2'-AMP)	0.82	0.45
		adenosine	1.10	1.08
		adenine	0.27	0.08
		N6-methyladenosine	0.80	1.31
		N6,N6-dimethyladenosine	1.01	**1.48**
		2'-deoxyadenosine	0.01	0.00
	Purine Metabolism, Guanine containing	guanosine 5'- diphosphate (GDP)	0.98	**1.37**
		guanosine 5'- monophosphate (5'-GMP)	0.88	0.65
		guanosine 3'-monophosphate (3'-GMP)	0.75	0.56
		guanosine	1.04	**1.70**
		guanine	**1.85**	**7.93**
	Purine Metabolism, Guanine containing	7-methylguanine	0.89	0.89
		2'-deoxyguanosine	0.04	0.07
	Pyrimidine Metabolism, Orotate containing	orotate	**1.94**	**1.52**
	Pyrimidine Metabolism, Uracil containing	uridine 3'-monophosphate (3'-UMP)	0.90	0.66
		uridine	1.10	1.14
		uracil	0.76	1.38
		pseudouridine	0.79	1.45
		3-ureidopropionate	**0.52**	0.81
Nucleotide	Pyrimidine Metabolism, Cytidine containing	cytidine 5'-monophosphate (5'-CMP)	**1.33**	1.11
		cytidine 2' or 3'-monophosphate (2' or 3'-CMP)	0.63	0.51
		cytidine	0.74	1.09
		cytosine	0.52	0.39
		2'-deoxycytidine 5'-monophosphate	0.05	0.05
		2'-deoxycytidine	0.16	**0.08**
		2'-O-methylcytidine	**0.57**	1.22
	Pyrimidine Metabolism, Thymine containing	thymidine 5'-monophosphate	**0.80**	**0.80**
		thymidine	1.00	1.00
	Purine and Pyrimidine Metabolism	methylphosphate	**0.76**	0.89
Cofactors and Vitamins	Nicotinate and Nicotinamide Metabolism	nicotinamide	0.64	0.97
		nicotinamide ribonucleotide (NMN)	0.83	1.00
		nicotinamide riboside	1.30	0.81
		nicotinamide adenine dinucleotide (NAD+)	0.87	0.81
		1-methylnicotinamide	**0.80**	**0.60**
		adenosine 5'-diphosphoribose (ADP-ribose)	**0.51**	0.73
	Riboflavin Metabolism	riboflavin (Vitamin B2)	**0.68**	**0.76**

		flavin adenine dinucleotide (FAD)	1.00	0.97
	Pantothenate and CoA Metabolism	pantothenate	0.86	**0.86**
		pantetheine	0.99	1.17
		phosphopantetheine	1.03	1.27
	Tocopherol Metabolism	alpha-tocopherol	1.08	**0.89**
		gamma-tocopherol/beta-tocopherol	**0.58**	0.66
	Hemoglobin and Porphyrin Metabolism	heme	**0.45**	0.97
	Thiamine Metabolism	thiamin (Vitamin B1)	**0.61**	1.17
	Vitamin A Metabolism	retinol (Vitamin A)	0.86	0.96
	Vitamin B6 Metabolism	pyridoxine (Vitamin B6)	0.97	0.83
		pyridoxamine	1.24	0.98
		pyridoxamine phosphate	0.95	0.89
		pyridoxal phosphate	**0.74**	1.02
		pyridoxal	0.97	1.20
Xenobiotics	Benzoate Metabolism	hippurate	1.02	0.99
		p-cresol sulfate	**0.79**	0.90
	Food Component/Plant	3-formylindole	0.97	**0.78**
		ergothioneine	**0.43**	1.21
		stachydrine	1.02	1.02
		methyl glucopyranoside (alpha + beta)	1.26	0.94
	Drug - Antibiotic	penicillin G	1.02	0.85
Xenobiotics	Drug - Antineoplastic	tamoxifen	**0.81**	**0.73**
	Chemical	HEPES	1.13	0.91
		2,4-di-tert-butylphenol	0.91	0.93
		phenol red	1.02	0.89
		thioproline	1.19	0.91
		4-chlorobenzoic acid	1.10	**0.80**
