# Supplementary material for: The Δ40p53 isoform inhibits p53-dependent eRNA transcription and enables regulation by signal-specific transcription factors during p53 activation
Source: PLoS Biol. 2021 Aug 5;19(8):e3001364. doi: 10.1371/journal.pbio.3001364 (PMC8370613; doi:10.1371/journal.pbio.3001364)
Supplement: S3 Table — (PDF) [file pbio.3001364.s022.pdf]

Table S3

**Insert verification:**	
TP53exon2-Forward	cagacactggcatggtgttgg
DBD_Forward	tctacaagcagtcacagcac
DBD_Reverse	ttccgtcccagtagattacca
Seq_TP53_exon2_Reverse	cagcccaacccttgtcctta
Seq_TP53_exon2_Forward	gagtgcttgggttgtggtga
CL802	catcttctgtcccttcccagaaaac
CL803	gtgctcgcttagtgctccctg
CRISPR3_seq	tgttcctggctagccaaggaacc
**RT-qPCR primers**	
p21 Forward	ctggagactctcagggtcgaaa
p21 Reverse	gattagggcttcctcttggagaa
PUMA Forward	acgacctcaacgcacagtacg
PUMA Reverse	tcccatgatgagattgtacag
gene desert Forward	tcttcattagtcccaaaaggc
gene desert Reverse	ggaagtttgaagcattagcgagat
