# Supplementary material for: The Δ40p53 isoform inhibits p53-dependent eRNA transcription and enables regulation by signal-specific transcription factors during p53 activation
Source: PLoS Biol. 2021 Aug 5;19(8):e3001364. doi: 10.1371/journal.pbio.3001364 (PMC8370613; doi:10.1371/journal.pbio.3001364)
Supplement: S4 Table — (PDF) [file pbio.3001364.s023.pdf]

TABLE S4

Heat map of statistically significant biochemicals profiled in this study. Red and green shaded cells indicate p≤0.05 (red indicates that the mean values are significantly higher for that comparison; green values significantly lower). Light red and light green shaded cells indicate 0.05<p<0.10 (light red indicates that the mean values trend higher for that comparison; light green values trend lower). For the ANOVA, blue-shaded cells indicate p≤0.05; light blue-shaded cells indicate 0.05<p<0.10

Pathway Sort Order	Super Pathway	Sub Pathway	Biochemical Name	Platform	Comp ID	KEGG	HMDB	PUBCHEM	FLp53
1	Amino Acid	Glycine, Serine and Threonine Metabolism	glycine	LC/MS pos early	58	C00037	HMDB00123	750	0.99
6			betaine	LC/MS pos early	3141	C00719	HMDB00043	247	1.12
9			serine	LC/MS pos early	1648	C00065	HMDB00187	5951	0.96
10			N-acetylserine	LC/MS polar	37076		HMDB02931	65249	1.34
16			threonine	LC/MS pos early	1284	C00188	HMDB00167	6288	0.98
17			N-acetylthreonine	LC/MS neg	33939		HMDB62557	152204	1.52
28		Alanine and Aspartate Metabolism	alanine	LC/MS pos early	1126	C00041	HMDB00161	5950	1.02
30			N-acetylalanine	LC/MS neg	1585	C02847	HMDB00766	88064	1.26
35			aspartate	LC/MS pos early	443	C00049	HMDB00191	5960	1.22
36			N-acetylaspartate (NAA)	LC/MS polar	22185	C01042	HMDB00812	65065	0.80
39			asparagine	LC/MS pos early	512	C00152	HMDB00168	6267	1.04
42		Glutamate Metabolism	glutamate	LC/MS pos early	57	C00025	HMDB00148	611	0.98
43			glutamine	LC/MS pos early	53	C00064	HMDB00641	5961	1.14
45			N-acetylglutamate	LC/MS pos early	15720	C00624	HMDB01138	70914	1.81
46			N-acetylglutamine	LC/MS pos early	33943	C02716	HMDB06029	182230	1.55
51			glutamate, gamma-methyl ester	LC/MS pos early	33487		HMDB61715	68662	1.36
52			pyroglutamine*	LC/MS pos early	46225			134508	1.59
54			beta-citrylglutamate	LC/MS neg	54923	C20775		72715786	1.35
60			S-1-pyrroline-5-carboxylate	LC/MS pos early	42370	C04322	HMDB01301	1196	1.36
67		Histidine Metabolism	histidine	LC/MS neg	59	C00135	HMDB00177	6274	1.09
77			formiminoglutamate	LC/MS pos early	43493	C00439	HMDB00854	439233	0.77
78			imidazole lactate	LC/MS pos early	15716	C05568	HMDB02320	793	1.58
91			4-imidazoleacetate	LC/MS pos early	32349	C02835	HMDB02024	96215	1.59
99		Lysine Metabolism	lysine	LC/MS pos early	1301	C00047	HMDB00182	5962	1.08
101			N6-acetyllysine	LC/MS neg	36752	C02727	HMDB00206	92832	0.96
108			N6-methyllysine	LC/MS pos early	62860	C02728	HMDB02038	164795	0.95
109			N6,N6-dimethyllysine	LC/MS pos early	62862	C05545	HMDB13287	193344	0.94
110			N6,N6,N6-trimethyllysine	LC/MS pos early	1498	C03793	HMDB01325	440120	1.00
113			5-(galactosylhydroxy)-L-lysine	LC/MS pos early	43582			123989	1.60
114			fructosyllysine	LC/MS pos early	46227			123708	1.15
133		Phenylalanine Metabolism	phenylalanine	LC/MS pos early	64	C00079	HMDB00159	6140	1.06
155		Tyrosine Metabolism	tyrosine	LC/MS neg	1299	C00082	HMDB00158	6057	1.01
171			3-(4-hydroxyphenyl)lactate	LC/MS neg	32197	C03672	HMDB00755	9378	1.31
180			phenol sulfate	LC/MS neg	32553	C02180	HMDB60015	74426	1.19
207			O-methyltyrosine	LC/MS pos early	37451			76957	1.06
217			N-formylphenylalanine	LC/MS neg	48433			759256	1.07
240		Tryptophan Metabolism	tryptophan	LC/MS neg	54	C00078	HMDB00929	6305	1.10
252			kynurenine	LC/MS pos early	15140	C00328	HMDB00684	161166	1.31
300		Leucine, Isoleucine and Valine Metabolism	leucine	LC/MS pos early	60			6106	1.02
318			isovalerylcarnitine (C5)	LC/MS pos early	34407		HMDB00688	6426851	0.87
327			beta-hydroxyisovalerate	LC/MS polar	12129		HMDB00754	69362	0.91
347			isoleucine	LC/MS pos early	1125	C00407	HMDB00172	6306	1.07
357			2-methylbutyrylcarnitine (C5)	LC/MS pos early	45095		HMDB00378	6426901	0.89
360			tiglylcarnitine (C5:1-DC)	LC/MS pos early	35428		HMDB02366	22833596	1.33
366			methylsuccinate	LC/MS polar	15745		HMDB01844	10349	0.88
372			valine	LC/MS pos early	1649	C00183	HMDB00883	6287	1.02
379			alpha-hydroxyisovalerate	LC/MS polar	46537		HMDB00407	99823	1.52
381			isobutyrylcarnitine (C4)	LC/MS pos early	33441		HMDB00736	168379	0.99
389		Methionine, Cysteine, SAM and Taurine Metabolism	methionine	LC/MS pos early	1302	C00073	HMDB00696	6137	1.06
390			N-acetylmethionine	LC/MS polar	1589	C02712	HMDB11745	448580	1.05
392			N-formylmethionine	LC/MS neg	2829	C03145	HMDB01015	439750	1.15
395			methionine sulfoxide	LC/MS pos early	18374	C02989	HMDB02005	158980	0.94
396			N-acetylmethionine sulfoxide	LC/MS pos early	45428			193368	1.03
401			S-adenosylhomocysteine (SAH)	LC/MS neg	42382	C00021	HMDB00939	439155	1.09
408			cystathionine	LC/MS pos early	15705	C02291	HMDB00099	439258	1.55
410			cysteine	LC/MS pos early	1868	C00097	HMDB00574	5862	1.22
411			N-acetylcysteine	LC/MS pos early	1586	C06809	HMDB01890	12035	1.12
412			S-methylcysteine	LC/MS pos early	39592		HMDB02108	24417	1.09
417			cystine	LC/MS pos early	56	C00491	HMDB00192	67678	1.37
423			cysteine sulfinic acid	LC/MS pos early	37443	C00606	HMDB00996	109	0.95
425			hypotaurine	LC/MS pos early	590	C00519	HMDB00965	107812	1.77
426			taurine	LC/MS neg	2125	C00245	HMDB00251	1123	1.54
438		Urea cycle; Arginine and Proline Metabolism	arginine	LC/MS pos early	1638	C00062	HMDB00517	232	1.11
439			argininosuccinate	LC/MS pos early	15497	C03406	HMDB00052	16950	1.29
440			urea	LC/MS pos early	1670	C00086	HMDB00294	1176	0.89
442			ornithine	LC/MS pos early	1493	C00077	HMDB03374	6262	1.13
446			2-oxoarginine*	LC/MS pos early	55072	C03771	HMDB04225	558	1.93
447			citrulline	LC/MS pos early	2132	C00327	HMDB00904	9750	0.97
450			proline	LC/MS pos early	1898	C00148	HMDB00162	145742	1.23
453			dimethylarginine (SDMA + ADMA)	LC/MS pos early	36808	C03626	HMDB01539	123831	1.03
467			trans-4-hydroxyproline	LC/MS pos early	32306	C01157	HMDB00725	5810	1.28
475			N-monomethylarginine	LC/MS pos early	43586	C03884	HMDB29416	132862	1.03
481		Creatine Metabolism	guanidinoacetate	LC/MS pos early	43802	C00581	HMDB00128	763	1.09
482			creatine	LC/MS neg	27718	C00300	HMDB00064	586	1.55
483			creatinine	LC/MS pos early	513	C00791	HMDB00562	588	1.28
491		Polyamine Metabolism	putrescine	LC/MS pos early	1408	C00134	HMDB01414	1045	1.47
492			N-acetylputrescine	LC/MS pos early	37496	C02714	HMDB02064	122356	0.78
499			spermidine	LC/MS pos late	485	C00315	HMDB01257	1102	1.35
504			(N(1) + N(8))-acetylspermidine	LC/MS pos early	57814				3.81
506			spermine	LC/MS pos late	603	C00750	HMDB01256	1103	1.12
507			N(1)-acetylspermine	LC/MS pos early	32360	C02567	HMDB01186	916	3.55
510			5-methylthioadenosine (MTA)	LC/MS pos early	1419	C00170	HMDB01173	439176	1.06
517		Guanidino and Acetamido Metabolism	4-guanidinobutanoate	LC/MS pos early	15681	C01035	HMDB03464	500	1.06
520		Glutathione Metabolism	glutathione, reduced (GSH)	LC/MS neg	2127	C00051	HMDB00125	124886	1.76
521			glutathione, oxidized (GSSG)	LC/MS pos early	27727	C00127	HMDB03337	65359	1.24
527			cysteine-glutathione disulfide	LC/MS pos early	35159		HMDB00656	4247235	1.18
533			5-oxoproline	LC/MS neg	1494	C01879	HMDB00267	7405	1.22
586	Peptide	Gamma-glutamyl Amino Acid	gamma-glutamylcysteine	LC/MS pos early	1778	C00669	HMDB01049	842	5.51
588			gamma-glutamylglutamine	LC/MS pos early	2730	C05283	HMDB11738	150914	1.78
592			gamma-glutamylleucine	LC/MS pos early	18369		HMDB11171	151023	1.20
616		Dipeptide	alanylleucine	LC/MS pos early	37093		HMDB28691	259583	1.40
743			glutaminylleucine	LC/MS pos early	42994			18218208	1.00
767			glycylleucine	LC/MS pos early	34398	C02155	HMDB00759	92843	0.91
777			glycylvaline	LC/MS pos early	18357		HMDB28854	97417	1.08
782			histidylalanine	LC/MS pos early	42027		HMDB28878	351667	0.90
801			isoleucylglycine	LC/MS neg	40008		HMDB28907	342532	1.01
815			leucylalanine	LC/MS pos early	40010		HMDB28922	259321	0.78
820			leucylglycine	LC/MS pos early	40045		HMDB28929	79070	0.83
869			phenylalanylalanine	LC/MS neg	41374			5488196	0.82
874			phenylalanylglycine	LC/MS neg	41370		HMDB28995	98207	0.83
895			prolylglycine	LC/MS pos early	40703		HMDB11178	6426709	1.33
934			threonylphenylalanine	LC/MS neg	31530		HMDB29068	4099799	0.79
946			tryptophylglycine	LC/MS pos early	43028		HMDB29083	263471	0.99
963			tyrosylglycine	LC/MS neg	41375		HMDB29105	259323	0.74
980			valylglutamine	LC/MS pos early	42079		HMDB29125	5253209	0.91
981			valylglycine	LC/MS pos early	40475		HMDB29127	136487	0.94
984			valylleucine	LC/MS neg	39994		HMDB29131	352035	0.81
994			leucylglutamine*	LC/MS pos early	48189		HMDB28927	4305457	1.06
1131		Acetylated Peptides	phenylacetylglycine	LC/MS neg	33945	C05598	HMDB00821	68144	1.62
1163	Carbohydrate	Glycolysis, Gluconeogenesis, and Pyruvate Metabolism	glucose	LC/MS polar	20488	C00031	HMDB00122	5793	1.41
1170			fructose 1,6-diphosphate/glucose 1,6-diphosphate/myo-inosit	LC/MS neg	46896				0.92
1172			dihydroxyacetone phosphate (DHAP)	LC/MS pos early	15522	C00111	HMDB01473	668	1.56
1177			3-phosphoglycerate	LC/MS neg	1414	C00597	HMDB00807	724	1.27
1178			phosphoenolpyruvate (PEP)	LC/MS neg	597	C00074	HMDB00263	1005	1.41
1179			pyruvate	LC/MS polar	48990	C00022	HMDB00243	1060	1.34
1180			lactate	LC/MS polar	527	C00186	HMDB00190	612	1.32
1183			glycerate	LC/MS neg	1572	C00258	HMDB00139	752	1.52
1197		Pentose Phosphate Pathway	sedoheptulose-7-phosphate	LC/MS neg	35649	C05382	HMDB01068	616	0.88
1201		Pentose Metabolism	ribose	LC/MS polar	1471	C00121	HMDB00283	5779	1.49
1288		Fructose, Mannose and Galactose Metabolism	mannitol/sorbitol	LC/MS polar	46142	C00794	HMDB00247	5780	0.85
1289			mannose	LC/MS polar	584	C00159	HMDB00169	18950	1.00
1299			galactitol (dulcitol)	LC/MS polar	1117	C01697	HMDB00107	11850	1.24
1318		Nucleotide Sugar	UDP-glucose/UDP-galactose	LC/MS neg	38670				1.88
1320			UDP-glucuronate	LC/MS neg	2763	C00167	HMDB00935	17473	1.36
1327			UDP-N-acetylglucosamine/galactosamine	LC/MS polar	46148				1.03
1346		Aminosugar Metabolism	N-acetylglucosamine 6-phosphate	LC/MS polar	15107	C00357	HMDB02817	439219	1.21
1347			N-acetyl-glucosamine 1-phosphate	LC/MS polar	15741	C04256	HMDB01367	440366	0.91
1353			N-acetylneuraminate	LC/MS pos early	32377	C00270	HMDB00230	439197	0.79
1364			N-acetylglucosaminylasparagine	LC/MS pos early	48149	C04540	HMDB00489	123826	1.24
1365			erythronate*	LC/MS polar	42420		HMDB00613	2781043	1.19
1367			N-acetylglucosamine/N-acetylgalactosamine	LC/MS pos early	46539				1.36
1368			N-glycolylneuraminate	LC/MS pos early	37123	C03410	HMDB00833	123802	0.70
1373	Energy	TCA Cycle	citrate	LC/MS neg	1564	C00158	HMDB00094	311	1.01
1375			aconitate [cis or trans]	LC/MS neg	46173				1.02
1377			isocitrate	LC/MS pos early	12110	C00311	HMDB00193	1198	1.13
1379			alpha-ketoglutarate	LC/MS polar	528	C00026	HMDB00208	51	1.01
1383			fumarate	LC/MS polar	1643	C00122	HMDB00134	444972	1.18
1385			malate	LC/MS polar	1303	C00149	HMDB00156	525	1.23
1398		Oxidative Phosphorylation	acetylphosphate	LC/MS polar	15488	C00227	HMDB01494	186	1.24
1400			phosphate	LC/MS neg	42109	C00009	HMDB01429	1061	1.05
1438		Long Chain Saturated Fatty Acid	myristate (14:0)	LC/MS neg	1365	C06424	HMDB00806	11005	1.18
1440			pentadecanoate (15:0)	LC/MS neg	1361	C16537	HMDB00826	13849	1.08
1441			palmitate (16:0)	LC/MS neg	1336	C00249	HMDB00220	985	1.29
1443			margarate (17:0)	LC/MS neg	1121		HMDB02259	10465	1.21
1445			stearate (18:0)	LC/MS neg	1358	C01530	HMDB00827	5281	1.19
1447			nonadecanoate (19:0)	LC/MS neg	1356	C16535	HMDB00772	12591	1.13
1448			arachidate (20:0)	LC/MS neg	1118	C06425	HMDB02212	10467	1.44
1460		Long Chain Monounsaturated Fatty Acid	palmitoleate (16:1n7)	LC/MS neg	33447	C08362	HMDB03229	445638	1.08
1462			10-heptadecenoate (17:1n7)	LC/MS neg	33971		HMDB60038	5312435	1.13
1464			oleate/vaccenate (18:1)	LC/MS neg	52285				1.19
1471			10-nonadecenoate (19:1n9)	LC/MS neg	33972		HMDB13622	5312513	1.17
1474			eicosenoate (20:1)	LC/MS neg	33587	C16526	HMDB02231	5282768	1.14
1478			erucate (22:1n9)	LC/MS neg	1552	C08316	HMDB02068	5281116	1.02
1481		Long Chain Polyunsaturated Fatty Acid (n3 and n6)	tetradecadienoate (14:2)*	LC/MS neg	62850		HMDB00560	5312402	1.38
1488			stearidonate (18:4n3)	LC/MS neg	33969	C16300	HMDB06547	5312508	1.17
1491			eicosapentaenoate (EPA; 20:5n3)	LC/MS neg	18467	C06428	HMDB01999	446284	0.95
1493			docosapentaenoate (n3 DPA; 22:5n3)	LC/MS neg	32504	C16513	HMDB06528	6441454	1.16
1494			docosahexaenoate (DHA; 22:6n3)	LC/MS neg	44675	C06429	HMDB02183	445580	1.03
1495			docosatrienoate (22:3n3)	LC/MS neg	32417	C16534	HMDB02823	5312556	1.55
1496			nisinate (24:6n3)	LC/MS neg	57810		HMDB02007	11792612	1.06
1497			hexadecadienoate (16:2n6)	LC/MS neg	57652		HMDB00477	13932172	1.39
1498			linoleate (18:2n6)	LC/MS neg	1105	C01595	HMDB00673	5280450	1.05
1500			linolenate [alpha or gamma; (18:3n3 or 6)]	LC/MS neg	34035	C06426	HMDB03073	5280934	1.16
1501			dihomo-linoleate (20:2n6)	LC/MS neg	17805	C16525	HMDB05060	6439848	1.14
1503			dihomo-linolenate (20:3n3 or n6)	LC/MS neg	35718	C03242	HMDB02925	5280581	1.13
1504			arachidonate (20:4n6)	LC/MS neg	1110	C00219	HMDB01043	444899	1.20
1505			docosatrienoate (22:3n6)*	LC/MS neg	57467				1.27
1506			adrenate (22:4n6)	LC/MS neg	32980	C16527	HMDB02226	5497181	1.09
1508			docosadienoate (22:2n6)	LC/MS neg	32415	C16533	HMDB61714	5282807	1.11
1510			mead acid (20:3n9)	LC/MS neg	35174		HMDB10378	5312531	0.92
1572		Fatty Acid, Branched	(14 or 15)-methylpalmitate (a17:0 or i17:0)	LC/MS neg	38768				1.22
1578			(16 or 17)-methylstearate (a19:0 or i19:0)	LC/MS neg	38296		HMDB37397	3083779	1.23
1589		Fatty Acid, Dicarboxylate	glutarate (C5-DC)	LC/MS polar	396	C00489	HMDB00661	743	1.01
1609			azelate (C9-DC)	LC/MS neg	18362	C08261	HMDB00784	2266	0.77
1619			dodecadienoate (12:2)*	LC/MS neg	62276				0.93
1698		Fatty Acid Metabolism (also BCAA Metabolism)	butyrylcarnitine (C4)	LC/MS pos early	32412	C02862	HMDB02013	439829	2.09
1701			propionylcarnitine (C3)	LC/MS pos early	32452	C03017	HMDB00824	107738	0.87
1704			methylmalonate (MMA)	LC/MS polar	1496	C02170	HMDB00202	487	0.99
1765		Fatty Acid Metabolism (Acyl Carnitine, Short Chain)	acetylcarnitine (C2)	LC/MS pos early	32198	C02571	HMDB00201	1	1.42
1787		Fatty Acid Metabolism (Acyl Carnitine, Long Chain Saturated)	palmitoylcarnitine (C16)	LC/MS pos late	44681	C02990	HMDB00222	461	1.72
1789			stearoylcarnitine (C18)	LC/MS pos late	34409		HMDB00848	6426855	1.20
1792			lignoceroylcarnitine (C24)*	LC/MS pos late	57515				0.83
1793			cerotoylcarnitine (C26)*	LC/MS pos late	57516		HMDB06347		0.72
1806		Fatty Acid Metabolism (Acyl Carnitine, Monounsaturated)	oleoylcarnitine (C18:1)	LC/MS pos late	35160		HMDB05065	6441392	1.69
1809			nervonoylcarnitine (C24:1)*	LC/MS pos late	57531				0.71
1810			ximenoylcarnitine (C26:1)*	LC/MS pos late	57517				0.82
1848		Carnitine Metabolism	deoxycarnitine	LC/MS pos early	36747	C01181	HMDB01161	134	1.37
1849			carnitine	LC/MS pos early	15500	C00318	HMDB00062	10917	1.40
1884		Fatty Acid, Monohydroxy	2-hydroxypalmitate	LC/MS neg	35675		HMDB31057	92836	0.59
1886			2-hydroxyoleate	LC/MS neg	54920			9796304	0.64
1887			2-hydroxystearate	LC/MS neg	17945	C03045		69417	0.59
1901			3-hydroxylaurate	LC/MS neg	32457		HMDB00387	94216	1.08
1930			13-HODE + 9-HODE	LC/MS neg	37752				0.86
2064		Endocannabinoid	oleoyl ethanolamide	LC/MS neg	38102		HMDB02088	5283454	1.16
2067			palmitoyl ethanolamide	LC/MS pos late	38165	C16512	HMDB02100	4671	1.20
2068			stearoyl ethanolamide	LC/MS pos late	38625		HMDB13078	27902	1.15
2087			linoleoyl ethanolamide	LC/MS neg	52608		HMDB12252	5283446	1.14
2106		Inositol Metabolism	myo-inositol	LC/MS polar	1124	C00137	HMDB00211	892	1.09
2135		Phospholipid Metabolism	choline	LC/MS pos early	15506	C00114	HMDB00097	305	0.87
2136			choline phosphate	LC/MS pos early	34396	C00588	HMDB01565	1014	0.86
2139			glycerophosphorylcholine (GPC)	LC/MS pos early	15990	C00670	HMDB00086	71920	1.67
2141			phosphoethanolamine	LC/MS neg	1600	C00346	HMDB00224	1015	0.94
2143			glycerophosphoethanolamine	LC/MS polar	37455	C01233	HMDB00114	123874	1.94
2144			glycerophosphoserine*	LC/MS pos early	57404			3081457	1.01
2145			glycerophosphoinositol*	LC/MS polar	47155			167572	0.70
2164		Phosphatidylcholine (PC)	1-myristoyl-2-palmitoyl-GPC (14:0/16:0)	LC/MS pos late	19258		HMDB07869	129657	0.87
2168			1-myristoyl-2-arachidonoyl-GPC (14:0/20:4)*	LC/MS pos late	53195		HMDB07883		0.96
2180			1,2-dipalmitoyl-GPC (16:0/16:0)	LC/MS pos late	19130		HMDB00564	452110	0.92
2181			1-palmitoyl-2-palmitoleoyl-GPC (16:0/16:1)*	LC/MS pos late	52470				0.96
2182			1-palmitoyl-2-stearoyl-GPC (16:0/18:0)	LC/MS pos late	52616		HMDB07970		1.28
2184			1-palmitoyl-2-oleoyl-GPC (16:0/18:1)	LC/MS pos late	52461		HMDB07972	6436017	0.92
2187			1-palmitoyl-2-linoleoyl-GPC (16:0/18:2)	LC/MS pos late	42446		HMDB07973	5287971	0.97
2190			1-palmitoyl-2-gamma-linolenoyl-GPC (16:0/18:3n6)*	LC/MS pos late	54812		HMDB07974		1.09
2198			1-palmitoyl-2-arachidonoyl-GPC (16:0/20:4n6)	LC/MS pos late	52462		HMDB07982	10747814	0.78
2220			1,2-distearoyl-GPC (18:0/18:0)	LC/MS pos late	19132		HMDB08036	94190	1.18
2221			1-stearoyl-2-oleoyl-GPC (18:0/18:1)	LC/MS pos late	52438		HMDB08038		0.88
2232			1-stearoyl-2-arachidonoyl-GPC (18:0/20:4)	LC/MS pos late	42450		HMDB08048	16219824	0.96
2247			1-oleoyl-2-linoleoyl-GPC (18:1/18:2)*	LC/MS pos late	52453				0.95
2257			1-oleoyl-2-docosahexaenoyl-GPC (18:1/22:6)*	LC/MS pos late	52697		HMDB08123		0.83
2258			1,2-dilinoleoyl-GPC (18:2/18:2)	LC/MS pos late	52603		HMDB08138	5288075	0.88
2259			1-linoleoyl-2-linolenoyl-GPC (18:2/18:3)*	LC/MS pos late	53176		HMDB08141		0.75
2301		Phosphatidylethanolamine (PE)	1,2-dipalmitoyl-GPE (16:0/16:0)*	LC/MS pos late	57341		HMDB08923	445468	0.96
2305			1-palmitoyl-2-oleoyl-GPE (16:0/18:1)	LC/MS pos late	19263		HMDB05320	5283496	1.03
2307			1-palmitoyl-2-linoleoyl-GPE (16:0/18:2)	LC/MS pos late	42449		HMDB05322	9546747	0.88
2317			1-palmitoleoyl-2-oleoyl-GPE (16:1/18:1)*	LC/MS pos late	52469				1.13
2322			1-stearoyl-2-oleoyl-GPE (18:0/18:1)	LC/MS pos late	42448		HMDB08993		1.06

2326	Lipid	Phosphatidylethanolamine (PE)	1-stearoyl-2-linoleoyl-GPE (18:0/18:2)*	LC/MS pos late	52446		HMDB08994
2335			1-stearoyl-2-arachidonoyl-GPE (18:0/20:4)	LC/MS pos late	52447		HMDB09003
2340			1,2-dioleoyl-GPE (18:1/18:1)	LC/MS pos late	52609		
2341			1-oleoyl-2-linoleoyl-GPE (18:1/18:2)*	LC/MS pos late	52687		HMDB05349
2344			1-oleoyl-2-arachidonoyl-GPE (18:1/20:4)*	LC/MS pos late	55041		HMDB09069
2396		Phosphatidylserine (PS)	1-palmitoyl-2-oleoyl-GPS (16:0/18:1)	LC/MS pos late	19261	C13880	HMDB12357
2402			1-stearoyl-2-oleoyl-GPS (18:0/18:1)	LC/MS pos late	19265		HMDB10163
2403			1-stearoyl-2-linoleoyl-GPS (18:0/18:2)	LC/MS pos late	53009		
2416		Phosphatidylglycerol (PG)	1-palmitoyl-2-oleoyl-GPG (16:0/18:1)	LC/MS pos late	52448		
2422			1,2-distearoyl-GPG (18:0/18:0)	LC/MS polar	52624		
2423			1-stearoyl-2-oleoyl-GPG (18:0/18:1)	LC/MS polar	52623		
2430		Phosphatidylinositol (PI)	1-palmitoyl-2-oleoyl-GPI (16:0/18:1)*	LC/MS polar	52669		HMDB09783
2435			1-palmitoyl-2-arachidonoyl-GPI (16:0/20:4)*	LC/MS pos late	52467		HMDB09789
2438			1-stearoyl-2-oleoyl-GPI (18:0/18:1)*	LC/MS pos late	52726		
2439			1-stearoyl-2-linoleoyl-GPI (18:0/18:2)	LC/MS pos late	52468		HMDB09809
2441			1,2-dioleoyl-GPI (18:1/18:1)	LC/MS pos late	52619		HMDB09837
2451			1-stearoyl-2-arachidonoyl-GPI (18:0/20:4)	LC/MS pos late	52449		HMDB09815
2456			1-oleoyl-2-arachidonoyl-GPI (18:1/20:4)*	LC/MS polar	54994		
2494		Lysophospholipid	1-palmitoyl-GPC (16:0)	LC/MS pos late	33955		HMDB10382
2495			2-palmitoyl-GPC (16:0)*	LC/MS neg	35253		HMDB61702
2496			1-palmitoleoyl-GPC (16:1)*	LC/MS pos late	33230		HMDB10383
2497			2-palmitoleoyl-GPC (16:1)*	LC/MS pos late	35819		HMDB10383
2503			1-stearoyl-GPC (18:0)	LC/MS pos late	33961		HMDB10384
2505			1-oleoyl-GPC (18:1)	LC/MS neg	48258		HMDB02815
2508			1-linoleoyl-GPC (18:2)	LC/MS neg	34419	C04100	HMDB10386
2540			1-lignoceroyl-GPC (24:0)	LC/MS pos late	49617		HMDB10405
2553			1-palmitoyl-GPE (16:0)	LC/MS pos late	35631		HMDB11503
2560			1-stearoyl-GPE (18:0)	LC/MS pos late	42398		HMDB11130
2561			2-stearoyl-GPE (18:0)*	LC/MS neg	41220		HMDB11129
2562			1-oleoyl-GPE (18:1)	LC/MS neg	35628		HMDB11506
2564			1-linoleoyl-GPE (18:2)*	LC/MS neg	32635		HMDB11507
2577			1-arachidonoyl-GPE (20:4n6)*	LC/MS neg	35186		HMDB11517
2593			1-stearoyl-GPS (18:0)*	LC/MS neg	45966		
2594			1-oleoyl-GPS (18:1)	LC/MS neg	19260		HMDB61694
2599			1-palmitoyl-GPG (16:0)*	LC/MS neg	45970		
2602			1-stearoyl-GPG (18:0)	LC/MS neg	34437		
2604			1-oleoyl-GPG (18:1)*	LC/MS neg	45968		
2606			1-linoleoyl-GPG (18:2)*	LC/MS neg	54885		
2608			1-palmitoyl-GPI (16:0)	LC/MS neg	35305		HMDB61695
2612			1-stearoyl-GPI (18:0)	LC/MS neg	19324		HMDB61696
2614			1-oleoyl-GPI (18:1)	LC/MS neg	36602		
2616			1-linoleoyl-GPI (18:2)*	LC/MS neg	36594		
2622			1-arachidonoyl-GPI (20:4)*	LC/MS neg	34214		HMDB61690
2691		Plasmalogen	1-(1-enyl-palmitoyl)-2-oleoyl-GPE (P-16:0/18:1)*	LC/MS pos late	52477		HMDB11342
2692			1-(1-enyl-palmitoyl)-2-linoleoyl-GPE (P-16:0/18:2)*	LC/MS pos late	52677		HMDB11343
2693			1-(1-enyl-palmitoyl)-2-palmitoyl-GPC (P-16:0/16:0)*	LC/MS pos late	52716		HMDB11206
2694			1-(1-enyl-palmitoyl)-2-palmitoleoyl-GPC (P-16:0/16:1)*	LC/MS pos late	52713		HMDB11207
2695			1-(1-enyl-palmitoyl)-2-arachidonoyl-GPE (P-16:0/20:4)*	LC/MS pos late	52673		HMDB11352
2697			1-(1-enyl-palmitoyl)-2-oleoyl-GPC (P-16:0/18:1)*	LC/MS pos late	52478		
2699			1-(1-enyl-stearoyl)-2-oleoyl-GPE (P-18:0/18:1)	LC/MS pos late	52614		HMDB11375
2700			1-(1-enyl-stearoyl)-2-linoleoyl-GPE (P-18:0/18:2)*	LC/MS pos late	52748		HMDB11376
2703			1-(1-enyl-palmitoyl)-2-arachidonoyl-GPC (P-16:0/20:4)*	LC/MS pos late	52689		HMDB11220
2704			1-(1-enyl-palmitoyl)-2-linoleoyl-GPC (P-16:0/18:2)*	LC/MS pos late	52682		HMDB11211
2708			1-(1-enyl-stearoyl)-2-arachidonoyl-GPE (P-18:0/20:4)*	LC/MS pos late	52475		HMDB05779
2723		Lysoplasmalogen	1-(1-enyl-palmitoyl)-GPC (P-16:0)*	LC/MS pos late	52474		HMDB10407
2724			1-(1-enyl-palmitoyl)-GPE (P-16:0)*	LC/MS pos late	39270		
2726			1-(1-enyl-oleoyl)-GPE (P-18:1)*	LC/MS pos late	44621		
2728			1-(1-enyl-stearoyl)-GPE (P-18:0)*	LC/MS pos late	39271		
2729			1-(1-enyl-oleoyl)-2-oleoyl-GPE (P-18:1/18:1)*	LC/MS pos late	54691		HMDB11441
2731		Glycerolipid Metabolism	glycerol	LC/MS neg	15122	C00116	HMDB00131
2732			glycerol 3-phosphate	LC/MS neg	43847	C00093	HMDB00126
2737			glycerophosphoglycerol	LC/MS polar	48857	C03274	
2740		Monoacylglycerol	1-pentadecanoylglycerol (15:0)	LC/MS neg	47898		
2741			1-palmitoylglycerol (16:0)	LC/MS neg	21127		HMDB31074
2743			1-margaroylglycerol (17:0)	LC/MS neg	34391		
2745			1-oleoylglycerol (18:1)	LC/MS neg	21184		HMDB11567
2746			1-linoleoylglycerol (18:2)	LC/MS neg	27447		
2750			2-dihomo-linoleoylglycerol (20:2)*	LC/MS neg	62450		
2751			1-dihomo-linolenylglycerol (20:3)	LC/MS neg	48341		
2752			1-arachidonylglycerol (20:4)	LC/MS neg	34397	C13857	HMDB11549
2758			2-palmitoylglycerol (16:0)	LC/MS neg	33419		HMDB11533
2759			2-palmitoleoylglycerol (16:1)*	LC/MS neg	52432		
2761			2-oleoylglycerol (18:1)	LC/MS neg	21232		HMDB11537
2762			2-linoleoylglycerol (18:2)	LC/MS neg	32506		HMDB11538
2763			2-arachidonoylglycerol (20:4)	LC/MS neg	19266	C13856	HMDB04666
2767			1-heptadecenoylglycerol (17:1)*	LC/MS neg	62449		
2768			2-heptadecenoylglycerol (17:1)*	LC/MS neg	62448		
2769			1-meadoylglycerol (20:3n9)*	LC/MS neg	62431		
2774			1-dihomo-linoleoylglycerol (20:2)	LC/MS neg	35103		
2778		Diacylglycerol	diacylglycerol (14:0/18:1, 16:0/16:1) [2]*	LC/MS pos late	54954		
2779			diacylglycerol (16:1/18:2 [2], 16:0/18:3 [1])*	LC/MS pos late	54966		
2795			palmitoyl-oleoyl-glycerol (16:0/18:1) [2]*	LC/MS pos late	54942		
2797			palmitoyl-linoleoyl-glycerol (16:0/18:2) [2]*	LC/MS pos late	52634		
2800			palmitoleoyl-oleoyl-glycerol (16:1/18:1) [2]*	LC/MS pos late	52631		
2804			palmitoyl-arachidonoyl-glycerol (16:0/20:4) [2]*	LC/MS pos late	54958		
2814			oleoyl-oleoyl-glycerol (18:1/18:1) [2]*	LC/MS pos late	54946		
2816			oleoyl-linoleoyl-glycerol (18:1/18:2) [1]	LC/MS pos late	46798		HMDB07219
2817			oleoyl-linoleoyl-glycerol (18:1/18:2) [2]	LC/MS pos late	46799		HMDB07219
2824			linoleoyl-linolenoyl-glycerol (18:2/18:3) [2]*	LC/MS pos late	54964		
2827			stearoyl-arachidonoyl-glycerol (18:0/20:4) [1]*	LC/MS pos late	57450		
2828			stearoyl-arachidonoyl-glycerol (18:0/20:4) [2]*	LC/MS pos late	57449		
2830			oleoyl-arachidonoyl-glycerol (18:1/20:4) [2]*	LC/MS pos late	54961		
2890		Sphingolipid Synthesis	sphinganine	LC/MS pos late	17769	C00836	HMDB00269
2892			sphingadienine	LC/MS pos late	57426		
2893			phytosphingosine	LC/MS pos late	1510	C12144	HMDB04610
2896		Dihydroceramides	N-palmitoyl-sphinganine (d18:0/16:0)	LC/MS pos late	52604		HMDB11760
2904		Ceramides	N-palmitoyl-sphingosine (d18:1/16:0)	LC/MS pos late	44877		HMDB04949
2908			N-stearoyl-sphingosine (d18:1/18:0)*	LC/MS pos late	54979		HMDB04950
2914			N-palmitoyl-sphingadienine (d18:2/16:0)*	LC/MS pos late	57416		
2926			N-palmitoyl-heptadecasphingosine (d17:1/16:0)*	LC/MS pos late	57430		
2930			ceramide (d18:1/14:0, d16:1/16:0)*	LC/MS pos late	57432		
2931			ceramide (d18:1/17:0, d17:1/18:0)*	LC/MS pos late	57434		
2939		Hexosylceramides (HCER)	glycosyl-N-palmitoyl-sphingosine (d18:1/16:0)	LC/MS pos late	53013		
2940			glycosyl-N-stearoyl-sphingosine (d18:1/18:0)	LC/MS pos late	52234		
2960			glycosyl ceramide (d18:1/20:0, d16:1/22:0)*	LC/MS pos late	57595		
2964			glycosyl ceramide (d18:2/24:1, d18:1/24:2)*	LC/MS pos late	57453		
2969		Lactosylceramides (LCER)	lactosyl-N-palmitoyl-sphingosine (d18:1/16:0)	LC/MS pos late	53010		
2973			lactosyl-N-nervonoyl-sphingosine (d18:1/24:1)*	LC/MS pos late	57370		
2980		Dihydrosphingomyelins	myristoyl dihydrosphingomyelin (d18:0/14:0)*	LC/MS pos late	57365		HMDB12085
2981			palmitoyl dihydrosphingomyelin (d18:0/16:0)*	LC/MS pos late	52434		
2982			behenoyl dihydrosphingomyelin (d18:0/22:0)*	LC/MS pos late	57331		HMDB12091
2983			sphingomyelin (d18:0/18:0, d19:0/17:0)*	LC/MS pos late	57473		HMDB12087
2984			sphingomyelin (d18:0/20:0, d16:0/22:0)*	LC/MS pos late	57476		
2985		Sphingomyelins	palmitoyl sphingomyelin (d18:1/16:0)	LC/MS pos late	37506		
2986			hydroxypalmitoyl sphingomyelin (d18:1/16:0(OH))**	LC/MS pos late	62851		
2987			stearoyl sphingomyelin (d18:1/18:0)	LC/MS pos late	19503	C00550	HMDB01348
2988			behenoyl sphingomyelin (d18:1/22:0)*	LC/MS pos late	48492		HMDB12103
2990			lignoceroyl sphingomyelin (d18:1/24:0)	LC/MS pos late	57330		
2993			sphingomyelin (d18:2/24:2)*	LC/MS pos late	57479		
2994			sphingomyelin (d17:1/14:0, d16:1/15:0)*	LC/MS pos late	62152		
2995			sphingomyelin (d18:1/14:0, d16:1/16:0)*	LC/MS polar	42463		HMDB12097
2996			sphingomyelin (d18:2/14:0, d18:1/14:1)*	LC/MS pos late	47154		
2997			sphingomyelin (d17:1/16:0, d18:1/15:0, d16:1/17:0)*	LC/MS pos late	52433		
2999			sphingomyelin (d18:2/16:0, d18:1/16:1)*	LC/MS polar	42459		
3000			sphingomyelin (d18:1/17:0, d17:1/18:0, d19:1/16:0)	LC/MS pos late	52615		
3001			sphingomyelin (d18:1/18:1, d18:2/18:0)	LC/MS pos late	37529		HMDB12101
3003			sphingomyelin (d18:1/20:0, d16:1/22:0)*	LC/MS pos late	48490		HMDB12102
3006			sphingomyelin (d18:1/21:0, d17:1/22:0, d16:1/23:0)*	LC/MS pos late	52495		
3008			sphingomyelin (d18:1/22:1, d18:2/22:0, d16:1/24:1)*	LC/MS pos late	48493		HMDB12104
3010			sphingomyelin (d18:2/23:0, d18:1/23:1, d17:1/24:1)*	LC/MS pos late	52435		
3011			sphingomyelin (d18:1/24:1, d18:2/24:0)*	LC/MS polar	47153		HMDB12107
3012			sphingomyelin (d18:2/24:1, d18:1/24:2)*	LC/MS pos late	52437		
3013			sphingomyelin (d18:1/25:0, d19:0/24:1, d20:1/23:0, d19:1/24:0)*	LC/MS pos late	57478		
3016		Ceramide PEs	palmitoyl-sphingosine-phosphoethanolamine (d18:1/16:0)	LC/MS pos late	62984		
3041		Sphingosines	sphingosine	LC/MS pos late	17747	C00319	HMDB00252
3049			hexadecasphingosine (d16:1)*	LC/MS pos late	57428		
3050			heptadecasphingosine (d17:1)	LC/MS pos late	57427		
3051			eicosanoylsphingosine (d20:1)*	LC/MS pos late	57597		
3059		Mevalonate Metabolism	3-hydroxy-3-methylglutarate	LC/MS polar	531	C03761	HMDB00355
3075		Sterol	cholesterol	LC/MS pos late	63	C00187	HMDB00067
3101			4-cholesten-3-one	LC/MS pos late	38125	C00599	HMDB00921
3122			7-hydroxycholesterol (alpha or beta)	LC/MS pos late	47890		HMDB06119
3979	Nucleotide	Purine Metabolism, (Hypo)Xanthine/Inosine containing	AICA ribonucleotide	LC/MS pos early	38325	C04677	HMDB01517
3984			inosine	LC/MS pos early	1123	C00294	HMDB00195
3985			hypoxanthine	LC/MS neg	3127	C00262	HMDB00157
3986			xanthine	LC/MS neg	3147	C00385	HMDB00292
3992			2'-deoxyinosine	LC/MS neg	15076	C05512	HMDB00071
3995			urate	LC/MS neg	1604	C00366	HMDB00289
4001		Purine Metabolism, Adenine containing	adenosine 5'-triphosphate (ATP)	LC/MS neg	33449	C00002	HMDB00538
4003			adenosine 5'-monophosphate (AMP)	LC/MS pos early	32342	C00020	HMDB00045
4004			adenosine 3'-monophosphate (3'-AMP)	LC/MS neg	35142	C01367	HMDB03540
4005			adenosine 2'-monophosphate (2'-AMP)	LC/MS neg	36815	C00946	HMDB11617
4010			adenosine	LC/MS neg	555	C00212	HMDB00050
4011			adenine	LC/MS pos early	554	C00147	HMDB00034
4018			N6-methyladenosine	LC/MS pos early	37114		HMDB04044
4019			N6,N6-dimethyladenosine	LC/MS pos early	42081		
4033			2'-deoxyadenosine	LC/MS pos early	1553	C00559	HMDB00101
4040		Purine Metabolism, Guanine containing	guanosine 5'- diphosphate (GDP)	LC/MS neg	2848	C00035	HMDB01201
4041			guanosine 5'- monophosphate (5'-GMP)	LC/MS pos early	2849	C00144	HMDB01397
4042			guanosine 3'-monophosphate (3'-GMP)	LC/MS neg	39786		
4046			guanosine	LC/MS pos early	1573	C00387	HMDB00133
4047			guanine	LC/MS neg	32352	C00242	HMDB00132
4050			7-methylguanine	LC/MS pos early	35114	C02242	HMDB00897
4064			2'-deoxyguanosine	LC/MS neg	1411	C00330	HMDB00085
4070		Pyrimidine Metabolism, Orotate containing	orotate	LC/MS polar	1505	C00295	HMDB00226
4079		Pyrimidine Metabolism, Uracil containing	uridine 3'-monophosphate (3'-UMP)	LC/MS polar	39784	C01368	
4082			uridine	LC/MS polar	606	C00299	HMDB00296
4083			uracil	LC/MS polar	605	C00106	HMDB00300
4084			pseudouridine	LC/MS neg	33442	C02067	HMDB00767
4101			3-ureidopropionate	LC/MS pos early	3155	C02642	HMDB00026
4109		Pyrimidine Metabolism, Cytidine containing	cytidine 5'-monophosphate (5'-CMP)	LC/MS pos early	2372	C00055	HMDB00095
4111			cytidine 2' or 3'-monophosphate (2' or 3'-CMP)	LC/MS pos early	61705		
4113			cytidine	LC/MS pos early	514	C00475	HMDB00089
4114			cytosine	LC/MS pos early	573	C00380	HMDB00630
4121			2'-deoxycytidine 5'-monophosphate	LC/MS pos early	533	C00239	HMDB01202
4123			2'-deoxycytidine	LC/MS pos early	15949	C00881	HMDB00014
4124			2'-O-methylcytidine	LC/MS pos early	57554		
4130		Pyrimidine Metabolism, Thymine containing	thymidine 5'-monophosphate	LC/MS neg	12023	C00364	HMDB01227
4134			thymidine	LC/MS polar	2183	C00214	HMDB00273
4141		Purine and Pyrimidine Metabolism	methylphosphate	LC/MS pos early	37070		HMDB61711
4161	Cofactors and Vitamins	Nicotinate and Nicotinamide Metabolism	nicotinamide	LC/MS pos early	594	C00153	HMDB01406
4163			nicotinamide ribonucleotide (NMN)	LC/MS pos early	22152	C00455	HMDB00229
4164			nicotinamide riboside	LC/MS pos early	33013	C03150	HMDB00855
4165			nicotinamide adenine dinucleotide (NAD+)	LC/MS neg	5278	C00003	HMDB00902
4173			1-methylnicotinamide	LC/MS pos early	27665	C02918	HMDB00699
4183			adenosine 5'-diphosphoribose (ADP-ribose)	LC/MS pos early	558	C00301	HMDB01178
4187		Riboflavin Metabolism	riboflavin (Vitamin B2)	LC/MS pos early	1827	C00255	HMDB00244
4188			flavin adenine dinucleotide (FAD)	LC/MS neg	2134	C00016	HMDB01248
4191		Pantothenate and CoA Metabolism	pantothenate	LC/MS pos early	1508	C00864	HMDB00210
4193			pantetheine	LC/MS neg	57555	C00831	
4194			phosphopantetheine	LC/MS neg	15504	C01134	HMDB01416
4214		Tocopherol Metabolism	alpha-tocopherol	LC/MS pos late	1561	C02477	HMDB01893
4236			gamma-tocopherol/beta-tocopherol	LC/MS pos late	52473		
4260		Hemoglobin and Porphyrin Metabolism	heme	LC/MS pos late	41754	C00032	HMDB03178
4273		Thiamine Metabolism	thiamin (Vitamin B1)	LC/MS pos early	5341	C00378	HMDB00235
4282		Vitamin A Metabolism	retinol (Vitamin A)	LC/MS pos late	1806	C00473	HMDB00305
4298		Vitamin B6 Metabolism	pyridoxine (Vitamin B6)	LC/MS neg	608	C00314	HMDB02075
4300			pyridoxamine	LC/MS pos early	2150	C00534	HMDB01431
4301			pyridoxamine phosphate	LC/MS pos early	3138	C00647	HMDB01555
4302			pyridoxal phosphate	LC/MS neg	5331	C00018	HMDB01491
4303			pyridoxal	LC/MS pos early	1651	C00250	HMDB01545
4311	Xenobiotics	Benzoate Metabolism	hippurate	LC/MS neg	15753	C01586	HMDB00714
4394			p-cresol sulfate	LC/MS neg	36103		HMDB11635
4596		Food Component/Plant	3-formylindole	LC/MS pos early	62863	C08493	HMDB29737
4702			ergothioneine	LC/MS pos early	37459	C05570	HMDB03045
4869			stachydrine	LC/MS pos early	34384	C10172	HMDB04827
4895			methyl glucopyranoside (alpha + beta)	LC/MS pos early	46144		
5210		Drug - Antibiotic	penicillin G	LC/MS neg	37468	C05551	HMDB15186
5347		Drug - Antineoplastic	tamoxifen	LC/MS pos late	64205	D08559 C07108	HMDB14813
5790		Chemical	HEPES	LC/MS neg	21248		
5799			2,4-di-tert-butylphenol	LC/MS neg	64328		
5810			phenol red	LC/MS neg	36817	C12600	
5857			thioproline	LC/MS pos early	53231		
5884			4-chlorobenzoic acid	LC/MS neg	63988		
