# Supplementary material for: The Δ40p53 isoform inhibits p53-dependent eRNA transcription and enables regulation by signal-specific transcription factors during p53 activation
Source: PLoS Biol. 2021 Aug 5;19(8):e3001364. doi: 10.1371/journal.pbio.3001364 (PMC8370613; doi:10.1371/journal.pbio.3001364)
Supplement: S5 Table — RNA-seq, RNA sequencing. (PDF) [file pbio.3001364.s024.pdf]

Table S5

RNA-seq Sample	Unpaired Total	Unpaired Aligned None	Unpaired Aligned One	Unpaired Aligned Multi	Overall Alignment Rate
**WTp53 20hr 0.1% DMSO Rep1**	33545273	196946	28420147	4928180	99.41
**WTp53 20hr 0.1% DMSO Rep2**	27452071	163016	22731675	4557380	99.41
**WTp53 20hr 10µM Nutlin3a Rep1**	30973712	185580	26018909	4769223	99.4
**WTp53 20hr 10µM Nutlin3a Rep2**	33221791	264667	27540238	5416886	99.2
**WTp53:WTp53 20hr 0.1% DMSO Rep1**	27608005	165898	23471504	3970603	99.4
**WTp53:WTp53 20hr 0.1% DMSO Rep2**	31739941	230384	26227061	5282496	99.27
**WTp53:WTp53 20hr 10µM Nutlin3a Rep1**	28456024	173100	24161330	4121594	99.39
**WTp53:WTp53 20hr 10µM Nutlin3a Rep2**	32733735	262404	27024386	5446945	99.2
**Δ40p53:WTp53 20hr 0.1% DMSO Rep1**	28203812	198272	23720840	4284700	99.3
**Δ40p53:WTp53 20hr 0.1% DMSO Rep2**	35581234	233633	29368862	5978739	99.34
**Δ40p53:WTp53 20hr 10µM Nutlin3a Rep1**	30297515	256164	25388553	4652798	99.15
**Δ40p53:WTp53 20hr 10µM Nutlin3a Rep2**	33048045	230934	27661790	5155321	99.3
**WTp53 20hr 0.375% DMSO Rep1**	42513309	456665	35580518	6476126	98.93
**WTp53 20hr 0.375% DMSO Rep2**	42731104	920011	35738807	6072286	97.85
**WTp53 20hr 375µM 5FU Rep1**	38398122	271042	32338112	5788968	99.29
**WTp53 20hr 375µM 5FU Rep2**	42816292	689125	35632252	6494915	98.39
**WTp53:WTp53 20hr 0.375% DMSO Rep1**	44950485	323166	37747665	6879654	99.28
**WTp53:WTp53 20hr 0.375% DMSO Rep2**	40310906	766608	33966099	5578199	98.1
**WTp53:WTp53 20hr 375µM 5FU Rep1**	33625259	540093	28344225	4740941	98.39
**WTp53:WTp53 20hr 375µM 5FU Rep2**	39811189	282054	33458388	6070747	99.29
**Δ40p53:WTp53 20hr 0.375% DMSO Rep1**	77803414	613785	65241948	11947681	99.21
**Δ40p53:WTp53 20hr 0.375% DMSO Rep2**	43373061	431864	36571186	6370011	99
**Δ40p53:WTp53 20hr 375µM 5FU Rep1**	42059915	1502177	34499313	6058425	96.43
**Δ40p53:WTp53 20hr 375µM 5FU Rep2**	40252973	318492	33979467	5955014	99.21
