# Supplementary material for: The Δ40p53 isoform inhibits p53-dependent eRNA transcription and enables regulation by signal-specific transcription factors during p53 activation
Source: PLoS Biol. 2021 Aug 5;19(8):e3001364. doi: 10.1371/journal.pbio.3001364 (PMC8370613; doi:10.1371/journal.pbio.3001364)
Supplement: S6 Table — ChIP-seq, chromatin immunoprecipitation sequencing. (PDF) [file pbio.3001364.s025.pdf]

Table S6

ChIP-seq Sample	Unpaired Total	Unpaired Aligned None	Unpaired Aligned One	Unpaired Aligned Multi	Overall Alignment Rate
**WTp53 6hr 0.1% DMSO Rep1 input**	27515263	1954813	23967127	1593323	92.9
**WTp53 6hr 0.1% DMSO Rep2 input**	13121030	895543	11533483	692004	93.17
**WTp53 6hr 0.1% DMSO Rep1**	26701982	2107545	23207965	1386472	92.11
**WTp53 6hr 0.1% DMSO Rep2**	40782091	3418960	35305333	2057798	91.62
**WTp53 6hr 10µM Nutlin Rep1 input**	25883537	1718248	22748404	1416885	93.36
**WTp53 6hr 10µM Nutlin Rep2 input**	12464099	851199	10952994	659906	93.17
**WTp53 6hr 10µM Nutlin Rep1**	25680387	2202392	22226705	1251290	91.42
**WTp53 6hr 10µM Nutlin Rep2**	18007747	1421340	15702407	884000	92.11
**WTp53:WTp53 6hr 0.1% DMSO Rep1 input**	20921869	1349435	18516757	1055677	93.55
**WTp53WTp53 6hr 0.1% DMSO Rep2 input**	8938265	603241	7875663	459361	93.25
**WTp53WTp53 6hr 0.1% DMSO Rep3 input**	54739173	4494600	47420499	2824074	91.79
**WTp53:WTp53 6hr 0.1% DMSO Rep1**	16124135	1182224	14146252	795659	92.67
**WTp53WTp53 6hr 0.1% DMSO Rep2**	8677149	763657	7491464	422028	91.2
**WTp53WTp53 6hr 0.1% DMSO Rep3**	36750144	4807992	30149867	1792285	86.92
**WTp53:WTp53 6hr 10µM Nutlin Rep1 input**	11059670	1526475	8952902	580293	86.2
**WTp53:WTp53 6hr 10µM Nutlin Rep2 input**	10041034	626407	8937467	477160	93.76
**WTp53:WTp53 6hr 10µM Nutlin Rep3 input**	15164316	14823926	309729	30661	2.24
**WTp53:WTp53 6hr 10µM Nutlin Rep1**	12528553	965626	10932088	630839	92.29
**WTp53:WTp53 6hr 10µM Nutlin Rep2**	37831766	3311011	32870593	1650162	91.25
**WTp53:WTp53 6hr 10µM Nutlin Rep3**	40030698	5049703	32991331	1989664	87.39
**Δ40p53:WTp53 6hr 0.1% DMSO Rep1 input**	10435537	649641	9278411	507485	93.77
**Δ40p53WTp53 6hr 0.1% DMSO Rep2 input**	10646401	616854	9545021	484526	94.21
**Δ40p53WTp53 6hr 0.1% DMSO Rep3 input**	41234243	3863467	35247425	2123351	90.63
**Δ40p53:WTp53 6hr 0.1% DMSO Rep1**	9018545	685902	7897392	435251	92.39
**Δ40p53WTp53 6hr 0.1% DMSO Rep2**	8661718	943735	7208614	509369	89.1
**Δ40p53WTp53 6hr 0.1% DMSO Rep3**	32256642	3729345	26939315	1587982	88.44
**Δ40p53:WTp53 6hr 10µM Nutlin Rep1 input**	4791821	800996	3568419	422406	83.28
**Δ40p53:WTp53 6hr 10µM Nutlin Rep2 input**	9762937	589113	8732757	441067	93.97
**Δ40p53:WTp53 6hr 10µM Nutlin Rep3 input**	50386004	4580640	43041996	2763368	90.91
**Δ40p53:WTp53 6hr 10µM Nutlin Rep1**	11932500	1033480	10279952	619068	91.34
**Δ40p53:WTp53 6hr 10µM Nutlin Rep2**	25265548	6332020	17900028	1033500	74.94
**Δ40p53:WTp53 6hr 10µM Nutlin Rep3**	51518813	5956452	42890367	2671994	88.44
