# Supplementary material for: The Δ40p53 isoform inhibits p53-dependent eRNA transcription and enables regulation by signal-specific transcription factors during p53 activation
Source: PLoS Biol. 2021 Aug 5;19(8):e3001364. doi: 10.1371/journal.pbio.3001364 (PMC8370613; doi:10.1371/journal.pbio.3001364)
Supplement: S7 Table — PRO-seq, precision nuclear run-on sequencing. (PDF) [file pbio.3001364.s026.pdf]

**Table S7**

| PRO-seq Sample (MCF10A) | Unpaired Total | Unpaired Aligned None | Unpaired Aligned One | Unpaired Aligned Multi | Overall Alignment Rate |
|---|---|---|---|---|---|
| **WTp53 3hr 0.1% DMSO Rep1** | 65527109 | 10020610 | 30074735 | 25431764 | 84.71 |
| **WTp53 3hr 0.1% DMSO Rep2** | 48897708 | 8795166 | 20163321 | 19939221 | 82.01 |
| **WTp53 3hr 10µM Nutlin3a Rep1** | 88210845 | 11433535 | 37031932 | 39745378 | 87.04 |
| **WTp53 3hr 10µM Nutlin3a Rep2** | 80233524 | 11046059 | 33407201 | 35780264 | 86.23 |
| **WTp53:WTp53 3hr 0.1% DMSO Rep1** | 63444081 | 10489987 | 29655255 | 23298839 | 83.47 |
| **WTp53:WTp53 3hr 0.1% DMSO Rep2** | 62517988 | 10548246 | 29365790 | 22603952 | 83.13 |
| **WTp53:WTp53 3hr 10µM Nutlin3a Rep1** | 73545769 | 9192417 | 31979150 | 32374202 | 87.5 |
| **WTp53:WTp53 3hr 10µM Nutlin3a Rep2** | 83526928 | 10717016 | 39816036 | 32993876 | 87.17 |
| **Δ40p53:WTp53 3hr 0.1% DMSO Rep1** | 63407244 | 9447642 | 28349844 | 25609758 | 85.1 |
| **Δ40p53:WTp53 3hr 0.1% DMSO Rep2** | 69807930 | 10093051 | 30269415 | 29445464 | 85.54 |
| **Δ40p53:WTp53 3hr 10µM Nutlin3a Rep1** | 113703328 | 11965250 | 53965911 | 47772167 | 89.48 |
| **Δ40p53:WTp53 3hr 10µM Nutlin3a Rep2** | 130135659 | 11159151 | 61477830 | 57498678 | 91.42 |
| **p53 Null 3hr DMSO Rep1** | 44262352 | 4648602 | 24618032 | 14995718 | 89.5 |
| **p53 Null 3hr DMSO Rep2** | 48843419 | 2194980 | 25237703 | 21410736 | 95.51 |
| **p53 Null 3hr Nutlin Rep1** | 52855524 | 3655853 | 23150789 | 26048882 | 93.08 |
| **p53 Null 3hr Nutlin Rep2** | 54991956 | 2073885 | 30136046 | 22782025 | 96.23 |
